# Topic modeling revisited:  New evidence on algorithm performance and quality metrics

**DOI:** 10.1371/journal.pone.0266325

**Published:** 2022-04-28

**Authors:** Matthias Rüdiger, David Antons, Amol M. Joshi, Torsten-Oliver Salge

**Affiliations:** 1 TIME Research Area, School of Business and Economics, RWTH Aachen University, Aachen, Germany; 2 Strategy and Entrepreneurship Area, School of Business, Wake Forest University, Winston-Salem, NC, United States of America; University of Sao Paulo, BRAZIL

## Abstract

Topic modeling is a popular technique for exploring large document collections. It has proven useful for this task, but its application poses a number of challenges. First, the comparison of available algorithms is anything but simple, as researchers use many different datasets and criteria for their evaluation. A second challenge is the choice of a suitable metric for evaluating the calculated results. The metrics used so far provide a mixed picture, making it difficult to verify the accuracy of topic modeling outputs. Altogether, the choice of an appropriate algorithm and the evaluation of the results remain unresolved issues. Although many studies have reported promising performance by various topic models, prior research has not yet systematically investigated the validity of the outcomes in a comprehensive manner, that is, using more than a small number of the available algorithms and metrics. Consequently, our study has two main objectives. First, we compare all commonly used, non-application-specific topic modeling algorithms and assess their relative performance. The comparison is made against a known clustering and thus enables an unbiased evaluation of results. Our findings show a clear ranking of the algorithms in terms of accuracy. Secondly, we analyze the relationship between existing metrics and the known clustering, and thus objectively determine under what conditions these algorithms may be utilized effectively. This way, we enable readers to gain a deeper understanding of the performance of topic modeling techniques and the interplay of performance and evaluation metrics.

## Introduction

The tremendous increase in the volume and variety of unstructured text documents represents a major challenge for social sciences research today. Most text analysis is still based on labor-intensive human coding or dictionary-based methods, which are semi-automated but involve a large amount of manual labor as well. To overcome this problem, social scientists are increasingly incorporating computer-assisted text analysis techniques into their research toolboxes. As a result, the rise of computer-assisted text analysis techniques in the social sciences is driving the emergence of the field of computational social sciences [1]. Qualitative researchers may find automated text analysis to be especially useful, as their work often relies heavily on labor-intensive, manual coding procedures [2]. Quantitative researchers may find that these methods expand their ability to generate new variables that capture the critical dimensions of important theoretical constructs (for an example, see [3]).

Topic modeling is one particular area of application of text mining techniques. Topic models extract theme-level relations by assuming that a single document covers a small set of concise topics based on the words used within the document. Thus, a topic model is able to produce a succinct overview of the themes covered in a document collection as well as the topic distribution of every document within the collection. A topic links a group of similar documents and consequently, the term *topic* corresponds to the often used term *cluster* in data mining. We use the two terms “text clustering” and “topic modeling” interchangeably in this paper. The topics or clusters revealed through topic modeling often correlate well with human understanding of the texts [4]. Many scholars proposed methods to extract hidden topic structures from document collections, among which the most frequently used is Latent Dirichlet Allocation (LDA), the successor of Probabilistic Latent Sematic Analysis (PLSA) [5, 6]. Areas of application include the automated analysis of all kinds of textual data. For example, scholars have uncovered statistical patterns of crime rates in China’s eighteenth and nineteenth centuries [7], developed measures for identifying patents that originate new technologies [8], mapped the topic landscape of academic papers [9], and examined Twitter posts and social media content generated by Internet trolls in the 2016 U.S. elections [10]. Hence, topic models are potentially useful in a wide array of social science research domains as they enable users to organize and summarize text collections at a scale that would be impossible using only human annotation [4].

Given the similarity with data mining and clustering, it is no surprise that the use of topic models comes with a number of challenges typical for the application of unsupervised learning methods. In this application context, there are two main challenges to be met. The first and foremost challenge is the choice of an appropriate algorithm, as numerous variants exist. In general, these methods can be divided into two groups, each with a number of popular representatives classified as either non-probabilistic or probabilistic algorithms. Performance investigations are typically limited to the analysis of one representative of each class or to the comparisons of a number of algorithms within one class. Additionally, researchers rely on different datasets and evaluation criteria when it comes to testing these algorithms. Thus, the comparison of methods may prove inconsistent and sometimes even puzzling. Since no comprehensive, side-by-side comparison is available, researchers lack appropriate evidence-based guidelines for choosing which algorithms to use and when and how to use them [11]. The second challenge is to choose a suitable evaluation metric for the calculated results. Social scientists, for instance, are accustomed to developing survey constructs and scales that apply measures of internal consistency or reliability such as Cronbach’s Alpha. In topic modeling, most algorithms require researchers to specify the number of clusters to be discovered. However, this number is not easily determined in advance. Hence, researchers must utilize trial and error approaches by first specifying many different numbers of clusters and then selecting the best results afterwards. Yet the evaluation criteria that scholars rely upon to date are ill-defined and inconclusive, making it hard to verify the accuracy of topic modeling results. Thus, the assessment of topic modeling results remains an unsettled issue [12] and a generally accepted evaluation metric, such as Cronbach’s Alpha for internal consistency, is missing [13].

The present study seeks to reach a deeper understanding of the performance of topic modeling algorithms and of the interplay of procedure performance and evaluation criteria, as well as to address the two aforementioned challenges. Above all, the study is intended to make the scholarly community more aware of the main challenges associated with topic modeling and to provide improved methods for evaluating the performance of topic models. Our aim is to empower scholars to make better and more informed choices of algorithms and to enable them to assess the validity of their choices.

Our study is organized into two parts. In part one, we compare several popular topic modeling algorithms with respect to their clustering performance against a known, pre-defined representative clustering. Whereas prior research has focused on the *interpretability* and *coherence* of generated topics, this study focuses on the *accuracy of cluster assignment*. As we argue below, the assessment of the coherence and quality of calculated topic descriptors alone is not a sufficient criterion to justify the application of a specific topic modeling method, as the problem of identifying seemingly coherent but nonsensical topics, is unavoidable. However, an applied method must not only be interpretable, but also prove to be reliable and valid. In response to this requirement, we focus on determining accuracy of the algorithms that we benchmark. In part two of our study, we analyze the correspondence of existing evaluation metrics with an optimal clustering. We extensively investigate the problem of inaccuracy caused by selecting unsuitable algorithms and/or choosing inappropriate metrics.

For both parts, our study requires a representative dataset for topic modeling tasks including a known clustering. Currently, researchers typically rely on a number of different datasets for their tests, which differ greatly in characteristics and hence may not be generalizable to real-world, exploratory topic modeling tasks [11]. Additionally, they only allow the variation of cluster sizes and number of clusters to a very limited extent. As no standard test datasets with the required properties for our test set up have yet emerged, we create an automatic dataset generation procedure for this purpose ourselves. The procedure is based on exploiting the Wikipedia category scheme. With it, our findings are more generalizable to a potentially unlimited number of datasets with human-assigned labelling. We believe replication of our approach might prove useful for a wide range of social science researchers and users of text clustering algorithms to further develop their applications.

Overall, we contribute to research on computational social sciences with a comprehensive and automated comparison of topic modeling algorithms and their evaluation metrics using a large number of non-artificial datasets featuring an unbiased result specification. In this way, we are able to identify candidate algorithms and metrics yielding best clustering performance in terms of accuracy. Hence, we can give researchers guidance regarding the validity and reliability of these methods. We also make publicly available the datasets utilized and their generation procedure. By doing so, we seek to enable other scholars to fully reproduce our results and to conduct their own performance experiments adapted to their specific research needs. We also hope that our work will provide a new evaluation standard for comparing topic modeling algorithms.

Our paper is organized as follows. First, we provide background information on topic modeling algorithms and evaluation metrics used in conjunction with topic modeling. Next, we describe our experimental setup for the comparison of algorithms and evaluation metrics, and explain the procedure for generating our test datasets. Then, we present our experimental results and outline best practices on how to improve the application and usage of topic modeling. Finally, we conclude with a brief summary, a discussion relating our findings to previous work, and suggestions for future research.

## Background

In the following two sections, we first give provide a short introduction to topic modeling algorithms and then outline approaches to evaluate topic modeling results. Both sections introduce the essential terms for the subsequently presented research design and the discussion of our findings. We also revisit related works. For the interested reader, we provide a deeper understanding of modeling algorithms and assessment metrics in our S1 Appendix. This involves introducing their underlying principles in an intuitive way and explaining their inter-relationships to each other.

### Topic modeling algorithms

Clustering has been studied extensively in many scientific disciplines and a variety of different algorithms have been developed. Fundamentally, clustering is defined to be the problem of finding groups of similar objects based on a similarity measure in a given dataset without possessing any further information on the group memberships of these objects. Due to this aspect, clustering algorithms are often referred to as *unsupervised* data mining methods, in contrast to *supervised* data mining methods, such as classification or prediction. Traditional methods for clustering have generally focused on the case of *structured data* such as numerical data presented in a spreadsheet. In contrast, *unstructured data* does not have a pre-defined data model [14]. This is especially true for text data, which exhibits a number of unique properties that may render traditional clustering algorithms unsuitable. Though many algorithms can be extended to any kind of data, they typically do not work well on some for forms of text data [15], as we outline below. Hence, text data calls for specialized algorithms.

Generally speaking, text clustering algorithms that are designed to discover the themes that occur in a corpus, or collection of texts, are called topic modeling algorithms. Thus, they are part of unsupervised data mining methods and differ from classification methods of text such as sentiment analysis. Topic modeling is concerned with the discovery of latent semantic concepts or topics within a document collection [5]. Intuitively, one would expect particular words to appear more or less frequently in a document, given that it is concerned with a particular topic, and a document may comprise multiple topics in different proportions. For instance, a research article might be embedded in a specific theoretical framework using terminology related to its constructs and mechanisms. Moreover, it might apply a specific research methodology like regression analysis. Our example article might thus cover (at least) two topics, the theory and the method, and their respective terminology. Topic models capture this idea in a framework that identifies the topics and assigns topics to the documents. A document might cover more than one topic, which is indicated by a weight or loading coefficient. Therefore, topic models follow a *mixed-membership* approach and belong to the class of *soft clustering algorithms*, in contrast to *hard clustering algorithms (single-membership approaches)*, which assign every object to one cluster only [14]. Of course, soft clustering algorithms can also be used to assign each object to only one cluster. In fact, in many cases topic models are used in exactly this way, by only considering the cluster with the largest weight per object, while ignoring all other assignments. In this context, the term *topic* corresponds to the term *cluster* in data mining. The major challenge is that these clusters are not known in advance, but must be learned or ascertained automatically by analyzing a collection of documents [15]. Although topic models were first described and implemented in the context of natural language processing, they also have applications in various other fields, such as bioinformatics [16]. Topic modeling algorithms usually output lists of the most representative words that are strongly associated with each topic. The lists are called *topic descriptors* [15]. However, it is important to stress that the term “topic” does not necessarily correspond to an intuitive understanding of the term. Because the procedures capture texts based on statistical features, they sometimes produce topics that merely reflect linguistic features in the text. These topics may not be of any value to the analyst [17]. Thus, the topics themselves have to be identified by manual pruning and labeling *after* the application of the algorithm. For this reason, manual validation of the results is imperative. For a detailed discussion on the application of topic models we refer to [18, 19]. From an algorithmic perspective, topics are just patterns of word co-occurrences in a set of documents, but the exact understanding varies from procedure to procedure. Some more details about this can be found at the end of this section.

All topic model approaches to date build on the vector space model (VSM) introduced by [20] as a mathematical representation of text, and take a document-term matrix (DTM) shaped from a document collection as input. The vector space model is based on the assumption that the meaning of documents can be derived from their constituent terms. All terms occurring in the document collection define the dictionary and all vectorized documents form the document-term matrix. Several different ways of computing the vector components, also known as term weighting schemes, have been developed [21]. The simplest way of document encoding is binary: a vector element is set to one if the corresponding word is used in the document and to zero if it is not used. Instead of a binary representation, the vector elements may also be just the occurrence count of the terms contained in a document. Thus, documents can be compared by use of simple vector operations. The similarity of two documents then may be computed using the cosine similarity. In contrast to simple counts, term weighting schemes are usually used, where the weights reflect the importance of a word in a specific document of the document collection [21]. In the classic vector space model, the term-specific weights in the document vectors are products of local and global parameters [20]. The model is known as the term frequency-inverse document frequency (TF-IDF) model. Large weights are assigned to terms that are used frequently in relevant documents but that rarely appear in the whole document collection [22]. Intuitively, the inverse document frequency acts as a normalization factor to prevent distortion of the overall measure by documents of different length. In our experiments, both count-based and TF-IDF-based text representations are used. Probabilistic topic modeling algorithms require counts as inputs, while the ones based on matrix decomposition may be fed with both text representations.

The vector space model, also called bag-of-words (BOW), has the following advantages. First, it is a simple model based on linear algebra and therefore easy to understand and implement. Second, it allows for computing a continuous degree of similarity between encoded documents. This is especially important because clustering algorithms require a similarity measure to be able to form groups of similar objects. However, VSM has some limitations, too. Usually, large documents are poorly represented because they have poor similarity values. Additionally, documents with similar contexts but different term vocabularies are not matched, and synonyms and homonyms are neither recognized nor taken into account. Most importantly, this representation does not account for word order and semantic relations, and therefore renders only a very incomplete snapshot of the language used. As a result, the obtained DTM, that is, the vector representations of all documents in a collection, is usually high-dimensional, very sparse, and consists of words with meanings that are not easily discerned out of context. This often makes it difficult to grasp the semantic information it contains [15].

Topic modeling procedures overcome some of these issues by extracting the underlying themes from the collection of documents. These procedures identify low-dimensional factors in the high-dimensional data while retaining the overall semantic structure and simultaneously reducing the lexical variability. The general idea dates back to the work on latent semantic indexing (LSI) by [23] in information retrieval, and is known as latent semantic analysis (LSA) in topic modeling. These algorithms decompose of the document-term matrix using singular value decomposition (SVD) as a dimension reduction technique, performing a low-rank approximation of the document-term matrix to uncover basic semantic concepts. This is similar to principal component analysis (PCA). For the same purpose, others have applied non-negative matrix factorization (NMF) instead, a variant of SVD with additionally-imposed restrictions for the matrix decomposition to address the issue of the difficult interpretability of the approximation obtained from SVD [24–26]. [6] introduced a probabilistic variant of LSA, namely probabilistic latent semantic analysis (PLSA). Probabilistic topic models have become popular in recent years and interpret topics as a probability distribution over words, with documents being mixtures of topics [27]. One extension of the PLSA model is known as Latent Dirichlet Allocation (LDA) and is one of the most frequently used topic modeling techniques today [5]. In recent years, many different variants of these algorithms have been developed. But in this work, we will refer to the general forms of these algorithms that are not adapted to or customized for specific use cases. Table 1 gives a brief overview.

**Table 1:** Topic modeling algorithms included in the experiments of this study.

Algorithm	Type	Reference
Latent Semantic Analysis (LSA)	Matrix Decomposition	[23]
Non-Negative Matrix Factorization (NMF)	Matrix Decomposition	[25]
Probabilistic Latent Semantic Analysis (PLSA)	Probabilistic Model	[6]
Latent Dirichlet Allocation (LDA)	Probabilistic Model	[5]

### Topic modeling evaluation metrics

As described above, the application of topic models comes with a number of design decisions including what algorithm and inference method to use, what model parametrization to use, and how many topics to extract. To be able to narrow down the set of possible design options, a single quality criterion, accuracy, is required. Of course, other criteria such as computational complexity or speed of calculation are also relevant. However, we argue that accuracy is most important to find the clustering that best mirrors reality and, relative to accuracy, that speed of calculation, especially for academic purposes, might be of less importance. Due to the stochastic nature of some inference processes, the quality of the results can vary greatly, even for different runs on the same dataset. Therefore, researchers need to be able to identify high-quality models from several runs [28].

The choice of a clustering evaluation metric depends on the application and available information. There are two ways to evaluate topic modeling results. One option is to use measures describing the internal properties of a clustering result. In topic modeling, this may be applied to the topic-document assignments or the topic descriptors. In data mining, this is known as *internal cluster validity* [14]. Internal cluster validity measures consider structural aspects of clusters such as their degree of separation and do not rely on any additional information related to the input data. This gives an idea of quality that may not correspond to the perception of humans. However, in many cases, it is the only viable option, because there is no referenceable knowledge structure to which the clustering of texts can be compared [17]. Another option is to compare the clustering results against a source of external knowledge, also called *groundtruth*, commonly in the form of a known classification. Usually, this refers to a manually-obtained labelling of clusters. Obviously, such a manual labelling is based on human perceptions and depends on the expertise of the raters. This option is known as *external cluster validity* [14]. In an exploratory scenario, it is usually not available, as no groundtruth is present. However, in the benchmarking scenario of this paper, we will rely on external criteria. Obviously, only when groundtruth is available is it possible to derive reliable statements concerning the accuracy of a topic modeling procedure. A social scientist may also relate the quality metrics presented here to the criteria applied in qualitative research, as it is a comparable exploratory scenario [29]. In the context of this study, the validity of a metric is understood as accuracy–*the closeness of a measurement to an external value*. Reliability comes in the form of test-retest reliability and is relevant for clustering algorithms, especially the non-deterministic ones.

The most commonly used internal evaluation measure is the perplexity of a held-out dataset with respect to an inferred model [5, 12]. This measures a model’s ability to generalize and predict newly-presented documents, and is based on the model’s likelihood [30]. Propose a Bayesian method for measuring how well a topic model fits a corpus, based on posterior predictive checking. It shows where the model does and does not fit the observations [31]. Suggest a topic evaluation method to identify and distinguish junk topics from meaningful ones. The basic idea consists of measuring the distance between a model’s topic distribution and distributions that take the form of a uniform distribution over the dictionary. Other researchers approach the problem from the direction of model selection, in most cases to find the optimal number of clusters to extract [32]. Propose a method to select the best topic model based on topic density, a measure of the average cosine-distances between topic descriptors [33]. Recommend comparing the singular value distribution of the topic-term matrix with the row *L*_1_ norm of the document-topic matrix to identify the right number of topics. They compare their new metric with the aforementioned measure of perplexity in their experiments. To estimate the correct number of latent concepts of a topic model [34], propose a method using Jensen-Shannon divergence, which is a symmetrized version of Kullback-Leibler divergence, on the topic-term distributions, with the aim of maximizing the information divergence between all pairs of obtained topics.

Another family of measures is word-based topic coherence measures, aiming at the semantic interpretability of the uncovered topics. These measures are motivated by the work of [17], who frame the evaluation of topic coherence as a word intrusion task. In this task, human judges were asked to identify intruder words inserted randomly into a set of words characterizing a particular topic. Assuming that finding the intruder word in coherent word sets would be easier than in incoherent word sets, the researchers found that the human notion of coherent topics does not correlate well with perplexity. Hence, they advocate for new semantic measures for topic model quality instead of the formerly described techniques, which are dependent on statistical features. In this context, coherence refers to sense relations between single units of a text. Due to these relations, a text appears to be logically and semantically consistent for the reader. Texts with high coherence consist of text units allow for the immediate recognition of semantic relations. As topic modeling algorithms describe the found topics with lists of words, coherence refers to the semantic connectedness of these words [35]. Propose a method to fully automate the original word intrusion task. To calculate word-based topic coherence measures automatically, most variants average pairwise semantic similarities of the most likely words of a topic [36]. Give a systematic evaluation of the numerous word-based topic coherence measures proposed in the literature and introduce a generic framework for coherence computation. By searching through the space of measures defined in this manner [36], derive several novel topic coherence measures with superior performance compared to the previous ones. However, the first automatically-calculated word-based topic coherence (*C*_*UCI*_) measure was publicized by [37]. It is calculated using WordNet-based and Wikipedia-based similarity metrics and pointwise mutual information (PMI). Instead of PMI [38], employ the pairwise log-conditional probability of words, estimated based on document frequencies of the original corpus (*C*_*UMASS*_). The metric of [39] evaluates a topic’s word set with regard to corresponding WordNet concepts [40]. Introduce topic coherence based on context vectors for every word in a topic’s word set, based on word co-occurrence counts of words around the word in the word set. Additionally, they show that the *C*_*UCI*_ coherence performs better if the PMI is replaced by normalized pointwise mutual information (NPMI). Another approach by [41], including external knowledge in the form of word embeddings, calculates the average of the cosine distances of word embeddings for all words in a topic’s word set [42]. An overview of embedding techniques for topic coherence computation can be found in the work of [43]. [44] present variants of word-embedding-based coherence metrics and test them on Twitter data. Four variants of word-embedding-based coherence measures using different distance metrics are evaluated by [45], who compares them to prior coherence methods [46]. Discuss partitioning the word sets into subsets and calculating the measures over the subset pairs instead of calculating pairwise word similarities. Drawing on this idea [47], propose a coherence measure based on grouping the topic words into coherent clusters. This, too, is achieved using external knowledge in the form of word embeddings. Here, the coherence score of a topic is defined as the size of a principal cluster representing the main concept of a topic.

[48] introduce document-based coherence measures. The coherence scores are calculated based on the most likely documents rather than the most likely words of a topic. To this end, first, a number of top documents are obtained from a resulting topic. Then, the documents are vectorized using one of the following methods: bag-of-words using word counts, TF-IDF vectorization, or aggregating the word embeddings of all document terms. Finally, the similarity of the vectorized documents is obtained using distance-based, density-based, or graph-based methods. By varying and testing the available options for calculating the coherence scores [48], find a number of best performing document-based coherence scores in the context of a classification task using two datasets. Finally, they demonstrate that there may be some benefit in combining word- and document-based coherence measures.

Not a quality criterion per se, and therefore not included in our analyses, but nevertheless another interesting internal measure, is stability [49]. Define stability as the mean document correlation of the document-topic matrix of best-matching topic pairs over all topics obtained from two algorithm runs of a topic model. The stability of clustering procedures is a desired property, especially for algorithms which include stochastic elements. Similar experiments were conducted by [50]. Typically, the problem of instability is not considered with necessary care. Computed results are often treated as final, although the results can change significantly if the initialization process is changed [51]. For this reason, we pay special attention to this issue during the experiments. Building on this prior work, Table 2 summarizes the metrics used in our study.

**Table 2:** Internal metrics for topic modeling result assessment included in the experiments of this study.

Metric	Basis	Reference
Perplexity	Likelihood of a Held-out Dataset	[12]
Topic Distance	Topic Cosine-Distance	[32]
Symmetric KL-Divergence	Topic KL-Divergence	[33]
*C*_*UCI*_	Word-based Topic Coherence	[37]
*C*_*UMASS*_	Word-based Topic Coherence	[38]
*C*_*NPMI*_	Topic KL-Divergence	[40]
Topic Divergence	Word-based Topic Coherence	[34]
*C*_*V*_	Word-based Topic Coherence	[36]
*C*_*W*2*V*_	Word-based Topic Coherence	[41]

### Study design

Scholars currently rely on many different datasets when it comes to testing topic modeling algorithms. However, their appropriateness for baseline tests appears questionable, as the datasets vary considerably in terms of corpus size, document length, topic size, and the amount of noise. As some of these characteristics prove challenging for various topic modeling procedures, they may not be representative choices for real-world data. From our point of view, analyses based on these datasets should only be considered relevant if one’s own data is relatively similar to the datasets used therein. Thus, in our study, we aim to select our test datasets as close to reality as possible. However, this first requires us to define the scope of our datasets.

In this study, we take topic modeling as a method for the exploratory thematic analysis of large document collections and thus define our experimental scenario with this in mind. To appropriately select test data, we leverage the inherent properties of topic models, including their advantages and disadvantages in various usage scenarios. Typically, bag-of-words-based methods work poorly on tasks that require precise semantic meaning, because they do not account for word order [21]. However, for large collections of documents, the assumption of exchangeability provides a theoretical foundation for the applicability of such methods [5]. This point harmonizes with the findings of [52]. In their systematic review of the inferential performance of LDA topic models, they identified a number of limiting factors of the input data that influence the effectiveness of the procedure: document length, number of documents, and the prior distribution hyperparameters of LDA. Considering the results performed on two simulated and three real datasets, they make five key recommendations. First, they argue that a sufficient number of documents is the most important factor to ensure accurate inference. Second, they state that the documents must neither be too short nor too long. Third, they find that the statistical inference of a large number of topics tends to be inefficient. Fourth, they find that algorithm performance is affected by the separability of the underlying concepts in the Euclidean space. And fifth, they advocate for a proper setting of hyperparameters.

Following the five key recommendations of [52], and striving to use a representative, real-world scenario, we chose to use the English Wikipedia as a data source for the following reasons. First and foremost, Wikipedia is a tremendously large text collection, making it possible to draw a effectively unbounded number of subsets from it. It has enough text to generate collections with document sizes of almost any length. Its texts have been reviewed by many authors and therefore tend to be syntactically and semantically consistent. It is free from application- or scenario-dependent noise such as emoticons and symbols. The information is publicly available and can be processed using open source tools to remove any unwanted artifacts such as Wikicode markup elements. The straightforward opportunity of obtaining texts makes it a valuable data source for the evaluation of text clustering algorithms. In addition, the extracted articles contain the respective topics to the extent that a statistical procedure can easily recognize them. It is crucial to emphasize that this is not necessarily the case for many other test datasets. Over the years, Wikipedia has become a *de facto* standard as a data source for data miners and linguists. Its properties are well understood within those communities. All in all, Wikipedia is an ideal choice for this kind of usage scenario. Additionally, and perhaps more importantly for our purposes, Wikipedia provides a rich category scheme serving as a community-generated human baseline that can serve as a meaningful reference point for the evaluation of the results. Because so many people work together on Wikipedia’s construction, it is reasonable to assume that the available categorization scheme is considered to be correct and accepted by a large number of people. While others have used Wikipedia texts to evaluate text mining procedures, we are, to the best of our knowledge, among the first to use their category scheme for this purpose.

To create our test datasets, we downloaded the entire English language Wikipedia site, processed the XML files we obtained to extract all article texts and the category scheme, and filtered out categories containing merely of lists of articles with no common theme as well as all categories used for administrative purposes. Detailed information on the extraction procedure as well as the conditions for the (re-)use of the datasets can be found in the S1 Appendix. Then we identified all categories containing at least 50 articles, each with a minimum length of 1000 characters and at least 500 words to make sure that the text contains enough content to be able to derive a theme from it. For each series of tests, we sampled categories from the obtained set of candidate categories. This design decision takes into account the aforementioned framework conditions of [52] for a sensible use of topic modeling. For each sampled category, we extracted 50 articles from categories meeting the above criteria. In the following, those 50 extracted articles from one Wikipedia category form one topic. In our understanding, a *series of tests* is a group of test runs beginning its first run on a dataset comprised of two topics, then continuing with a test run on a dataset with an additional topic, and so on. In this way, we kept adding topics, i.e. a set of 50 articles from one Wikipedia category, until we reached 75 categories or 3750 articles, in step sizes of one, two, or five. All algorithms were executed 25 times on each dataset of each test series. By repeating the experiments on a number of test series, we averaged out the influence of individual effects of the themes.

We evaluated the quality of topic models using frequently-employed variants of the two representative families, non-probabilistic (LSA and NMF) and probabilistic methods (PLSA and LDA). We used general variants of the procedures not adapted to specific use cases. For LSA, we used a general implementation of truncated singular value decomposition (SVD). The implementation of non-negative matrix factorization was based on [53, 54]. PLSA is mathematically equivalent to NMF, with an *L*_1_ ratio of 0.5 [55]. Accordingly, it is computed using the same implementation. Because a full inference of LDA remains infeasible, approximation methods are required. Originally presented with a variational Bayes approximation method, several additional variants of LDA have been proposed in recent years. The two most popular ones to be found in publicly available toolkits are the online variational Bayes method of [56] and a fast implementation sampling based-method rooting in the work of [57, 58].

In our experiments, we employed algorithm implementations widely used in research. In the case of LDA, these come from *Mallet* [59], *Gensim* [60] and *Scikit-Learn* [61]. *Mallet* provides a sampling based-implementation of LDA and *Gensim* and *Scikit-Learn* contribute an online and a batch variant of Variational Bayes LDA to the test environment. *Scikit-Learn* also adds implementations of LSA, PLSA, and NMF. For comparison purposes, we also included a popular variant of the *k*-means algorithm in our experiments. From a practical point of view, k-means is one of the fastest clustering algorithms available, but it may not be an appropriate choice for clustering large amounts of text, e.g. due to the high dimensionality of the data.

A critical step in text analytics is the text preprocessing. This procedure, interwoven with text vectorization, involves a series of actions to clean and normalize text with the goals of removing potential noise and maximizing the underlying signal. Typical steps include the conversion of the texts to lowercase, the removal of stopwords, and the stemming of the terms to some kind of root word form [62]. To create comparable conditions for all tested methods, equally preprocessed texts were fed to all tested algorithms. To ensure the appropriateness of our preprocessing, we compared a number of combinations of typical preprocessing steps in terms of their influence on the results. Our preprocessing pipeline determined in this way began with the lowercase conversion of all input texts, except for terms written all in capital letters and with a length of two or more characters. The purpose of the exception is to prevent confusion between abbreviations and regular words. Then, we identified all multi-word expressions using a list of noun phrases comprising up to six words extracted from all Wikipedia article titles. These noun phrases were regarded as multi-word expressions in the following processing steps. Then, we filtered out all words that were not nouns, proper nouns, or multi-word expressions. This ensures the removal of most infrequently-used words, but with the advantage of not relying on some kind of stopword list. After that, the remaining terms were lemmatized, including multi-word expressions, but excluding words in all-capital letters. Up to this point, the preprocessing was chosen to maximize the quality of the results, just as an analyst would do. To further save computation time, the vocabulary was stripped from all terms occurring in less than 5% or more than 95% of the documents. Carrying out extensive pretests on a subset of the data, we ensured that the optimizations beyond the standard level did not affect the result ranking of the top-performing procedures. The preprocessing chosen creates equal conditions for all algorithms, including algorithms that do not perform well with large vocabulary sizes. However, in real-world use, it may be considered too aggressive. We have added some remarks on the influence of the preprocessing at the appropriate place in the results section. After the preprocessing, we used TF-IDF weighting to vectorize the input texts for all matrix factorization-based methods as well as k-means. All probabilistic procedures were fed with an unweighted bag-of-words token stream.

For clustering evaluation, we calculated the external metrics *F*_1_ in two variants, as well as the adjusted RAND index (ARI) and the adjusted (or normalized) mutual information (AMI) [14, 63]. Although one external clustering metric is considered sufficient, both are reported for comparison purposes with other studies. For mixed membership algorithms, all employed external metrics consider only single memberships, i.e., the highest assigned cluster weight of the object. Wikipedia provides a large amount of textual data and a rich category schema, however, there were neither enough articles with multiple assigned categories nor information about the weight of a single category. Therefore, in this study, we measure and compare the accuracy with which topic modeling algorithms identify the dominant topic in each document relative to the human baseline of the existing Wikipedia categorization of each document. In the following section, the results on the external metric ARI are discussed; all other computed metrics are available (see S1 Appendix). The internal clustering metrics encompass perplexity, the measure of topic density [32], topic divergence *D*_*KL*_ [33], topic divergence *D*_*JS*_ [34], and the following per-word topic coherence measures: *C*_*UCI*_, *C*_*NPMI*_, *C*_*UMASS*_, *C*_*V*_ and *C*_*W*2*V*_ [46]. All these measures are used in various academic publications and many of them are available in free software packages [60, 61, 64].

In summary, our experiments comprise roughly 75,000 single test runs (25 repetitions of 9 algorithmic runs for each of the 21 tests of 5 test series plus 25 repetitions of approximately 25 extra runs per algorithm, supplied with various false numbers of clusters *k*, to assess the metric’s capabilities to identify the correct value for *k* evaluated with 13 metrics; the experiments took more than 1000 hours of computing time on an Intel i7 CPU.

## Results

In this section, we present the results of our experiments in two parts. The first section presents the comparison of topic modeling algorithms. The second part includes the results of our search for a suitable internal evaluation metric.

### Comparison of topic modeling algorithms

In this first part of our study’s results, we compare the performance of the aforementioned algorithms using external validity measures against the Wikipedia categorization schema as a baseline. The performance is determined and compared solely on the basis of the largest cluster membership per object, not on the basis of the calculated topic descriptors. The algorithm parameter *k* is preset and denotes the correct number of clusters. The performance is evaluated using the Adjusted RAND Index (ARI), measuring the similarity of the baseline and the calculated clusterings with chance normalization and ignoring permutations (larger values are better). It is particularly suitable for clusterings consisting of large and equally-sized clusters, as it is the case here [63]. Detailed information on procedure-specific settings as well as the dataset characteristics are listed in the S1 Appendix. Here, we emphasize that the presented values are not to be considered as an absolute measure, but that the relative order of the obtained results is most important. With our setup we attempted to identify the average behavior of the methods over a very large set of experiments that are as realistic as possible. However, each application of topic models necessitates tailoring of preprocessing steps and parameterization choices to the data at hand, which may cause the procedures to behave differently.

**Fig 1:**
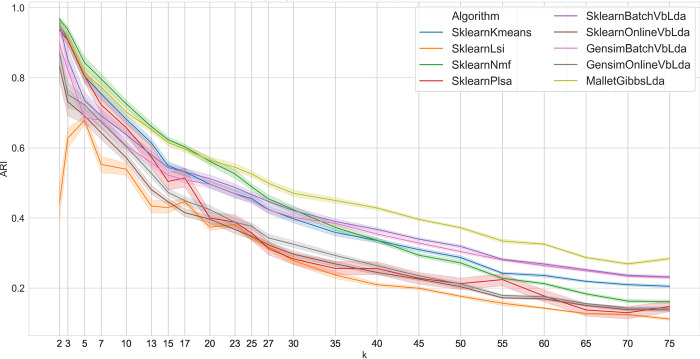
Average algorithm performance on all dataset series.

The relationship between performance and the number of clusters *k* is illustrated in Fig 1. We discuss our findings on the basis of averages over all test series to show the algorithms’ capabilities and to smooth out dataset-specific qualities. The graph shows some irregularities due to the use of non-artificial data, but they do not invalidate the outcome as a whole. For a small number of clusters *k* with *k<20*, non-negative matrix factorization (NMF) outperforms all other algorithms, sometimes alternating with sampling-based LDA. Although technically similar to LSA and PLSA, this algorithm is almost never included in performance comparisons of topic modeling procedures. Only with larger values of *k* (*k>20*), the relative strength of the other procedures become apparent. Then, the sampling-based LDA approach dominates all other algorithms. LSA yields much worse results in comparison, especially with increasing *k*. The two batch implementations of variational Bayes LDA (VB-LDA) consistently give better results than do the online variants. They show similar performance, especially for larger *k*, and surpass NMF, whose performance drops significantly for larger *k*. As already stated, the effective accuracy of all algorithms would actually be higher with a preprocessing tailored to the algorithm. The lead of the best performing algorithms LDA and NMF would be even greater. However, all the algorithms slowly but steadily lose predictive power as the number of clusters increases. With increasing numbers of clusters *k*, the difficulty of telling them apart increases as well. The shading around the lines in the figure give a hint of the result scattering of the 25 runs on each dataset, especially for LSA. However, result scattering also appears to be relevant for values of *k* smaller than 15 for all variational Bayes LDA variants. This may seem unexpected in the first place, but the implementations of the originally-deterministic variant VB-LDA also use sampling in parts to speed up the computation. This fact is often ignored in practice.

**Fig 2:**
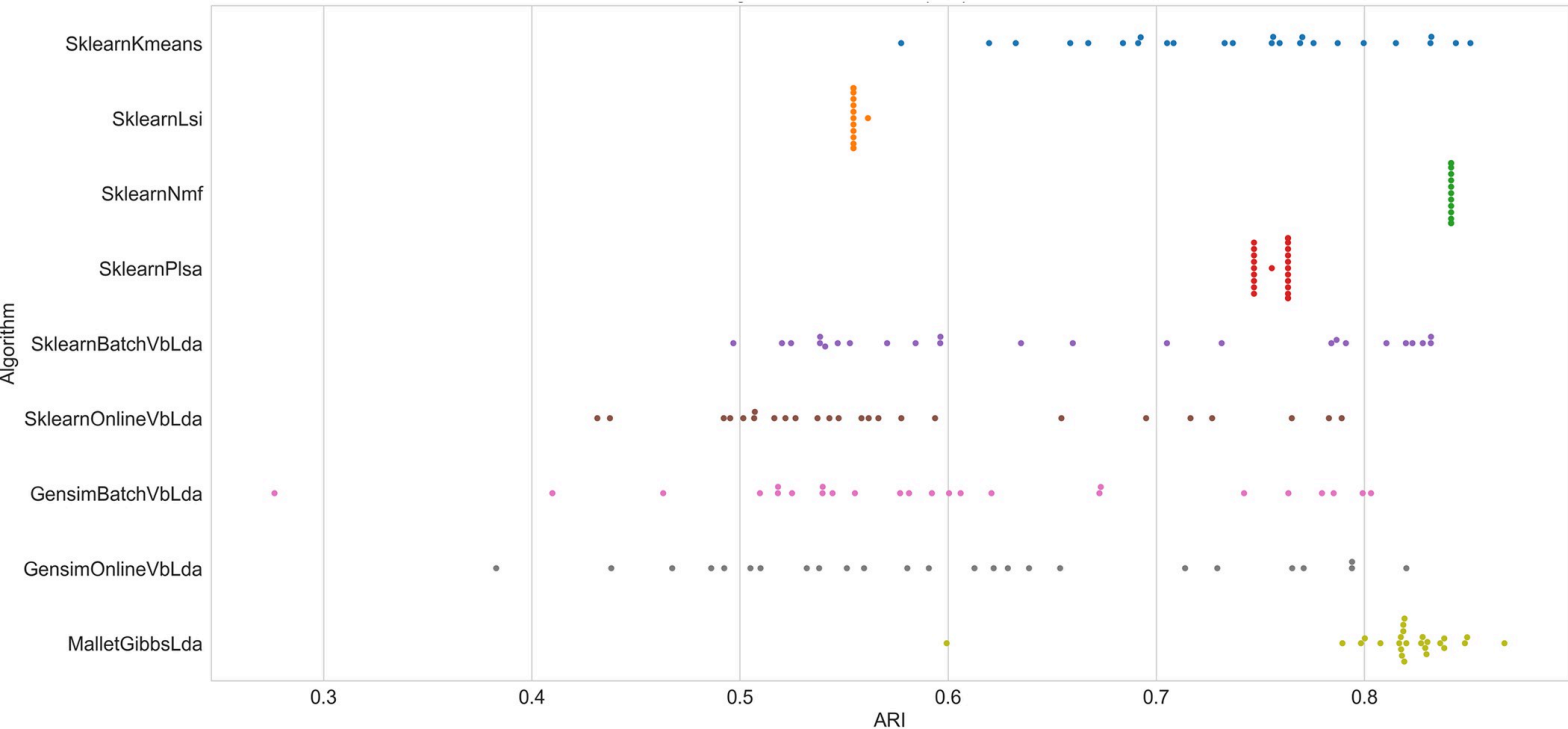
Algorithm performance (ARI) on the WikiA5 dataset.

**Fig 3:**
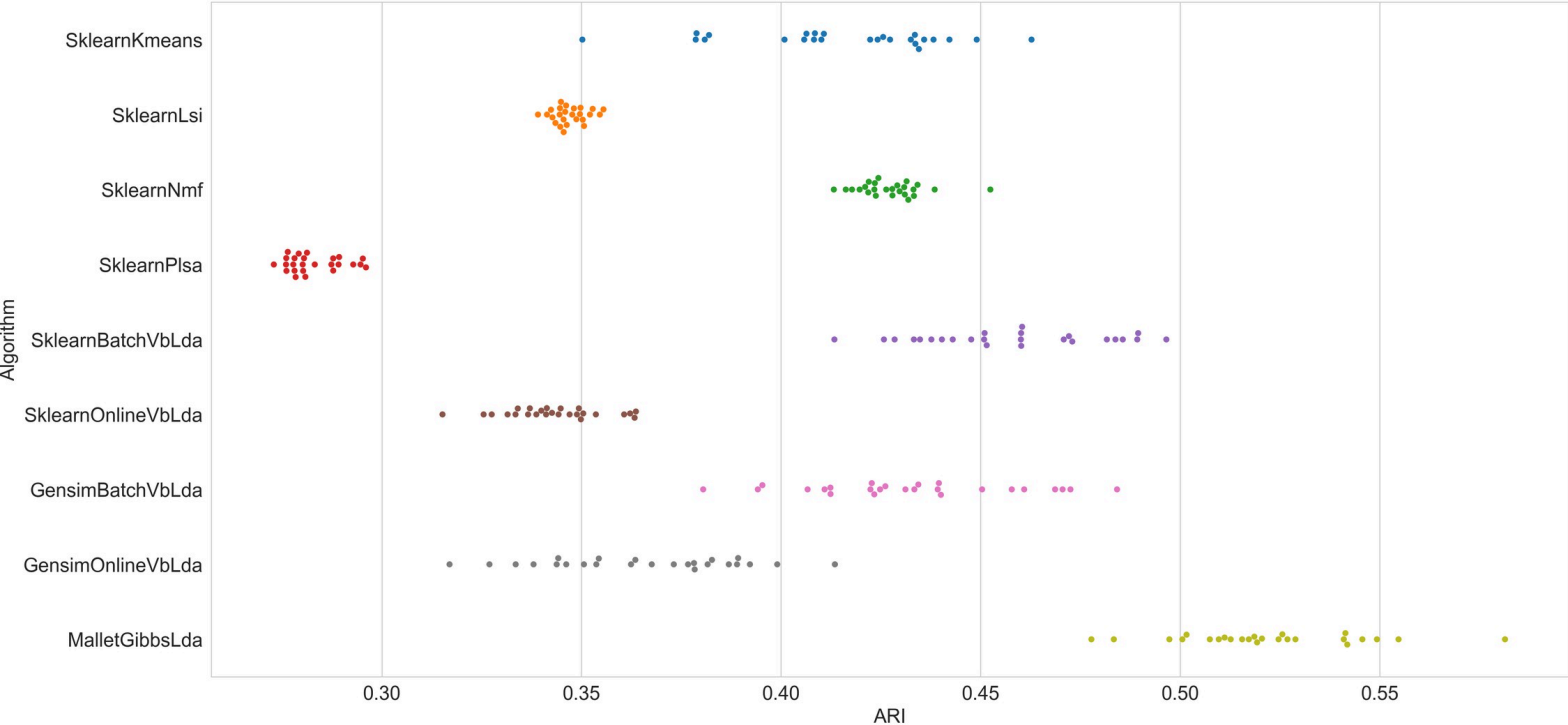
Algorithm performance (ARI) on the WikiA25 dataset.

To shed further light on this aspect, Figs 2 and 3 show the result scattering of all algorithms on datasets with *k = 5* and *k = 25* on one of the test series, each dot represents the 25 runs for each algorithm. Obviously, the results of the 25 runs on the Wikipedia dataset with five clusters scatter heaviest with the variational Bayes variants of LDA as well as k-means, whereas all the other algorithms perform almost equally on each run. Regarding the result scattering of VB-LDA algorithms, a similar picture emerges on the dataset with 25 clusters in Fig 3. As expected, all algorithms perform less accurately on this dataset, as it poses a more challenging task; and algorithms whose performance was almost constant before now show some variation in their results. Sampling-based LDA is also impacted, but shows a smaller increase in result dispersion. As the number of clusters *k* rises, the spread of results decreases, albeit at a lower overall level.

**Fig 4:**
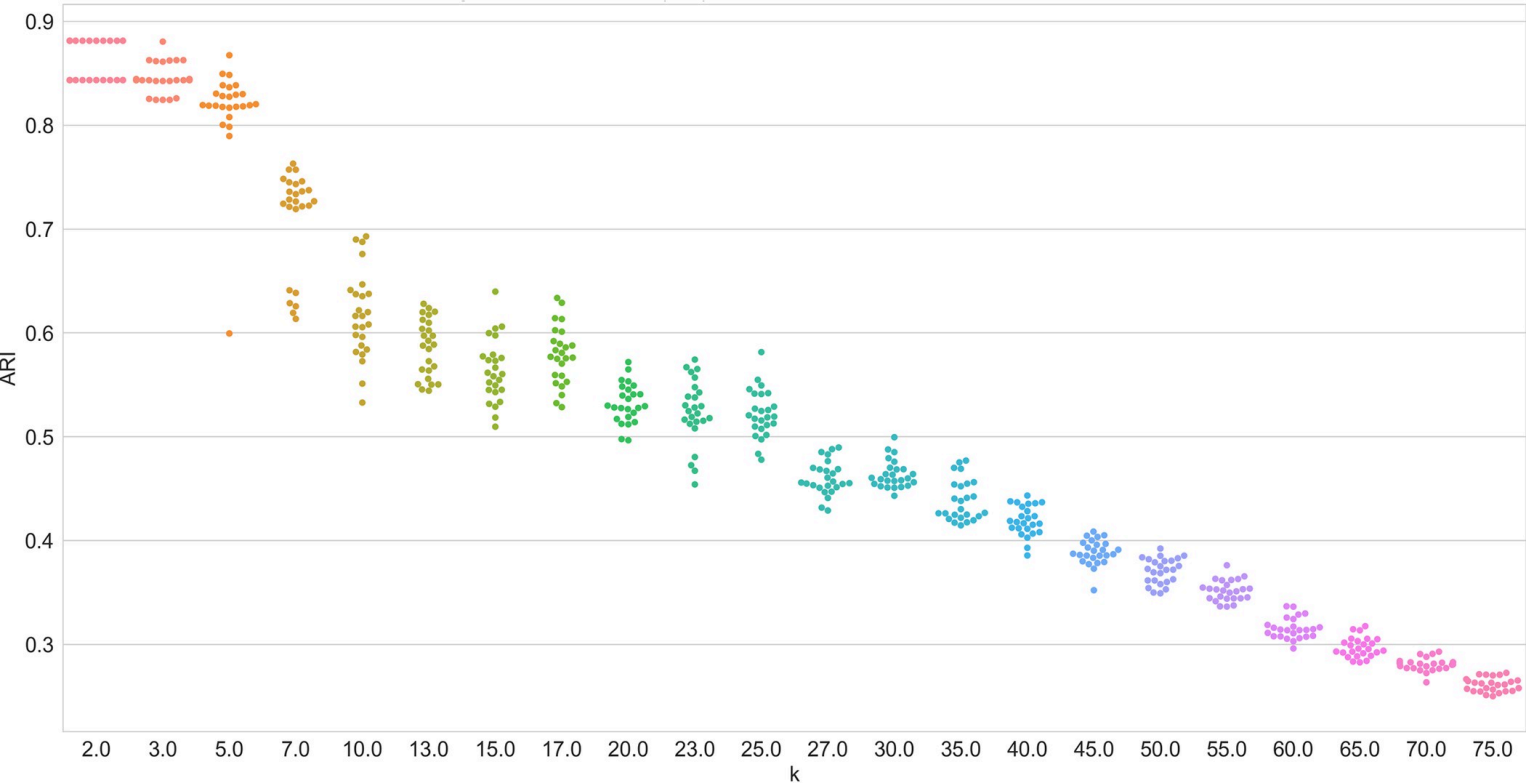
Algorithm performance (ARI) of sampling-based LDA on the WikiA dataset series.

**Fig 5:**
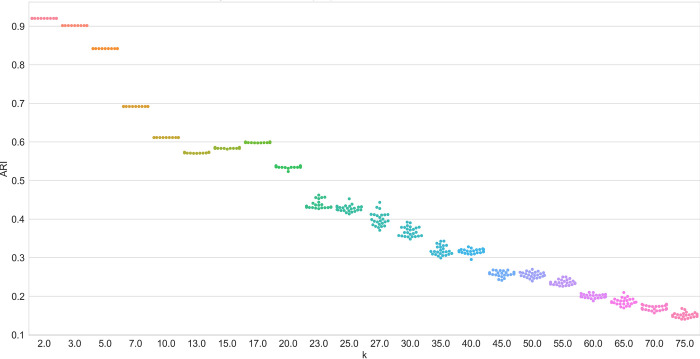
Algorithm performance (ARI) of NMF on the WikiA dataset series.

In practice, the result scattering characteristics of the algorithms are of utmost importance because result scattering determines the likelihood of receiving an optimal result and hence the necessity of repeated computations. A social scientist may revisit the concept of reliability, i.e. referring to achieving similar results under consistent conditions. In this context, a low result dispersion signifies high reliability. Figs 4 and 5 show the result dispersion for selected datasets. They show the different convergence behaviors of the clustering algorithms. While NMF does not exhibit result scattering up to a count of approximately 20 clusters, all the LDA variants clearly show result dispersion. Not depicted here, the online variants of VB-LDA show massive scattering for small *k*, rendering a single run almost useless. The illustration shows that the results of the sampling-based variant of LDA also exhibit result scattering, but to some lower degree. Remarkably, it clearly reveals the search for local optima on small *k*, recognizable by the almost discrete steps in the swarm plot (Fig 4).

To summarize the results of the first part of our study, for small *k* (roughly *k<20*), NMF outperforms all other tested clustering procedures. It performs better both in terms of clustering accuracy and result scattering. Hence, NMF does not require a separate testing procedure to determine the optimal run with the same parameter settings. Additionally, the number of clusters *k* is the only parameter this procedure demands, and it is much faster than all the variants of LDA. For small *k*, even the k-means algorithm performs well, but scatters due to its stochastic initialization procedure. However, this might result from non-overlapping cluster allocations inherent in our test data. In a scenario that requires the assignment of documents to several topics, *k*-means as a partitioning algorithm would not be the right choice. The sampling-based variant of LDA performs well on datasets with larger k (roughly *k>20*), but the results scatter, and a measure to determine an optimal run is required. Overall, it is quite surprising that in the context of topic modeling, especially for small *k*, the performance benefits of using NMF do not appear to be fully appreciated by the research community. As this study focuses on more than cluster assignment correctness, the results show that scholars should clearly prefer NMF for expected small *k* and sampling-based LDA for larger *k*. The variational Bayes variants of LDA are clearly outperformed by NMF. Furthermore, as they also use sampling techniques during the process, they do not have the advantage of being deterministic. Hence, these variants of LDA also necessitate the selection of an optimal run out of several runs. An argument in their favor is that they are much faster than sampling-based LDA. Additionally, the models of the online variants of variational Bayes LDA can be updated any time and do not require the recalculation of the entire model. In an exploratory research setting, these two criteria may be of low significance. In a production application environment with a constant feed of data, that may not be the case. Users should carefully consider these factors when deciding which algorithm to apply to their data.

### Comparison of topic modeling result assessment metrics

In the second part of our study, we examine the relation between the result assessment metrics and optimal clustering results. As in the previous part of the study, the performance is determined and compared solely on the basis of the cluster memberships using the Adjusted RAND Index (ARI). Again, the parameter *k* is preset and denotes the correct number of clusters. The analysis includes all previously-presented internal cluster validity measures; however, only a small excerpt of the results is presented here.

In general, internal quality criteria are often applied for two reasons. First, the number of clusters *k* is known and the best run of a number of runs of a non-deterministic algorithm must be identified. Here, usually, a domain expert has determined or estimated *k* beforehand. Second, the number of clusters *k* in the dataset is not known to the user, but is determined automatically. If a non-deterministic algorithm is employed, the best run of a number of runs must be determined in a second step. Of course, internal quality criteria are also used for their intended purpose, such as measuring a particular statistical property or the coherence of the generated topic descriptor. However, they are regularly employed as a proxy for determining model parameters such as the optimal number of clusters.

An intuitive approach may be the comparison of correlations of internal metrics with an external criterion in order to find the most accurate internal one. In a similar way, coherence measures have been related to human ratings or other internal metrics [17, 35, 65–67]. Accordingly, we begin with a Pearson correlation, including all metrics computed on all algorithms. The strongest correlations with the external validity measure ARI show *C*_*V*_ and *C*_*W*2*V*_. At first sight, this suggests that suitable internal clustering metrics exist. However, we contend that this approach is misguided, because it does not take into account that the characteristics of the various algorithms may affect the metrics differently, or that the datasets with an increasing number of *k* may pose a very different task for the algorithms. More precisely, the correlation for each single dataset (here: each test of each series) may differ in correlation from all other datasets or the aggregation of all datasets. Hence, we take a closer look at the individual results of the datasets for the various clustering algorithms; specifically, at the correlation for each dataset and each *k* individually. In this section, we focus on the two best-performing algorithms derived from the first part of our study, non-negative matrix factorization (NMF) and sampling-based LDA.

**Fig 6:**
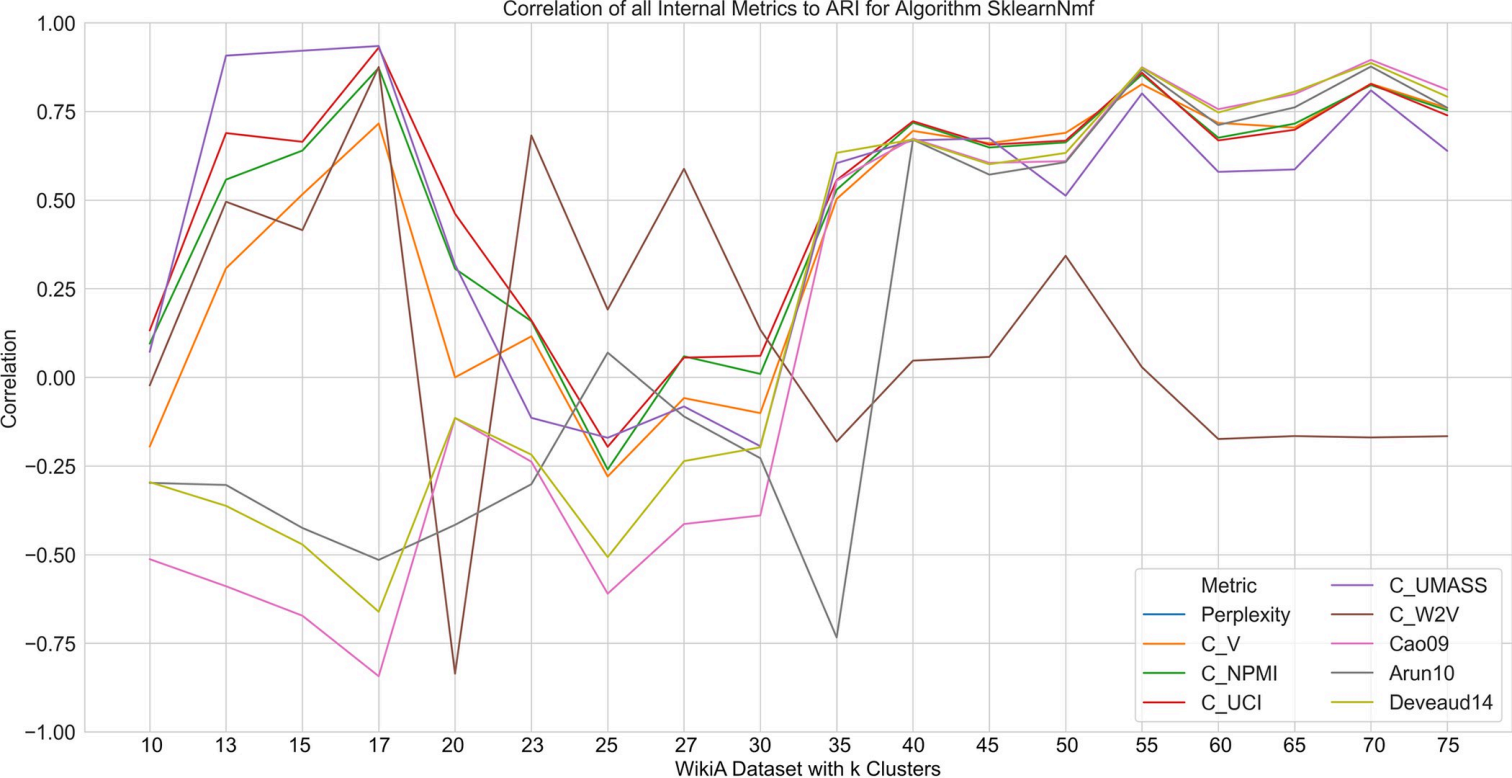
Correlation of all internal metrics to ARI for NMF on the WikiA dataset series.

**Fig 7:**
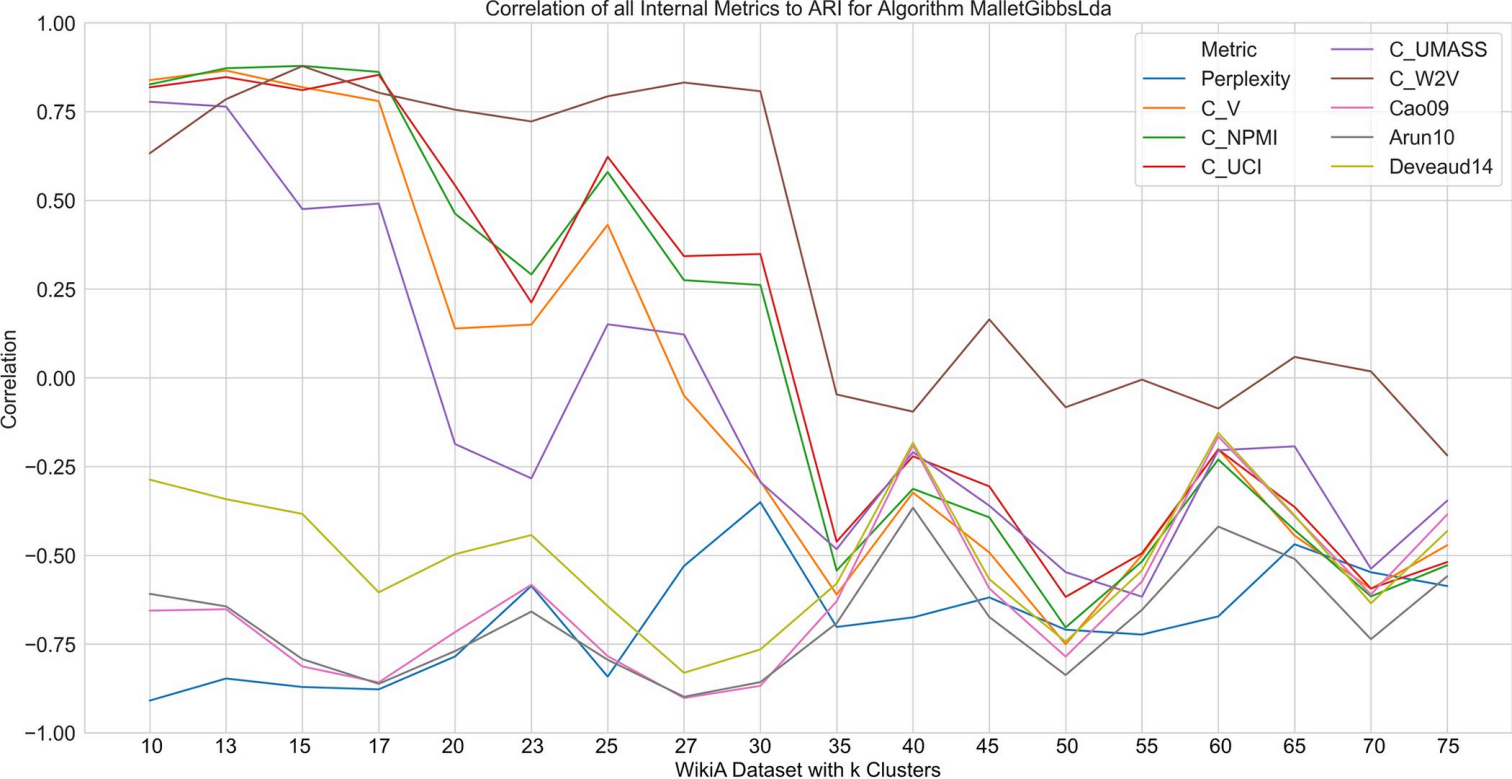
Correlation of all internal metrics to ARI of sampling-based LDA on the WikiA dataset series.

Fig 6 shows the correlation of the various internal validity metrics with the external validity metric ARI as a function of the number of clusters *k* on one of the dataset series of NMF. The correlations do not follow a clear pattern: they do not remain constant and they also do not rise or fall linearly with increasing *k*. This lack of correlation renders most internal validity measures practically useless for determining an optimal number of clusters. Only for certain ranges of *k* do some internal metrics show a relationship with the external validity metric ARI. This is the case for all coherence measures except for *C*_*W*2*V*_, which shows no pattern at all. However, since such a range is commonly not known in advance, this is of little help. Accordingly, for NMF, no internal metric for determining *k* can be recommended. For sampling-based LDA, a similar picture emerges (Fig 7). The correlations do not fluctuate heavily and indicate an approximation towards 0 with increasing *k*. The correlation of perplexity remains high and, compared to all other measures, most stable for the majority of *k*. Therefore, perplexity may be the most promising candidate for sampling-based LDA to determine *k*. In both figures, the lines converge with increasing *k*. This is to be expected, since with increasing *k* all algorithms tend to perform worse.

**Fig 8:**
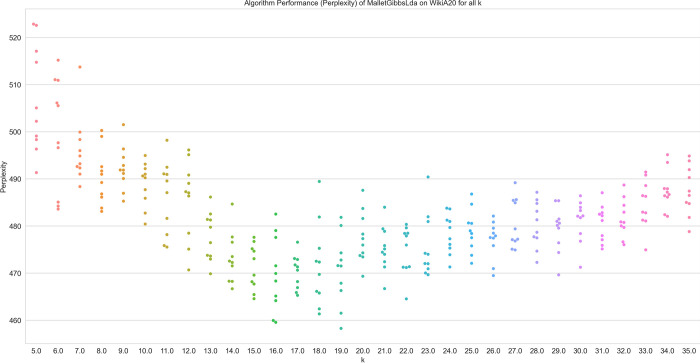
Algorithm performance (perplexity) of sampling-based LDA on the Wiki20 dataset.

However, the question remains as to how accurately the *k* can be determined. As shown above, perplexity shows relatively constant correlation with ARI for sampling-based LDA and hence might be a good candidate. Fig 8 shows the relationship of ARI and perplexity for a dataset with *k = 20* more clearly. In particular, the graph suggests how accurately the correct number of clusters can be determined at all. It becomes apparent that even in datasets with only a few clusters, for which the problem to be solved by the algorithm represents only a relatively low hurdle, the exact determination of *k* poses a real challenge. Ideally, the graph should show a clear inverted U-shape with its minimum at *k = 20* in order to allow the exact determination of the correct number for *k*. A closer look reveals that it allows for a good approximation to the correct number of *k* on this dataset, an exact determination of *k* seems unlikely. The datasets with larger *k* look roughly the same, but the minimum becomes somewhat less obvious. Thus, an exact determination of k using internal metrics appears to be improbable. Users can therefore only expect to be able to determine the approximate number of topics contained in a dataset. For all algorithms other than LDA, none of the metrics allow the determination of the correct number of *k* for any dataset.

In summary, the results from the second part of our study show that none of the employed internal measure for topic modeling tasks allows us to reliably estimate the accuracy of a result. Only for sampling-based LDA, perplexity may be selected. The experiments show that the performance of a metric is heavily dependent on the chosen algorithm and the data. For this reason, users of topic modeling should always run tests on their data, not only for the choice of an appropriate algorithm, but also for the choice of an appropriate evaluation metric. In a scenario where the accuracy of a clustering is not the primary concern, the strategy may be different.

## Discussion

In this study, we introduced the landscape of topic modeling algorithms and evaluation measures and defined the parameters for a reasonable field of application in an exploratory scenario. In a comprehensive series of experiments, we compared the performance of several topic modeling algorithms in terms of accuracy. In that sense, we measured the accuracy with which distinct topic modeling algorithms identify the dominant topic in each document relative to the human baseline of the existing Wikipedia categorization of each document. We then assessed the correlation of various internal metrics with an external evaluation measure and closely examined the validity of commonly-employed result assessment metrics. To facilitate our experimentation, we exploited the Wikipedia category scheme to automatically generate a large number of realistic datasets for the testing procedure using the category scheme as a baseline.

Our findings indicate that sampling-based LDA and NMF perform best in our experimental setup. While NMF is best suited for datasets containing presumably a presumably small number of clusters with roughly *k<20*, sampling-based LDA is best suited for datasets containing a larger number of *k*. Within this scope, NMF shows almost no result dispersion, making it unnecessary to repeat the procedure to identify a best run. In contrast, sampling-based LDA has considerable result dispersion, and hence its application requires repeated runs on the same dataset and an evaluation criterion to identify an optimal run. Our setup allowed us to assess clustering performance against an external criterion. However, as this is usually not available, researchers must resort to internal quality criteria. For sampling-based LDA, perplexity turned out to be the most promising candidate if accuracy, or correctness in the sense of the scenario presented here, is the goal of the analysis. For NMF as well as all other tested algorithms, none of the analyzed internal metrics can be recommended. Since we do not offer a practical remedy for this challenge in this study, we would like to call upon the scholarly community to build upon our initial results and intensify their efforts in finding a valid internal metric for topic modeling accuracy. This is accompanied by an appeal to users of the method not to rely solely on the metrics available to date but instead to manually validate the results in the spirit of good qualitative research. By providing our data sets, we encourage fellow researchers to replicate and extend our research in new directions and further investigate potential metrics.

### Comparison to prior research on topic modeling performance

Few studies compare topic modeling algorithms according to their actual accuracy, and their findings are mixed [68]. Compare the task performance of PLSA and LDA using two datasets. Their findings show that LDA performs better than PLSA on one dataset and comparable on the other. Both algorithms are outperformed by k-means in their setup. In our experiments, we can confirm the strong performance of k-means, but found significant differences in the performance of PLSA and LDA identifying sampling-based LDA as much more accurate, especially for large numbers of clusters, i.e. topics.

### Comparison to prior research on topic modeling evaluation metrics

Other studies rely on coherence or other internal metrics to evaluate topic modeling procedures. For example [41], find that several coherence measures of topic descriptors produced by NMF are superior to LDA [69]. Assess the interpretability of LDA, LSA and NMF. They conclude that each of the algorithms has different strengths. LDA is best in learning descriptive topics, while LSA is best at creating compact representations of documents and words. However, NMF yields more incoherent topics than LDA and SVD. Therefore, according to [69], LDA should be preferred to NMF. However, both studies rely on coherence only and do not employ any measures for accuracy. As demonstrated in the second part of our study, we find that coherence measures are not correlated to accuracy. Therefore, we would like to suggest that in future studies on the coherence of calculated topics, the accuracy should always be reported in order to be able to better interpret the results.

Other studies examine or question the validity and reliability of topic modeling evaluation metrics. For instance [28], benchmark a number of metrics with regard to their suitability for determining the correct number of topics *k* inherent in a dataset. They compare, among others, the metrics from [32–34]. In their experiments, the latter two metrics show overall poor performance. Our findings are consistent with the results of their study. In their experiments, though, the metric from [32] tends to show accurate results if the topics are well-separable in linear space. However, this characteristic does not relate to our non-artificial datasets, as they use synthetically-generated test data to keep exact control for the properties of the texts.

For assessing the quality of generated topics, several prior studies agree that coherence may be a good candidate [12, 17, 38]. Conversely [13], object that coherence metrics are “less than a perfect guide” (p. 5). They argue that coherence metrics are able to identify poorly-defined topics well but are not good at identifying coherent ones. They speculate that topics may have good coherence values just because they are composed of common words that co-occur frequently in a reference corpus, but do not relate to any concept of discourse *per se*. Although we cannot fully explain the poor performance of coherence metrics, our results agree the statement from [13]. Specifically, our results show that coherence (at least the way it is currently measured in the literature) does not correlate with accuracy. Thus, clustering results that yield coherent topics do not necessarily guarantee correctness. This fact renders analyses relying solely on coherence measures contestable.

In the past, for assessing model quality in general, researchers usually looked at perplexity [5]. According to our results, at least for sampling-based LDA, perplexity is indeed the best predictor of result correctness. In general, lower perplexity scores express the ability of a model to generalize better. However [17], argue that perplexity and human judgment are often not correlated. They show that lower perplexity may result in topics that are exceedingly specific but hard to understand. In other words, perplexity encourages complexity, but this is usually contrary to analysts’ requirements. In their experiments, they tested three topic modeling procedures: PLSA, LDA, and correlated topic model (CTM) [70], on two datasets with values of *k = 50*, *k = 100*, *k = 150*. They examined to what extent the correct identification of an intruder word in the topic descriptors was related to the predictive log-likelihood. Their results show that with simultaneously increasing *k*, in some cases, perplexity correlates negatively with their definition of topic descriptor accuracy. They do not compare their measure to an external validity metric but rely on perplexity only. Thus, the only implication that can be drawn from this is that perplexity may not be related to the employed definition of topic descriptor accuracy. Most importantly, their results indicate that decreasing perplexity is accompanied by an increasing number of clusters *k*. This is to be expected, as perplexity typically drops as the number of topics grows [71]. Therefore, our more detailed experiments may extend their findings to the effect that perplexity and topic coherence are not correlated at all. For some algorithms, topic coherence seems to be a good estimate for convergence, but in general, it is unclear how to use it to evaluate the quality of topic modeling results [18]. State that the use of perplexity is somewhat misguided if the purpose of a model is to help understand the underlying thematic structure and not to correctly predict any document’s class. We concur with this line of reasoning but emphasize that coherence is no guarantee of accuracy.

## Limitations and future research

There are several opportunities for future research to extend our assessment of topic modeling performance and evaluation metrics. First of all, even though we sought to create the most realistic and at the same time most generalizable setting possible, there may be other scenarios in which our assumptions and findings do not hold. As other researchers have pointed out, topic modeling algorithms are highly sensitive to cluster sizes, text lengths, overall text characteristics, and the text preprocessing–none of which are the focus of this study. Hence, further investigations including parameters and characteristics are necessary. A promising approach for this purpose may be the use of synthetic datasets [11, 52]. In such datasets, all text characteristics can be controlled and altered systematically. This approach may allow for exploring the boundaries and interconnectedness of the characteristics regarding their effects on clustering performance. But it necessitates methods for the comparability of the synthetic to real-world datasets to be able to transfer the findings derived from synthetic datasets. If it is possible to identify clear and transferable characteristics of texts and their influence on clustering performance, that would allow users to better tailor their choices of a suitable algorithm, its parameterization, and the accompanying evaluation metrics. However, as [11] state, “synthetic corpora are far from the complexity of real-world corpora“. Thus, the transferability of the results remains uncertain at present. Besides the aspects of text characteristics, we did not adjust other parameters besides the number of clusters *k* [52]. Highlight the importance of the proper setting of a range of parameters, as some algorithms show high sensitivity to certain parameters. This issue might be addressed by grid-searching the overall parameter space, but that increases the complexity (and runtime) of the necessary experiments exponentially. The same applies to varying preprocessing. Any changes in this aspect can have a considerable impact on the results. Although we have ensured, through extensive pretests, that the result order of the procedures is not influenced by preprocessing, absolute assessments of results cannot be obtained in this way. Future research should therefore also focus on the effect of text preprocessing. Finally, by using Wikipedia as a data source, we were only able to test for the correct assignment of the dominant topic per document. Topic models, however, are capable of discovering distributions of many topics per document. Hence, we plan to extend our experiments in this direction in the future. Among others, the challenges will pertain to the generation of corpora containing documents each composed of multiple well-defined topics with a known topic distribution and the identification or development of appropriate evaluation metrics.

## Concluding remarks

With this study, we contribute to research on the application of topic modeling algorithms and reinvestigate the metrics landscape. Our main purpose is to raise the scientific community’s awareness of the key challenges associated with topic modeling by critically examining the accuracy of the currently available procedures and uncover key problems associated with evaluating results. Obtained through benchmarking scenarios consistent with [52], our results indicate the superiority of LDA and NMF, with NMF appearing more appropriate when the expected number of clusters is less than approx. 20 and LDA more appropriate when the expected number of clusters is more than approx. 20. However, we found no evidence to support a recommendation for any of the result assessment metrics under test. Only the perplexity metric may be suitable for evaluating the results of LDA, but even then, only in a limited way. For this reason, manual evaluation of clustering results remains critical and should be conducted with a high degree of care according to the standards of qualitative research. To promote the application of topic modeling, we call on scientists to intensify research into the automatic assessment of topic modeling results. Future research may build on our preliminary steps in this regard. We encourage researchers to keep in mind the relationship between coherence and accuracy. We recommend that future researchers should always include measures of accuracy in their reported results and their interpretation of findings regarding topic models.

## Supporting information

S1 Appendix Details on the study design. (DOCX)
