# Supplementary material for: Topic modeling revisited: New evidence on algorithm performance and quality metrics
Source: PLoS One. 2022 Apr 28;17(4):e0266325. doi: 10.1371/journal.pone.0266325 (PMC9049322; doi:10.1371/journal.pone.0266325)
Supplement: S1 Appendix — (DOCX) [file pone.0266325.s001.docx]

# Appendix: Details on the Study Design

## Algorithm Parameter Settings

Table 3 lists all algorithms included in our experiments, together with parameter settings and sources for further details regarding the implementation and algorithm parameters.

Table 3: Parameter Settings of all Text Clustering Algorithms Included in our Study

| **Algorithm** | **Name** | **Method** | **Parameters** | **Type** | **Reference** |
| --- | --- | --- | --- | --- | --- |
| K-Means | SklearnKmeans | Partitioning | method=k-means++ max_iter=300 | Batch | [61] |
| Latent Semantic Analysis (LSA) | SklearnLsi | Matrix Decomposition (Truncated SVD) | n_iter=5 | Batch | [23, 61] |
| Probabilistic Latent Semantic Analysis (PLSA) | SklearnPlsa | Stochastic Model, see NMF (Ding et al., 2008) | alpha=0.0, l1_ratio=.5, init='nndsvda', solver='mu', beta_loss='kullback-leibler', max_iter=500 | Batch | [6, 61] |
| Non-negative matrix factorization (NMF) | SklearnNmf | Constained Matrix Decomposition | alpha=0.0, l1_ratio=.5, init='nndsvd', max_iter=500 | Batch | [53, 54, 24, 61, 26] |
| Latent Dirichlet Allocation (LDA) | SklearnBatchVbLda | Stochastic Model, Variational Bayes | passes=20 | Batch | [5, 56, 61] |
| Latent Dirichlet Allocation (LDA) | SklearnOnlineVbLda | Stochastic Model, Variational Bayes | passes=20, learning_decay=0.7, learning_offset=10, batch_size=2000 | Online | [5, 56, 61] |
| Latent Dirichlet Allocation (LDA) | GensimBatchVbLda | Stochastic Model, Variational Bayes | doc_topic_prior=0.01, topic_word_prior=50.0, passes=20 | Batch | [5, 60] |
| Latent Dirichlet Allocation (LDA) | GensimOnlineVbLda | Stochastic Model, Variational Bayes | doc_topic_prior=0.01, topic_word_prior=50.0, passes=20, learning_decay=0.7, learning_offset=10, batch_size=2000 | Online | [5, 60] |
| Latent Dirichlet Allocation (LDA) | MalletGibbsLda | Stochastic Model, Sampling | doc_topic_prior=0.01, topic_word_prior=5.0, iterations=1000 | Batch | [5, 27, 57, 59] |

## Dataset Extraction Procedure and Composition

All data used for compiling the datasets were downloaded by means of the Wikipedia Dumps on December 1, 2019. See https://www.mediawiki.org/wiki/API:Main_page for more details. Article extraction and cleaning from Wikicode was carried out using the Python library mwparserfromhell. See <https://github.com/earwig/mwparserfromhell> for more details. Filtering of special purpose pages, usually related to maintenance, was done using the Wikipedia namespaces and the Wikipedia category scheme. An overview of the namespaces and the maintenance categories can be found here: <https://en.wikipedia.org/wiki/Help:Special_page>. All generated datasets have been manually searched for unwanted artifacts. They are made available via our online appendix. Wikipedia article texts are licensed under the [Creative Commons Attribution-ShareAlike 3.0 Unported License](https://en.wikipedia.org/wiki/Wikipedia:Text_of_Creative_Commons_Attribution-ShareAlike_3.0_Unported_License) (CC BY-SA) and can therefore be copied, distributed and/or modified under these terms. Hence, all data collection and sharing complies with the terms and conditions of Wikipedia.

Table 4 contains the Wikipedia categories included in the test series WikiA, WikiB, WikiC, WikiD and WikiE. Each test series includes up to 75 categories for 21 tests. Each test includes 50 (+/- 1) Wikipedia articles of 2, 3, 5, 7, 10, 13, 15, 17, 20, 23, 25, 27, 30, 35, 40, 45, 50, 55, 60, 65, and 75 categories respectively. For example, test 3 of test series WikiC includes 150 articles taken from the 3 Wikipedia categories Binary_stars, Monetary_policy, and Monetary_policy.

Table 4: Wikipedia Categories Contained in the Test Series

| **WikiA** | **WikiB** | **WikiC** | **WikiD** | **WikiE** |
| --- | --- | --- | --- | --- |
| Cybercrime | Programming_paradigms | Binary_stars | Packaging | Painting_techniques |
| Artificial_intelligence | Corporate_finance | Monetary_policy | Behavioural_sciences | Antibiotics |
| Petroleum_production | Artificial_intelligence | Monetary_policy | Cybercrime | Monetary_economics |
| Neurology | Production_economics | Ball_games | Individual_sports | Plate_tectonics |
| Cryptography | Linear_algebra | Photovoltaics | Antibiotics | Monetary_policy |
| Psychoanalysis | Soil | Banking | Medieval_art | Livestock |
| Radioactivity | Church_architecture | Monetary_economics | Theoretical_computer_science | Psychoanalysis |
| Painting_techniques | Aerodynamics | Chemistry | Telegraphy | Church_architecture |
| Neurotoxins | Antibiotics | Thermodynamics | Production_economics | Binary_stars |
| Telegraphy | Theoretical_computer_science | Plate_tectonics | Quantum_mechanics | Economics_models |
| Crystallography | Plate_tectonics | Road_safety | Boat_types | Phobias |
| Political_science | Financial_regulation | Liberalism | Democracy | Feudalism |
| Financial_crises | Industrial_processes | Optics | Critical_theory | Game_theory |
| Linguistics | Livestock | Quantum_mechanics | Liberalism | Financial_regulation |
| Medieval_art | Game_theory | Astrophysics | Robotics | Soil |
| Herbs | Democracy | Greek_mythology | Soil | French_Revolution |
| Economics_models | Critical_theory | Nutrition | Atomic_physics | Immunology |
| Ball_games | Linguistics | Horticulture_and_gardening | Toxicology | Medieval_art |
| Road_safety | Online_advertising | Marine_biology | Russian_Revolution | Petroleum_production |
| Game_theory | Nutrition | Atomic_physics | Central_banks | Linear_algebra |
| Financial_regulation | Horticulture_and_gardening | Corporate_finance | Phobias | Optics |
| Church_architecture | Social_psychology | Democracy | Loans | Central_banks |
| Quantum_mechanics | Individual_sports | Urban_planning | Cryptography | Thermodynamics |
| Organic_reactions | Petroleum_production | Intelligence | Mysticism | Cooking_techniques |
| Feudalism | Astrophysics | Toxicology | Greek_mythology | Greek_mythology |
| Thermodynamics | Financial_crises | Aerodynamics | Livestock | Horticulture_and_gardening |
| Democracy | Neurotoxins | Herbs | Organizational_behavior | Organizational_behavior |
| Cooking_techniques | Russian_Revolution | Behavioural_sciences | Financial_regulation | Stock_market |
| Industrial_processes | Digestive_system | Witchcraft | Intelligence | Road_safety |
| Photosynthesis | Zoology | Financial_crises | Crystallography | Critical_theory |
| Digestive_system | Organizational_behavior | Loans | Financial_crises | Atomic_physics |
| Livestock | Crystallography | Medieval_art | Binary_stars | Political_theories |
| Witchcraft | Economics_models | Critical_theory | Thermodynamics | Democracy |
| Atomic_physics | Photovoltaics | Production_economics | French_Revolution | Robotics |
| Russian_Revolution | Political_theories | Climate | Radioactivity | Marine_biology |
| Plate_tectonics | Binary_stars | Energy_economics | Industrial_processes | Cryptography |
| Tropical_diseases | Road_safety | Livestock | Tropical_diseases | Energy_economics |
| Toxicology | Greek_mythology | Cybercrime | Online_advertising | Nutrition |
| Horticulture_and_gardening | Painting_techniques | Digestive_system | Astrophysics | Aerodynamics |
| Central_banks | Herbs | Individual_sports | Banking | Industrial_processes |
| Online_advertising | Medieval_art | Church_architecture | Church_architecture | Agriculture |
| Information_theory | Phobias | Packaging | Energy_economics | Materials |
| Agriculture | Optics | Russian_Revolution | Linguistics | Liberalism |
| Corporate_finance | Marine_biology | Psychoanalysis | Herbs | Toxicology |
| Energy_economics | Thermodynamics | Social_psychology | Marine_biology | Financial_crises |
| Linear_algebra | Agriculture | Crystallography | Stock_market | Programming_paradigms |
| Evolution | Radioactivity | Petroleum_production | Cooking_techniques | Social_psychology |
| Marine_biology | Evolution | Materials | Nutrition | Linguistics |
| Astrophysics | Stock_market | Immunology | Neurotoxins | Cybercrime |
| Monetary_policy | Cybercrime | Industrial_processes | Corporate_finance | Organic_reactions |
| Loans | Mysticism | Soil | Materials | Neurology |
| Network_protocols | Ball_games | Organizational_behavior | Plate_tectonics | Astrophysics |
| Urban_planning | Liberalism | Theoretical_computer_science | Petroleum_production | Intelligence |
| Soil | Materials | Linguistics | Digestive_system | Crystallography |
| Intelligence | Cryptography | Online_advertising | Political_theories | Radioactivity |
| Theoretical_computer_science | Robotics | Network_protocols | Monetary_policy | Evolution |
| Nutrition | Atomic_physics | Agriculture | Psychoanalysis | Photovoltaics |
| Monetary_economics | Psychoanalysis | Photosynthesis | Optics | Photosynthesis |
| Chemistry | Photosynthesis | Zoology | Agriculture | Theoretical_computer_science |
| Social_psychology | Neurology | Antibiotics | Painting_techniques | Tropical_diseases |
| Packaging | Energy_economics | Military_ranks | Organic_reactions | Boat_types |
| Mysticism | Behavioural_sciences | Artificial_intelligence | Monetary_economics | Urban_planning |
| Political_theories | French_Revolution | Neurology | Chemistry | Online_advertising |
| Photovoltaics | Boat_types | Financial_regulation | Network_protocols | Herbs |
| Critical_theory | Cooking_techniques | Phobias | Neurology | Banking |
| Individual_sports | Political_science | Painting_techniques | Artificial_intelligence | Packaging |
| Antibiotics | Intelligence | Game_theory | Ball_games | Digestive_system |
| Liberalism | Witchcraft | Central_banks | Photovoltaics | Telegraphy |
| Optics | Loans | Stock_market | Evolution | Network_protocols |
| Climate | Immunology | Programming_paradigms | Immunology | Loans |
| Programming_paradigms | Organic_reactions | Political_science | Climate | Neurotoxins |
| Production_economics | Toxicology | Robotics | Information_theory | Witchcraft |
| French_Revolution | Information_theory | Radioactivity | Road_safety | Zoology |
| Banking | Quantum_mechanics | Cryptography | Witchcraft | Quantum_mechanics |
| Robotics | Climate | Economics_models | Feudalism | Mysticism |

# Online Appendix: Experimental Results

Table 5 contains the results for all test series WikiA to WikiE for all algorithms and all included metrics. Note, that not all metrics are properly defined for all algorithms.

Table 5: Experimental Results for all Test Series

| **Dataset** | **Algorithm** | **F1_U** | **F1_O** | **ARI** | **AMI** |  | **Perplexity** | **C_V** | **C_NPMI** | **C_UCI** | **C_UMASS** | **C_W2V** | **Cao09** | **Arun10** | **Deveaud14** |
| --- | --- | --- | --- | --- | --- | --- | --- | --- | --- | --- | --- | --- | --- | --- | --- |
| WikiA2 | SklearnKmeans | 0,961 | 0,961 | 0,850 | 0,799 |  |  | 0,407 | -0,075 | -2,455 | -1,369 | 0,991 | 0,996 | 23,422 | 0,194 |
| WikiA2 | SklearnLsi | 0,811 | 0,811 | 0,396 | 0,446 |  |  | 0,403 | -0,043 | -1,681 | -1,113 | 0,988 |  |  |  |
| WikiA2 | SklearnNmf | 0,980 | 0,980 | 0,920 | 0,876 |  |  | 0,524 | -0,016 | -1,169 | -0,903 | 0,995 | 0,258 | 3,588 | 0,130 |
| WikiA2 | SklearnPlsa | 0,949 | 0,949 | 0,806 | 0,758 |  |  | 0,313 | -0,091 | -2,544 | -0,846 | 0,995 | 0,450 | 3,470 | 0,142 |
| WikiA2 | SklearnBatchVbLda | 0,960 | 0,960 | 0,846 | 0,794 |  | 742,343 | 0,429 | -0,028 | -1,181 | -1,115 | 0,991 | 0,392 | 30,344 | 0,153 |
| WikiA2 | SklearnOnlineVbLda | 0,883 | 0,883 | 0,640 | 0,574 |  | 787,325 | 0,396 | -0,031 | -1,132 | -1,012 | 0,991 | 0,524 | 31,143 | 0,185 |
| WikiA2 | GensimBatchVbLda | 0,924 | 0,924 | 0,749 | 0,692 |  | 741,677 | 0,426 | -0,020 | -0,911 | -0,919 | 0,992 | 0,454 | 30,520 | 0,168 |
| WikiA2 | GensimOnlineVbLda | 0,887 | 0,887 | 0,661 | 0,608 |  | 756,997 | 0,394 | -0,035 | -1,235 | -1,048 | 0,990 | 0,528 | 31,281 | 0,186 |
| WikiA2 | MalletGibbsLda | 0,964 | 0,964 | 0,862 | 0,812 |  | 795,305 | 0,436 | -0,023 | -1,068 | -1,125 | 0,992 | 0,376 | 30,268 | 0,139 |
| WikiA3 | SklearnKmeans | 0,945 | 0,945 | 0,840 | 0,810 |  |  | 0,571 | -0,018 | -1,541 | -1,600 | 0,969 | 0,996 | 32,414 | 0,221 |
| WikiA3 | SklearnLsi | 0,851 | 0,851 | 0,572 | 0,637 |  |  | 0,584 | -0,002 | -1,721 | -1,605 | 0,960 |  |  |  |
| WikiA3 | SklearnNmf | 0,967 | 0,967 | 0,901 | 0,866 |  |  | 0,692 | 0,049 | -0,437 | -0,847 | 0,985 | 0,201 | 3,047 | 0,141 |
| WikiA3 | SklearnPlsa | 0,947 | 0,947 | 0,842 | 0,827 |  |  | 0,391 | -0,030 | -1,136 | -0,883 | 0,971 | 0,456 | 3,187 | 0,164 |
| WikiA3 | SklearnBatchVbLda | 0,889 | 0,898 | 0,758 | 0,739 |  | 511,448 | 0,541 | 0,015 | -0,625 | -0,917 | 0,970 | 0,285 | 35,891 | 0,157 |
| WikiA3 | SklearnOnlineVbLda | 0,748 | 0,794 | 0,583 | 0,624 |  | 537,055 | 0,538 | 0,017 | -0,669 | -1,021 | 0,970 | 0,334 | 36,564 | 0,172 |
| WikiA3 | GensimBatchVbLda | 0,829 | 0,844 | 0,666 | 0,668 |  | 516,360 | 0,529 | 0,010 | -0,748 | -1,050 | 0,968 | 0,320 | 36,295 | 0,168 |
| WikiA3 | GensimOnlineVbLda | 0,806 | 0,845 | 0,663 | 0,679 |  | 513,765 | 0,552 | 0,021 | -0,565 | -0,945 | 0,971 | 0,317 | 36,484 | 0,167 |
| WikiA3 | MalletGibbsLda | 0,948 | 0,948 | 0,846 | 0,812 |  | 552,703 | 0,557 | 0,021 | -0,450 | -0,840 | 0,972 | 0,274 | 35,590 | 0,148 |
| WikiA5 | SklearnKmeans | 0,887 | 0,887 | 0,738 | 0,756 |  |  | 0,528 | 0,014 | -0,895 | -1,387 | 0,927 | 0,997 | 43,275 | 0,266 |
| WikiA5 | SklearnLsi | 0,823 | 0,823 | 0,555 | 0,647 |  |  | 0,573 | 0,000 | -1,720 | -1,871 | 0,921 |  |  |  |
| WikiA5 | SklearnNmf | 0,936 | 0,936 | 0,842 | 0,821 |  |  | 0,698 | 0,057 | -0,463 | -1,036 | 0,954 | 0,185 | 2,025 | 0,158 |
| WikiA5 | SklearnPlsa | 0,896 | 0,896 | 0,757 | 0,739 |  |  | 0,421 | -0,014 | -0,711 | -1,019 | 0,921 | 0,434 | 2,335 | 0,180 |
| WikiA5 | SklearnBatchVbLda | 0,801 | 0,817 | 0,666 | 0,701 |  | 446,574 | 0,566 | 0,036 | -0,289 | -1,074 | 0,932 | 0,278 | 40,600 | 0,169 |
| WikiA5 | SklearnOnlineVbLda | 0,739 | 0,764 | 0,582 | 0,632 |  | 461,400 | 0,524 | 0,018 | -0,669 | -1,155 | 0,918 | 0,320 | 41,277 | 0,185 |
| WikiA5 | GensimBatchVbLda | 0,740 | 0,767 | 0,600 | 0,643 |  | 448,283 | 0,528 | 0,020 | -0,603 | -1,133 | 0,920 | 0,333 | 41,545 | 0,188 |
| WikiA5 | GensimOnlineVbLda | 0,767 | 0,777 | 0,607 | 0,653 |  | 446,914 | 0,536 | 0,026 | -0,459 | -1,052 | 0,921 | 0,310 | 41,377 | 0,181 |
| WikiA5 | MalletGibbsLda | 0,919 | 0,924 | 0,815 | 0,823 |  | 536,743 | 0,585 | 0,045 | -0,020 | -0,945 | 0,931 | 0,287 | 40,745 | 0,170 |
| WikiA7 | SklearnKmeans | 0,809 | 0,820 | 0,639 | 0,709 |  |  | 0,547 | 0,018 | -0,884 | -1,409 | 0,896 | 0,997 | 50,091 | 0,281 |
| WikiA7 | SklearnLsi | 0,630 | 0,638 | 0,335 | 0,524 |  |  | 0,439 | -0,037 | -2,443 | -1,804 | 0,880 |  |  |  |
| WikiA7 | SklearnNmf | 0,856 | 0,856 | 0,692 | 0,727 |  |  | 0,717 | 0,088 | 0,152 | -0,980 | 0,935 | 0,153 | 3,651 | 0,159 |
| WikiA7 | SklearnPlsa | 0,611 | 0,673 | 0,492 | 0,565 |  |  | 0,409 | -0,012 | -0,761 | -0,959 | 0,892 | 0,369 | 2,900 | 0,174 |
| WikiA7 | SklearnBatchVbLda | 0,692 | 0,730 | 0,574 | 0,668 |  | 442,110 | 0,561 | 0,040 | -0,250 | -1,064 | 0,905 | 0,237 | 41,753 | 0,170 |
| WikiA7 | SklearnOnlineVbLda | 0,631 | 0,663 | 0,504 | 0,614 |  | 455,971 | 0,546 | 0,034 | -0,337 | -1,073 | 0,901 | 0,270 | 42,977 | 0,184 |
| WikiA7 | GensimBatchVbLda | 0,683 | 0,715 | 0,547 | 0,646 |  | 437,925 | 0,544 | 0,036 | -0,336 | -1,123 | 0,898 | 0,286 | 42,915 | 0,191 |
| WikiA7 | GensimOnlineVbLda | 0,692 | 0,710 | 0,547 | 0,641 |  | 440,408 | 0,561 | 0,040 | -0,274 | -1,070 | 0,904 | 0,251 | 42,128 | 0,176 |
| WikiA7 | MalletGibbsLda | 0,838 | 0,851 | 0,710 | 0,752 |  | 564,811 | 0,608 | 0,060 | 0,134 | -0,966 | 0,911 | 0,246 | 41,900 | 0,173 |
| WikiA10 | SklearnKmeans | 0,758 | 0,784 | 0,605 | 0,715 |  |  | 0,558 | 0,032 | -0,549 | -1,491 | 0,864 | 0,997 | 59,547 | 0,295 |
| WikiA10 | SklearnLsi | 0,571 | 0,608 | 0,360 | 0,547 |  |  | 0,425 | -0,095 | -4,005 | -2,961 | 0,812 |  |  |  |
| WikiA10 | SklearnNmf | 0,734 | 0,789 | 0,611 | 0,710 |  |  | 0,712 | 0,086 | 0,148 | -1,058 | 0,903 | 0,132 | 3,331 | 0,158 |
| WikiA10 | SklearnPlsa | 0,700 | 0,701 | 0,514 | 0,585 |  |  | 0,404 | -0,017 | -0,881 | -1,138 | 0,857 | 0,324 | 2,369 | 0,168 |
| WikiA10 | SklearnBatchVbLda | 0,671 | 0,701 | 0,536 | 0,663 |  | 399,398 | 0,596 | 0,060 | 0,086 | -1,123 | 0,877 | 0,210 | 46,395 | 0,166 |
| WikiA10 | SklearnOnlineVbLda | 0,603 | 0,632 | 0,472 | 0,608 |  | 409,963 | 0,566 | 0,047 | -0,149 | -1,157 | 0,873 | 0,228 | 47,364 | 0,177 |
| WikiA10 | GensimBatchVbLda | 0,657 | 0,685 | 0,533 | 0,654 |  | 396,454 | 0,572 | 0,052 | -0,040 | -1,144 | 0,868 | 0,258 | 48,276 | 0,190 |
| WikiA10 | GensimOnlineVbLda | 0,624 | 0,653 | 0,498 | 0,631 |  | 396,404 | 0,584 | 0,055 | 0,030 | -1,125 | 0,876 | 0,216 | 46,792 | 0,170 |
| WikiA10 | MalletGibbsLda | 0,758 | 0,772 | 0,617 | 0,714 |  | 544,881 | 0,618 | 0,071 | 0,339 | -1,042 | 0,879 | 0,216 | 46,393 | 0,170 |
| WikiA13 | SklearnKmeans | 0,712 | 0,755 | 0,552 | 0,704 |  |  | 0,547 | 0,033 | -0,446 | -1,412 | 0,840 | 0,997 | 64,420 | 0,304 |
| WikiA13 | SklearnLsi | 0,469 | 0,520 | 0,263 | 0,461 |  |  | 0,391 | -0,100 | -3,862 | -3,127 | 0,781 |  |  |  |
| WikiA13 | SklearnNmf | 0,710 | 0,751 | 0,572 | 0,698 |  |  | 0,699 | 0,084 | 0,221 | -1,128 | 0,889 | 0,115 | 7,881 | 0,156 |
| WikiA13 | SklearnPlsa | 0,592 | 0,598 | 0,404 | 0,523 |  |  | 0,463 | 0,008 | -0,451 | -1,214 | 0,855 | 0,267 | 4,610 | 0,158 |
| WikiA13 | SklearnBatchVbLda | 0,623 | 0,650 | 0,496 | 0,637 |  | 387,487 | 0,591 | 0,059 | 0,140 | -1,161 | 0,860 | 0,199 | 45,780 | 0,164 |
| WikiA13 | SklearnOnlineVbLda | 0,521 | 0,551 | 0,406 | 0,568 |  | 402,720 | 0,572 | 0,049 | -0,051 | -1,178 | 0,857 | 0,198 | 46,091 | 0,167 |
| WikiA13 | GensimBatchVbLda | 0,609 | 0,637 | 0,492 | 0,629 |  | 388,204 | 0,567 | 0,051 | 0,013 | -1,175 | 0,853 | 0,247 | 48,145 | 0,188 |
| WikiA13 | GensimOnlineVbLda | 0,577 | 0,606 | 0,455 | 0,607 |  | 389,730 | 0,589 | 0,056 | 0,064 | -1,157 | 0,861 | 0,184 | 45,478 | 0,158 |
| WikiA13 | MalletGibbsLda | 0,739 | 0,748 | 0,586 | 0,700 |  | 567,202 | 0,615 | 0,071 | 0,397 | -1,094 | 0,864 | 0,210 | 45,928 | 0,169 |
| WikiA15 | SklearnKmeans | 0,680 | 0,712 | 0,490 | 0,668 |  |  | 0,535 | 0,022 | -0,617 | -1,268 | 0,824 | 0,997 | 69,979 | 0,310 |
| WikiA15 | SklearnLsi | 0,529 | 0,547 | 0,334 | 0,506 |  |  | 0,370 | -0,092 | -3,361 | -2,384 | 0,767 |  |  |  |
| WikiA15 | SklearnNmf | 0,728 | 0,766 | 0,583 | 0,717 |  |  | 0,690 | 0,086 | 0,386 | -1,131 | 0,883 | 0,109 | 8,847 | 0,154 |
| WikiA15 | SklearnPlsa | 0,588 | 0,588 | 0,390 | 0,508 |  |  | 0,452 | 0,008 | -0,416 | -1,190 | 0,842 | 0,230 | 5,441 | 0,152 |
| WikiA15 | SklearnBatchVbLda | 0,598 | 0,636 | 0,491 | 0,644 |  | 364,642 | 0,581 | 0,058 | 0,215 | -1,158 | 0,850 | 0,183 | 48,632 | 0,162 |
| WikiA15 | SklearnOnlineVbLda | 0,517 | 0,549 | 0,423 | 0,592 |  | 376,296 | 0,567 | 0,051 | 0,046 | -1,188 | 0,851 | 0,175 | 48,563 | 0,162 |
| WikiA15 | GensimBatchVbLda | 0,580 | 0,608 | 0,474 | 0,626 |  | 365,232 | 0,552 | 0,047 | 0,023 | -1,181 | 0,842 | 0,230 | 51,282 | 0,189 |
| WikiA15 | GensimOnlineVbLda | 0,553 | 0,582 | 0,449 | 0,615 |  | 365,467 | 0,579 | 0,057 | 0,171 | -1,164 | 0,853 | 0,161 | 47,533 | 0,154 |
| WikiA15 | MalletGibbsLda | 0,698 | 0,725 | 0,563 | 0,689 |  | 551,960 | 0,604 | 0,071 | 0,451 | -1,103 | 0,853 | 0,192 | 48,226 | 0,170 |
| WikiA17 | SklearnKmeans | 0,673 | 0,707 | 0,476 | 0,663 |  |  | 0,516 | 0,028 | -0,416 | -1,455 | 0,809 | 0,997 | 75,454 | 0,313 |
| WikiA17 | SklearnLsi | 0,588 | 0,599 | 0,412 | 0,563 |  |  | 0,401 | -0,090 | -3,527 | -2,596 | 0,763 |  |  |  |
| WikiA17 | SklearnNmf | 0,737 | 0,761 | 0,599 | 0,709 |  |  | 0,665 | 0,080 | 0,336 | -1,186 | 0,862 | 0,100 | 11,279 | 0,152 |
| WikiA17 | SklearnPlsa | 0,544 | 0,556 | 0,392 | 0,518 |  |  | 0,461 | 0,017 | -0,250 | -1,232 | 0,835 | 0,200 | 9,874 | 0,147 |
| WikiA17 | SklearnBatchVbLda | 0,630 | 0,655 | 0,521 | 0,671 |  | 360,562 | 0,582 | 0,061 | 0,260 | -1,167 | 0,837 | 0,171 | 49,128 | 0,160 |
| WikiA17 | SklearnOnlineVbLda | 0,493 | 0,521 | 0,409 | 0,584 |  | 375,763 | 0,563 | 0,051 | 0,049 | -1,204 | 0,839 | 0,150 | 48,779 | 0,156 |
| WikiA17 | GensimBatchVbLda | 0,584 | 0,617 | 0,491 | 0,646 |  | 362,564 | 0,553 | 0,051 | 0,086 | -1,182 | 0,829 | 0,218 | 51,966 | 0,187 |
| WikiA17 | GensimOnlineVbLda | 0,523 | 0,555 | 0,442 | 0,611 |  | 364,816 | 0,573 | 0,056 | 0,127 | -1,191 | 0,842 | 0,141 | 47,486 | 0,147 |
| WikiA17 | MalletGibbsLda | 0,704 | 0,724 | 0,578 | 0,703 |  | 557,035 | 0,601 | 0,072 | 0,454 | -1,120 | 0,840 | 0,187 | 49,582 | 0,169 |
| WikiA20 | SklearnKmeans | 0,650 | 0,677 | 0,467 | 0,661 |  |  | 0,491 | 0,025 | -0,379 | -1,303 | 0,778 | 0,996 | 78,950 | 0,314 |
| WikiA20 | SklearnLsi | 0,526 | 0,547 | 0,392 | 0,553 |  |  | 0,347 | -0,088 | -3,110 | -2,097 | 0,719 |  |  |  |
| WikiA20 | SklearnNmf | 0,696 | 0,711 | 0,535 | 0,687 |  |  | 0,624 | 0,070 | 0,239 | -1,261 | 0,834 | 0,081 | 19,523 | 0,150 |
| WikiA20 | SklearnPlsa | 0,480 | 0,504 | 0,323 | 0,468 |  |  | 0,467 | 0,011 | -0,504 | -1,328 | 0,810 | 0,169 | 16,187 | 0,140 |
| WikiA20 | SklearnBatchVbLda | 0,570 | 0,605 | 0,477 | 0,648 |  | 342,705 | 0,553 | 0,054 | 0,171 | -1,226 | 0,809 | 0,147 | 48,097 | 0,156 |
| WikiA20 | SklearnOnlineVbLda | 0,448 | 0,474 | 0,373 | 0,571 |  | 356,016 | 0,537 | 0,044 | -0,052 | -1,261 | 0,812 | 0,126 | 47,677 | 0,148 |
| WikiA20 | GensimBatchVbLda | 0,538 | 0,574 | 0,448 | 0,624 |  | 344,758 | 0,533 | 0,048 | 0,085 | -1,219 | 0,801 | 0,190 | 51,868 | 0,183 |
| WikiA20 | GensimOnlineVbLda | 0,476 | 0,504 | 0,397 | 0,590 |  | 344,730 | 0,555 | 0,052 | 0,090 | -1,241 | 0,816 | 0,113 | 46,182 | 0,140 |
| WikiA20 | MalletGibbsLda | 0,655 | 0,685 | 0,532 | 0,684 |  | 537,051 | 0,582 | 0,068 | 0,443 | -1,184 | 0,815 | 0,162 | 48,968 | 0,165 |
| WikiA23 | SklearnKmeans | 0,619 | 0,647 | 0,434 | 0,643 |  |  | 0,482 | 0,025 | -0,310 | -1,250 | 0,760 | 0,996 | 83,859 | 0,318 |
| WikiA23 | SklearnLsi | 0,511 | 0,524 | 0,354 | 0,521 |  |  | 0,360 | -0,084 | -3,018 | -2,136 | 0,712 |  |  |  |
| WikiA23 | SklearnNmf | 0,587 | 0,600 | 0,437 | 0,609 |  |  | 0,621 | 0,070 | 0,252 | -1,287 | 0,828 | 0,073 | 29,497 | 0,146 |
| WikiA23 | SklearnPlsa | 0,461 | 0,473 | 0,310 | 0,449 |  |  | 0,472 | 0,016 | -0,358 | -1,301 | 0,800 | 0,143 | 25,147 | 0,134 |
| WikiA23 | SklearnBatchVbLda | 0,551 | 0,579 | 0,454 | 0,638 |  | 334,815 | 0,558 | 0,058 | 0,239 | -1,227 | 0,798 | 0,138 | 49,119 | 0,153 |
| WikiA23 | SklearnOnlineVbLda | 0,420 | 0,442 | 0,338 | 0,551 |  | 352,376 | 0,528 | 0,043 | -0,016 | -1,263 | 0,796 | 0,116 | 48,595 | 0,142 |
| WikiA23 | GensimBatchVbLda | 0,531 | 0,561 | 0,441 | 0,631 |  | 337,671 | 0,532 | 0,049 | 0,127 | -1,230 | 0,787 | 0,180 | 52,879 | 0,181 |
| WikiA23 | GensimOnlineVbLda | 0,437 | 0,465 | 0,363 | 0,574 |  | 341,638 | 0,543 | 0,048 | 0,023 | -1,263 | 0,805 | 0,100 | 46,336 | 0,132 |
| WikiA23 | MalletGibbsLda | 0,651 | 0,676 | 0,524 | 0,683 |  | 543,857 | 0,586 | 0,071 | 0,478 | -1,192 | 0,805 | 0,157 | 49,656 | 0,162 |
| WikiA25 | SklearnKmeans | 0,598 | 0,629 | 0,415 | 0,632 |  |  | 0,477 | 0,024 | -0,325 | -1,208 | 0,764 | 0,996 | 89,529 | 0,320 |
| WikiA25 | SklearnLsi | 0,460 | 0,483 | 0,347 | 0,513 |  |  | 0,326 | -0,096 | -3,216 | -2,281 | 0,704 |  |  |  |
| WikiA25 | SklearnNmf | 0,557 | 0,597 | 0,427 | 0,613 |  |  | 0,600 | 0,060 | 0,150 | -1,292 | 0,822 | 0,071 | 31,939 | 0,147 |
| WikiA25 | SklearnPlsa | 0,394 | 0,409 | 0,283 | 0,421 |  |  | 0,469 | 0,012 | -0,450 | -1,341 | 0,805 | 0,108 | 48,549 | 0,124 |
| WikiA25 | SklearnBatchVbLda | 0,563 | 0,591 | 0,458 | 0,641 |  | 327,517 | 0,544 | 0,054 | 0,242 | -1,214 | 0,798 | 0,136 | 51,153 | 0,152 |
| WikiA25 | SklearnOnlineVbLda | 0,415 | 0,440 | 0,343 | 0,560 |  | 344,046 | 0,522 | 0,040 | -0,046 | -1,278 | 0,800 | 0,111 | 51,559 | 0,141 |
| WikiA25 | GensimBatchVbLda | 0,522 | 0,555 | 0,433 | 0,622 |  | 331,055 | 0,517 | 0,046 | 0,135 | -1,223 | 0,788 | 0,175 | 55,436 | 0,180 |
| WikiA25 | GensimOnlineVbLda | 0,446 | 0,473 | 0,366 | 0,577 |  | 333,889 | 0,534 | 0,046 | 0,058 | -1,267 | 0,803 | 0,091 | 47,262 | 0,130 |
| WikiA25 | MalletGibbsLda | 0,665 | 0,686 | 0,522 | 0,681 |  | 531,951 | 0,568 | 0,065 | 0,444 | -1,172 | 0,801 | 0,153 | 52,130 | 0,162 |
| WikiA27 | SklearnKmeans | 0,573 | 0,604 | 0,362 | 0,600 |  |  | 0,468 | 0,021 | -0,352 | -1,243 | 0,753 | 0,997 | 91,273 | 0,324 |
| WikiA27 | SklearnLsi | 0,384 | 0,410 | 0,303 | 0,479 |  |  | 0,326 | -0,094 | -3,108 | -2,138 | 0,696 |  |  |  |
| WikiA27 | SklearnNmf | 0,555 | 0,581 | 0,400 | 0,594 |  |  | 0,590 | 0,060 | 0,189 | -1,326 | 0,813 | 0,067 | 50,154 | 0,145 |
| WikiA27 | SklearnPlsa | 0,386 | 0,396 | 0,265 | 0,417 |  |  | 0,476 | 0,017 | -0,365 | -1,380 | 0,805 | 0,095 | 67,678 | 0,121 |
| WikiA27 | SklearnBatchVbLda | 0,524 | 0,552 | 0,424 | 0,618 |  | 327,800 | 0,540 | 0,054 | 0,253 | -1,244 | 0,793 | 0,129 | 50,743 | 0,150 |
| WikiA27 | SklearnOnlineVbLda | 0,383 | 0,405 | 0,306 | 0,523 |  | 344,860 | 0,518 | 0,041 | -0,001 | -1,297 | 0,795 | 0,101 | 49,005 | 0,137 |
| WikiA27 | GensimBatchVbLda | 0,491 | 0,522 | 0,400 | 0,603 |  | 332,115 | 0,514 | 0,044 | 0,096 | -1,252 | 0,782 | 0,170 | 55,763 | 0,178 |
| WikiA27 | GensimOnlineVbLda | 0,403 | 0,427 | 0,319 | 0,538 |  | 334,477 | 0,532 | 0,046 | 0,065 | -1,287 | 0,802 | 0,092 | 47,112 | 0,128 |
| WikiA27 | MalletGibbsLda | 0,591 | 0,618 | 0,460 | 0,640 |  | 556,127 | 0,561 | 0,064 | 0,437 | -1,211 | 0,797 | 0,147 | 51,472 | 0,159 |
| WikiA30 | SklearnKmeans | 0,562 | 0,591 | 0,354 | 0,591 |  |  | 0,469 | 0,021 | -0,283 | -1,240 | 0,726 | 0,997 | 96,987 | 0,325 |
| WikiA30 | SklearnLsi | 0,372 | 0,409 | 0,270 | 0,455 |  |  | 0,289 | -0,104 | -3,165 | -2,145 | 0,659 |  |  |  |
| WikiA30 | SklearnNmf | 0,510 | 0,553 | 0,367 | 0,574 |  |  | 0,570 | 0,054 | 0,157 | -1,332 | 0,790 | 0,061 | 65,579 | 0,143 |
| WikiA30 | SklearnPlsa | 0,324 | 0,336 | 0,217 | 0,380 |  |  | 0,461 | 0,007 | -0,580 | -1,437 | 0,792 | 0,067 | 110,910 | 0,112 |
| WikiA30 | SklearnBatchVbLda | 0,515 | 0,542 | 0,409 | 0,611 |  | 319,413 | 0,532 | 0,052 | 0,267 | -1,249 | 0,773 | 0,121 | 49,857 | 0,149 |
| WikiA30 | SklearnOnlineVbLda | 0,358 | 0,383 | 0,294 | 0,520 |  | 336,295 | 0,514 | 0,041 | 0,034 | -1,295 | 0,777 | 0,096 | 50,819 | 0,136 |
| WikiA30 | GensimBatchVbLda | 0,479 | 0,511 | 0,394 | 0,600 |  | 323,737 | 0,507 | 0,043 | 0,121 | -1,248 | 0,761 | 0,159 | 55,056 | 0,177 |
| WikiA30 | GensimOnlineVbLda | 0,389 | 0,415 | 0,312 | 0,537 |  | 324,474 | 0,524 | 0,044 | 0,082 | -1,290 | 0,783 | 0,080 | 45,566 | 0,126 |
| WikiA30 | MalletGibbsLda | 0,599 | 0,624 | 0,464 | 0,646 |  | 538,211 | 0,548 | 0,061 | 0,444 | -1,221 | 0,773 | 0,142 | 51,614 | 0,160 |
| WikiA35 | SklearnKmeans | 0,509 | 0,543 | 0,322 | 0,565 |  |  | 0,466 | 0,027 | -0,087 | -1,220 | 0,710 | 0,997 | 105,550 | 0,329 |
| WikiA35 | SklearnLsi | 0,351 | 0,376 | 0,239 | 0,413 |  |  | 0,284 | -0,101 | -3,020 | -2,104 | 0,644 |  |  |  |
| WikiA35 | SklearnNmf | 0,460 | 0,492 | 0,319 | 0,533 |  |  | 0,558 | 0,051 | 0,136 | -1,363 | 0,777 | 0,055 | 100,124 | 0,141 |
| WikiA35 | SklearnPlsa | 0,297 | 0,310 | 0,199 | 0,345 |  |  | 0,454 | 0,008 | -0,527 | -1,434 | 0,774 | 0,051 | 164,439 | 0,108 |
| WikiA35 | SklearnBatchVbLda | 0,489 | 0,516 | 0,382 | 0,592 |  | 319,987 | 0,532 | 0,054 | 0,303 | -1,264 | 0,761 | 0,112 | 51,180 | 0,146 |
| WikiA35 | SklearnOnlineVbLda | 0,330 | 0,352 | 0,268 | 0,503 |  | 338,485 | 0,506 | 0,038 | 0,002 | -1,318 | 0,765 | 0,086 | 53,192 | 0,130 |
| WikiA35 | GensimBatchVbLda | 0,475 | 0,499 | 0,375 | 0,588 |  | 324,240 | 0,511 | 0,047 | 0,232 | -1,245 | 0,749 | 0,150 | 56,982 | 0,174 |
| WikiA35 | GensimOnlineVbLda | 0,354 | 0,377 | 0,281 | 0,514 |  | 328,112 | 0,512 | 0,038 | -0,053 | -1,337 | 0,772 | 0,073 | 48,888 | 0,121 |
| WikiA35 | MalletGibbsLda | 0,577 | 0,601 | 0,438 | 0,633 |  | 554,046 | 0,547 | 0,062 | 0,474 | -1,227 | 0,758 | 0,137 | 54,160 | 0,158 |
| WikiA40 | SklearnKmeans | 0,492 | 0,523 | 0,323 | 0,552 |  |  | 0,446 | 0,020 | -0,193 | -1,219 | 0,686 | 0,997 | 113,323 | 0,335 |
| WikiA40 | SklearnLsi | 0,327 | 0,346 | 0,207 | 0,379 |  |  | 0,294 | -0,080 | -2,449 | -1,899 | 0,635 |  |  |  |
| WikiA40 | SklearnNmf | 0,447 | 0,489 | 0,316 | 0,521 |  |  | 0,534 | 0,043 | 0,037 | -1,430 | 0,756 | 0,048 | 44,418 | 0,137 |
| WikiA40 | SklearnPlsa | 0,256 | 0,268 | 0,180 | 0,325 |  |  | 0,464 | 0,013 | -0,490 | -1,507 | 0,773 | 0,032 | 265,865 | 0,100 |
| WikiA40 | SklearnBatchVbLda | 0,461 | 0,488 | 0,362 | 0,570 |  | 321,787 | 0,513 | 0,049 | 0,277 | -1,284 | 0,742 | 0,101 | 51,027 | 0,142 |
| WikiA40 | SklearnOnlineVbLda | 0,306 | 0,324 | 0,241 | 0,471 |  | 340,899 | 0,494 | 0,037 | 0,023 | -1,342 | 0,750 | 0,067 | 47,996 | 0,123 |
| WikiA40 | GensimBatchVbLda | 0,451 | 0,475 | 0,354 | 0,566 |  | 327,291 | 0,498 | 0,044 | 0,210 | -1,278 | 0,730 | 0,142 | 58,888 | 0,172 |
| WikiA40 | GensimOnlineVbLda | 0,333 | 0,353 | 0,257 | 0,483 |  | 327,155 | 0,508 | 0,040 | 0,025 | -1,345 | 0,758 | 0,058 | 47,054 | 0,116 |
| WikiA40 | MalletGibbsLda | 0,552 | 0,574 | 0,419 | 0,607 |  | 577,448 | 0,532 | 0,059 | 0,451 | -1,244 | 0,740 | 0,131 | 55,268 | 0,156 |
| WikiA45 | SklearnKmeans | 0,447 | 0,484 | 0,278 | 0,523 |  |  | 0,451 | 0,029 | 0,070 | -1,156 | 0,676 | 0,997 | 121,242 | 0,336 |
| WikiA45 | SklearnLsi | 0,312 | 0,337 | 0,206 | 0,372 |  |  | 0,259 | -0,096 | -2,776 | -2,069 | 0,612 |  |  |  |
| WikiA45 | SklearnNmf | 0,414 | 0,440 | 0,258 | 0,476 |  |  | 0,526 | 0,042 | 0,082 | -1,410 | 0,740 | 0,042 | 44,719 | 0,134 |
| WikiA45 | SklearnPlsa | 0,214 | 0,226 | 0,157 | 0,299 |  |  | 0,445 | 0,001 | -0,752 | -1,601 | 0,764 | 0,021 | 400,999 | 0,095 |
| WikiA45 | SklearnBatchVbLda | 0,441 | 0,467 | 0,334 | 0,552 |  | 320,752 | 0,505 | 0,048 | 0,306 | -1,289 | 0,724 | 0,093 | 51,955 | 0,141 |
| WikiA45 | SklearnOnlineVbLda | 0,287 | 0,307 | 0,223 | 0,457 |  | 342,181 | 0,482 | 0,035 | 0,040 | -1,345 | 0,731 | 0,071 | 58,011 | 0,124 |
| WikiA45 | GensimBatchVbLda | 0,427 | 0,452 | 0,325 | 0,549 |  | 326,999 | 0,488 | 0,043 | 0,230 | -1,270 | 0,711 | 0,133 | 59,809 | 0,172 |
| WikiA45 | GensimOnlineVbLda | 0,294 | 0,313 | 0,230 | 0,466 |  | 329,036 | 0,480 | 0,031 | -0,093 | -1,366 | 0,737 | 0,052 | 54,083 | 0,112 |
| WikiA45 | MalletGibbsLda | 0,527 | 0,547 | 0,389 | 0,587 |  | 593,808 | 0,521 | 0,057 | 0,459 | -1,244 | 0,722 | 0,123 | 55,953 | 0,154 |
| WikiA50 | SklearnKmeans | 0,433 | 0,467 | 0,264 | 0,508 |  |  | 0,443 | 0,027 | 0,027 | -1,186 | 0,660 | 0,997 | 125,284 | 0,336 |
| WikiA50 | SklearnLsi | 0,280 | 0,304 | 0,180 | 0,348 |  |  | 0,253 | -0,097 | -2,797 | -2,010 | 0,599 |  |  |  |
| WikiA50 | SklearnNmf | 0,399 | 0,428 | 0,255 | 0,468 |  |  | 0,519 | 0,044 | 0,138 | -1,435 | 0,725 | 0,038 | 41,663 | 0,130 |
| WikiA50 | SklearnPlsa | 0,176 | 0,186 | 0,119 | 0,250 |  |  | 0,443 | 0,005 | -0,643 | -1,595 | 0,750 | 0,016 | 517,971 | 0,092 |
| WikiA50 | SklearnBatchVbLda | 0,421 | 0,448 | 0,310 | 0,531 |  | 319,706 | 0,502 | 0,049 | 0,327 | -1,284 | 0,709 | 0,088 | 50,318 | 0,138 |
| WikiA50 | SklearnOnlineVbLda | 0,270 | 0,286 | 0,207 | 0,444 |  | 339,146 | 0,476 | 0,034 | 0,037 | -1,352 | 0,714 | 0,069 | 63,054 | 0,122 |
| WikiA50 | GensimBatchVbLda | 0,393 | 0,418 | 0,296 | 0,522 |  | 326,372 | 0,484 | 0,043 | 0,245 | -1,269 | 0,695 | 0,125 | 58,982 | 0,170 |
| WikiA50 | GensimOnlineVbLda | 0,282 | 0,295 | 0,216 | 0,453 |  | 324,691 | 0,473 | 0,028 | -0,158 | -1,382 | 0,721 | 0,055 | 79,650 | 0,113 |
| WikiA50 | MalletGibbsLda | 0,505 | 0,529 | 0,371 | 0,575 |  | 594,322 | 0,516 | 0,056 | 0,460 | -1,245 | 0,706 | 0,118 | 55,820 | 0,152 |
| WikiA55 | SklearnKmeans | 0,410 | 0,443 | 0,234 | 0,481 |  |  | 0,430 | 0,022 | -0,079 | -1,253 | 0,648 | 0,997 | 129,575 | 0,339 |
| WikiA55 | SklearnLsi | 0,302 | 0,316 | 0,192 | 0,363 |  |  | 0,263 | -0,080 | -2,261 | -1,989 | 0,600 |  |  |  |
| WikiA55 | SklearnNmf | 0,374 | 0,398 | 0,236 | 0,450 |  |  | 0,500 | 0,041 | 0,140 | -1,436 | 0,719 | 0,033 | 39,537 | 0,129 |
| WikiA55 | SklearnPlsa | 0,182 | 0,190 | 0,118 | 0,250 |  |  | 0,428 | -0,002 | -0,735 | -1,661 | 0,744 | 0,012 | 696,385 | 0,090 |
| WikiA55 | SklearnBatchVbLda | 0,400 | 0,425 | 0,286 | 0,511 |  | 309,149 | 0,494 | 0,047 | 0,327 | -1,301 | 0,706 | 0,078 | 48,101 | 0,136 |
| WikiA55 | SklearnOnlineVbLda | 0,242 | 0,257 | 0,179 | 0,420 |  | 332,855 | 0,461 | 0,030 | 0,005 | -1,378 | 0,711 | 0,072 | 76,073 | 0,122 |
| WikiA55 | GensimBatchVbLda | 0,388 | 0,413 | 0,283 | 0,514 |  | 314,657 | 0,477 | 0,042 | 0,256 | -1,284 | 0,692 | 0,113 | 56,957 | 0,168 |
| WikiA55 | GensimOnlineVbLda | 0,246 | 0,263 | 0,188 | 0,427 |  | 318,235 | 0,455 | 0,025 | -0,134 | -1,405 | 0,720 | 0,051 | 80,679 | 0,110 |
| WikiA55 | MalletGibbsLda | 0,499 | 0,522 | 0,351 | 0,557 |  | 583,829 | 0,509 | 0,054 | 0,434 | -1,265 | 0,702 | 0,112 | 54,834 | 0,151 |
| WikiA60 | SklearnKmeans | 0,383 | 0,414 | 0,225 | 0,463 |  |  | 0,431 | 0,023 | -0,060 | -1,229 | 0,635 | 0,997 | 134,935 | 0,338 |
| WikiA60 | SklearnLsi | 0,252 | 0,269 | 0,150 | 0,319 |  |  | 0,244 | -0,081 | -2,270 | -1,979 | 0,585 |  |  |  |
| WikiA60 | SklearnNmf | 0,347 | 0,371 | 0,200 | 0,417 |  |  | 0,507 | 0,045 | 0,193 | -1,460 | 0,710 | 0,031 | 41,516 | 0,127 |
| WikiA60 | SklearnPlsa | 0,156 | 0,167 | 0,101 | 0,232 |  |  | 0,425 | -0,002 | -0,739 | -1,674 | 0,736 | 0,009 | 850,513 | 0,089 |
| WikiA60 | SklearnBatchVbLda | 0,366 | 0,389 | 0,258 | 0,488 |  | 315,500 | 0,495 | 0,049 | 0,344 | -1,314 | 0,696 | 0,067 | 46,811 | 0,132 |
| WikiA60 | SklearnOnlineVbLda | 0,222 | 0,236 | 0,169 | 0,411 |  | 337,413 | 0,461 | 0,032 | 0,025 | -1,391 | 0,699 | 0,068 | 90,920 | 0,121 |
| WikiA60 | GensimBatchVbLda | 0,361 | 0,386 | 0,258 | 0,494 |  | 321,503 | 0,478 | 0,043 | 0,277 | -1,294 | 0,680 | 0,106 | 57,608 | 0,166 |
| WikiA60 | GensimOnlineVbLda | 0,230 | 0,245 | 0,173 | 0,413 |  | 319,781 | 0,459 | 0,027 | -0,127 | -1,413 | 0,712 | 0,046 | 95,978 | 0,108 |
| WikiA60 | MalletGibbsLda | 0,457 | 0,479 | 0,315 | 0,530 |  | 608,233 | 0,514 | 0,057 | 0,471 | -1,274 | 0,693 | 0,101 | 54,331 | 0,147 |
| WikiA65 | SklearnKmeans | 0,370 | 0,404 | 0,219 | 0,458 |  |  | 0,434 | 0,026 | 0,066 | -1,163 | 0,634 | 0,997 | 136,339 | 0,340 |
| WikiA65 | SklearnLsi | 0,232 | 0,253 | 0,132 | 0,290 |  |  | 0,237 | -0,079 | -2,117 | -1,963 | 0,572 |  |  |  |
| WikiA65 | SklearnNmf | 0,327 | 0,353 | 0,186 | 0,404 |  |  | 0,493 | 0,040 | 0,165 | -1,491 | 0,704 | 0,027 | 38,091 | 0,124 |
| WikiA65 | SklearnPlsa | 0,143 | 0,152 | 0,091 | 0,214 |  |  | 0,409 | -0,007 | -0,791 | -1,703 | 0,725 | 0,008 | 1049,848 | 0,088 |
| WikiA65 | SklearnBatchVbLda | 0,356 | 0,378 | 0,255 | 0,485 |  | 312,126 | 0,480 | 0,044 | 0,319 | -1,313 | 0,689 | 0,064 | 44,047 | 0,131 |
| WikiA65 | SklearnOnlineVbLda | 0,210 | 0,222 | 0,160 | 0,402 |  | 339,607 | 0,441 | 0,024 | -0,053 | -1,407 | 0,691 | 0,068 | 100,366 | 0,118 |
| WikiA65 | GensimBatchVbLda | 0,349 | 0,370 | 0,252 | 0,488 |  | 318,084 | 0,464 | 0,039 | 0,266 | -1,288 | 0,672 | 0,101 | 55,540 | 0,165 |
| WikiA65 | GensimOnlineVbLda | 0,219 | 0,232 | 0,159 | 0,401 |  | 319,766 | 0,434 | 0,018 | -0,243 | -1,453 | 0,698 | 0,045 | 98,329 | 0,106 |
| WikiA65 | MalletGibbsLda | 0,427 | 0,446 | 0,297 | 0,513 |  | 614,709 | 0,499 | 0,052 | 0,441 | -1,275 | 0,685 | 0,100 | 53,187 | 0,147 |
| WikiA70 | SklearnKmeans | 0,363 | 0,395 | 0,211 | 0,451 |  |  | 0,430 | 0,025 | 0,057 | -1,182 | 0,622 | 0,997 | 142,632 | 0,341 |
| WikiA70 | SklearnLsi | 0,228 | 0,245 | 0,132 | 0,288 |  |  | 0,240 | -0,069 | -1,828 | -1,898 | 0,563 |  |  |  |
| WikiA70 | SklearnNmf | 0,310 | 0,339 | 0,169 | 0,389 |  |  | 0,496 | 0,042 | 0,206 | -1,492 | 0,696 | 0,025 | 38,773 | 0,123 |
| WikiA70 | SklearnPlsa | 0,131 | 0,138 | 0,086 | 0,207 |  |  | 0,401 | -0,010 | -0,851 | -1,726 | 0,716 | 0,006 | 1223,228 | 0,087 |
| WikiA70 | SklearnBatchVbLda | 0,338 | 0,362 | 0,236 | 0,472 |  | 313,753 | 0,475 | 0,044 | 0,315 | -1,321 | 0,679 | 0,059 | 43,762 | 0,129 |
| WikiA70 | SklearnOnlineVbLda | 0,193 | 0,205 | 0,144 | 0,389 |  | 341,145 | 0,437 | 0,024 | -0,043 | -1,415 | 0,682 | 0,067 | 111,692 | 0,118 |
| WikiA70 | GensimBatchVbLda | 0,325 | 0,347 | 0,234 | 0,476 |  | 319,950 | 0,458 | 0,039 | 0,265 | -1,294 | 0,662 | 0,096 | 56,646 | 0,164 |
| WikiA70 | GensimOnlineVbLda | 0,206 | 0,221 | 0,152 | 0,392 |  | 317,470 | 0,435 | 0,017 | -0,272 | -1,465 | 0,695 | 0,038 | 93,291 | 0,104 |
| WikiA70 | MalletGibbsLda | 0,410 | 0,431 | 0,281 | 0,504 |  | 620,047 | 0,496 | 0,051 | 0,435 | -1,288 | 0,676 | 0,093 | 52,309 | 0,145 |
| WikiA75 | SklearnKmeans | 0,342 | 0,373 | 0,197 | 0,436 |  |  | 0,433 | 0,027 | 0,080 | -1,180 | 0,615 | 0,997 | 144,993 | 0,340 |
| WikiA75 | SklearnLsi | 0,217 | 0,231 | 0,114 | 0,264 |  |  | 0,224 | -0,074 | -1,918 | -1,940 | 0,557 |  |  |  |
| WikiA75 | SklearnNmf | 0,284 | 0,305 | 0,151 | 0,365 |  |  | 0,496 | 0,044 | 0,228 | -1,505 | 0,690 | 0,023 | 38,779 | 0,120 |
| WikiA75 | SklearnPlsa | 0,123 | 0,131 | 0,079 | 0,195 |  |  | 0,397 | -0,010 | -0,851 | -1,742 | 0,711 | 0,005 | 1442,056 | 0,086 |
| WikiA75 | SklearnBatchVbLda | 0,316 | 0,338 | 0,220 | 0,458 |  | 319,450 | 0,478 | 0,045 | 0,341 | -1,320 | 0,674 | 0,055 | 43,008 | 0,127 |
| WikiA75 | SklearnOnlineVbLda | 0,184 | 0,196 | 0,138 | 0,382 |  | 346,099 | 0,438 | 0,026 | -0,006 | -1,418 | 0,676 | 0,063 | 115,929 | 0,116 |
| WikiA75 | GensimBatchVbLda | 0,313 | 0,335 | 0,223 | 0,466 |  | 325,578 | 0,461 | 0,040 | 0,275 | -1,300 | 0,656 | 0,090 | 56,437 | 0,162 |
| WikiA75 | GensimOnlineVbLda | 0,193 | 0,205 | 0,142 | 0,384 |  | 321,908 | 0,429 | 0,015 | -0,304 | -1,477 | 0,686 | 0,038 | 122,317 | 0,103 |
| WikiA75 | MalletGibbsLda | 0,384 | 0,406 | 0,261 | 0,490 |  | 637,999 | 0,499 | 0,053 | 0,456 | -1,286 | 0,670 | 0,089 | 51,593 | 0,143 |
| WikiB2 | SklearnKmeans | 0,996 | 0,996 | 0,982 | 0,969 |  |  | 0,516 | -0,026 | -1,571 | -1,047 | 0,958 | 0,986 | 25,248 | 0,167 |
| WikiB2 | SklearnLsi | 0,779 | 0,779 | 0,326 | 0,398 |  |  | 0,524 | -0,018 | -1,834 | -1,639 | 0,925 |  |  |  |
| WikiB2 | SklearnNmf | 0,990 | 0,990 | 0,960 | 0,928 |  |  | 0,676 | 0,051 | -0,158 | -0,747 | 0,986 | 0,146 | 3,723 | 0,117 |
| WikiB2 | SklearnPlsa | 1,000 | 1,000 | 1,000 | 1,000 |  |  | 0,415 | -0,007 | -0,438 | -0,710 | 0,951 | 0,430 | 4,788 | 0,137 |
| WikiB2 | SklearnBatchVbLda | 1,000 | 1,000 | 1,000 | 1,000 |  | 489,830 | 0,532 | 0,010 | -0,597 | -0,924 | 0,962 | 0,243 | 31,658 | 0,137 |
| WikiB2 | SklearnOnlineVbLda | 0,973 | 0,973 | 0,903 | 0,878 |  | 512,356 | 0,513 | 0,005 | -0,613 | -0,881 | 0,952 | 0,338 | 32,065 | 0,160 |
| WikiB2 | GensimBatchVbLda | 0,990 | 0,990 | 0,966 | 0,958 |  | 485,084 | 0,542 | 0,014 | -0,460 | -0,854 | 0,959 | 0,264 | 31,763 | 0,142 |
| WikiB2 | GensimOnlineVbLda | 0,994 | 0,994 | 0,978 | 0,969 |  | 487,360 | 0,541 | 0,015 | -0,406 | -0,813 | 0,959 | 0,299 | 31,976 | 0,152 |
| WikiB2 | MalletGibbsLda | 1,000 | 1,000 | 1,000 | 1,000 |  | 495,150 | 0,529 | 0,010 | -0,590 | -0,919 | 0,963 | 0,234 | 31,467 | 0,126 |
| WikiB3 | SklearnKmeans | 0,909 | 0,909 | 0,745 | 0,713 |  |  | 0,479 | -0,035 | -1,747 | -1,334 | 0,947 | 0,993 | 30,648 | 0,224 |
| WikiB3 | SklearnLsi | 0,790 | 0,790 | 0,531 | 0,608 |  |  | 0,575 | 0,014 | -1,126 | -1,144 | 0,957 |  |  |  |
| WikiB3 | SklearnNmf | 0,946 | 0,946 | 0,846 | 0,800 |  |  | 0,659 | 0,054 | -0,302 | -0,913 | 0,970 | 0,188 | 2,442 | 0,145 |
| WikiB3 | SklearnPlsa | 0,919 | 0,919 | 0,777 | 0,733 |  |  | 0,387 | -0,007 | -0,395 | -0,748 | 0,952 | 0,443 | 2,817 | 0,163 |
| WikiB3 | SklearnBatchVbLda | 0,867 | 0,881 | 0,736 | 0,734 |  | 417,118 | 0,516 | 0,006 | -0,720 | -0,964 | 0,948 | 0,312 | 33,290 | 0,164 |
| WikiB3 | SklearnOnlineVbLda | 0,859 | 0,865 | 0,704 | 0,694 |  | 427,291 | 0,471 | -0,005 | -0,827 | -1,058 | 0,939 | 0,377 | 33,956 | 0,182 |
| WikiB3 | GensimBatchVbLda | 0,854 | 0,864 | 0,700 | 0,699 |  | 412,686 | 0,487 | -0,002 | -0,825 | -1,082 | 0,942 | 0,352 | 33,497 | 0,175 |
| WikiB3 | GensimOnlineVbLda | 0,852 | 0,858 | 0,691 | 0,690 |  | 421,370 | 0,465 | -0,004 | -0,767 | -0,963 | 0,939 | 0,368 | 33,731 | 0,180 |
| WikiB3 | MalletGibbsLda | 0,934 | 0,934 | 0,817 | 0,789 |  | 467,575 | 0,524 | 0,011 | -0,580 | -0,902 | 0,949 | 0,314 | 32,897 | 0,159 |
| WikiB5 | SklearnKmeans | 0,875 | 0,875 | 0,700 | 0,721 |  |  | 0,528 | 0,009 | -0,919 | -1,730 | 0,886 | 0,994 | 38,443 | 0,250 |
| WikiB5 | SklearnLsi | 0,848 | 0,848 | 0,628 | 0,681 |  |  | 0,612 | 0,022 | -1,053 | -1,362 | 0,901 |  |  |  |
| WikiB5 | SklearnNmf | 0,880 | 0,880 | 0,714 | 0,718 |  |  | 0,716 | 0,088 | 0,262 | -0,941 | 0,937 | 0,160 | 2,124 | 0,147 |
| WikiB5 | SklearnPlsa | 0,858 | 0,858 | 0,681 | 0,668 |  |  | 0,467 | 0,009 | -0,397 | -0,908 | 0,904 | 0,408 | 1,854 | 0,172 |
| WikiB5 | SklearnBatchVbLda | 0,766 | 0,800 | 0,663 | 0,704 |  | 358,426 | 0,557 | 0,047 | 0,068 | -0,914 | 0,902 | 0,259 | 36,290 | 0,165 |
| WikiB5 | SklearnOnlineVbLda | 0,731 | 0,764 | 0,627 | 0,680 |  | 365,329 | 0,540 | 0,038 | -0,136 | -0,989 | 0,897 | 0,286 | 37,062 | 0,176 |
| WikiB5 | GensimBatchVbLda | 0,805 | 0,813 | 0,665 | 0,692 |  | 354,552 | 0,548 | 0,045 | 0,073 | -0,900 | 0,898 | 0,301 | 36,896 | 0,180 |
| WikiB5 | GensimOnlineVbLda | 0,773 | 0,795 | 0,665 | 0,706 |  | 355,391 | 0,550 | 0,044 | -0,013 | -0,949 | 0,900 | 0,283 | 36,691 | 0,174 |
| WikiB5 | MalletGibbsLda | 0,879 | 0,881 | 0,754 | 0,753 |  | 436,247 | 0,575 | 0,051 | 0,142 | -0,878 | 0,903 | 0,262 | 36,056 | 0,164 |
| WikiB7 | SklearnKmeans | 0,891 | 0,894 | 0,760 | 0,801 |  |  | 0,528 | 0,010 | -0,962 | -1,633 | 0,881 | 0,995 | 48,522 | 0,270 |
| WikiB7 | SklearnLsi | 0,851 | 0,851 | 0,649 | 0,737 |  |  | 0,635 | 0,032 | -1,196 | -1,692 | 0,905 |  |  |  |
| WikiB7 | SklearnNmf | 0,906 | 0,906 | 0,788 | 0,816 |  |  | 0,743 | 0,096 | 0,168 | -0,994 | 0,935 | 0,115 | 2,819 | 0,152 |
| WikiB7 | SklearnPlsa | 0,849 | 0,849 | 0,693 | 0,714 |  |  | 0,425 | 0,002 | -0,455 | -1,062 | 0,892 | 0,352 | 2,060 | 0,171 |
| WikiB7 | SklearnBatchVbLda | 0,801 | 0,820 | 0,728 | 0,788 |  | 306,421 | 0,575 | 0,054 | 0,083 | -1,062 | 0,891 | 0,197 | 42,071 | 0,163 |
| WikiB7 | SklearnOnlineVbLda | 0,731 | 0,752 | 0,655 | 0,734 |  | 314,606 | 0,551 | 0,047 | -0,033 | -1,079 | 0,888 | 0,235 | 43,296 | 0,177 |
| WikiB7 | GensimBatchVbLda | 0,796 | 0,811 | 0,716 | 0,774 |  | 301,898 | 0,556 | 0,049 | 0,043 | -1,044 | 0,887 | 0,232 | 43,231 | 0,180 |
| WikiB7 | GensimOnlineVbLda | 0,785 | 0,808 | 0,707 | 0,771 |  | 303,040 | 0,574 | 0,055 | 0,111 | -1,036 | 0,891 | 0,217 | 42,699 | 0,170 |
| WikiB7 | MalletGibbsLda | 0,902 | 0,908 | 0,821 | 0,846 |  | 380,324 | 0,590 | 0,063 | 0,295 | -0,995 | 0,894 | 0,202 | 41,611 | 0,165 |
| WikiB10 | SklearnKmeans | 0,784 | 0,814 | 0,624 | 0,757 |  |  | 0,530 | 0,017 | -0,840 | -1,736 | 0,839 | 0,995 | 60,436 | 0,285 |
| WikiB10 | SklearnLsi | 0,831 | 0,831 | 0,637 | 0,763 |  |  | 0,593 | 0,010 | -1,619 | -2,097 | 0,849 |  |  |  |
| WikiB10 | SklearnNmf | 0,864 | 0,864 | 0,746 | 0,793 |  |  | 0,709 | 0,093 | 0,268 | -1,108 | 0,898 | 0,105 | 1,276 | 0,154 |
| WikiB10 | SklearnPlsa | 0,809 | 0,809 | 0,648 | 0,697 |  |  | 0,417 | 0,000 | -0,541 | -1,237 | 0,846 | 0,327 | 1,630 | 0,171 |
| WikiB10 | SklearnBatchVbLda | 0,744 | 0,765 | 0,652 | 0,746 |  | 297,488 | 0,569 | 0,053 | 0,016 | -1,160 | 0,857 | 0,182 | 46,949 | 0,164 |
| WikiB10 | SklearnOnlineVbLda | 0,648 | 0,678 | 0,576 | 0,693 |  | 306,183 | 0,550 | 0,047 | -0,097 | -1,199 | 0,853 | 0,207 | 48,391 | 0,173 |
| WikiB10 | GensimBatchVbLda | 0,690 | 0,717 | 0,609 | 0,716 |  | 297,016 | 0,542 | 0,044 | -0,133 | -1,217 | 0,851 | 0,223 | 48,653 | 0,184 |
| WikiB10 | GensimOnlineVbLda | 0,670 | 0,697 | 0,596 | 0,711 |  | 296,722 | 0,562 | 0,053 | -0,006 | -1,197 | 0,858 | 0,191 | 47,607 | 0,166 |
| WikiB10 | MalletGibbsLda | 0,794 | 0,808 | 0,692 | 0,768 |  | 395,657 | 0,585 | 0,062 | 0,232 | -1,111 | 0,860 | 0,191 | 47,016 | 0,168 |
| WikiB13 | SklearnKmeans | 0,737 | 0,760 | 0,572 | 0,721 |  |  | 0,504 | 0,021 | -0,448 | -1,431 | 0,813 | 0,996 | 71,710 | 0,300 |
| WikiB13 | SklearnLsi | 0,625 | 0,630 | 0,454 | 0,625 |  |  | 0,451 | -0,057 | -2,725 | -2,070 | 0,802 |  |  |  |
| WikiB13 | SklearnNmf | 0,747 | 0,790 | 0,622 | 0,744 |  |  | 0,667 | 0,069 | -0,005 | -1,226 | 0,882 | 0,099 | 7,194 | 0,153 |
| WikiB13 | SklearnPlsa | 0,648 | 0,652 | 0,460 | 0,577 |  |  | 0,448 | 0,006 | -0,417 | -1,242 | 0,831 | 0,232 | 4,337 | 0,154 |
| WikiB13 | SklearnBatchVbLda | 0,642 | 0,678 | 0,559 | 0,696 |  | 299,544 | 0,575 | 0,055 | 0,083 | -1,212 | 0,857 | 0,173 | 49,771 | 0,160 |
| WikiB13 | SklearnOnlineVbLda | 0,505 | 0,541 | 0,428 | 0,604 |  | 313,709 | 0,546 | 0,045 | -0,069 | -1,249 | 0,853 | 0,193 | 51,252 | 0,166 |
| WikiB13 | GensimBatchVbLda | 0,617 | 0,654 | 0,526 | 0,673 |  | 300,355 | 0,553 | 0,048 | 0,033 | -1,204 | 0,847 | 0,221 | 52,271 | 0,185 |
| WikiB13 | GensimOnlineVbLda | 0,575 | 0,610 | 0,486 | 0,647 |  | 303,250 | 0,568 | 0,051 | 0,049 | -1,231 | 0,858 | 0,175 | 50,149 | 0,157 |
| WikiB13 | MalletGibbsLda | 0,746 | 0,774 | 0,646 | 0,749 |  | 421,943 | 0,588 | 0,062 | 0,292 | -1,164 | 0,855 | 0,198 | 50,143 | 0,170 |
| WikiB15 | SklearnKmeans | 0,717 | 0,743 | 0,528 | 0,712 |  |  | 0,490 | 0,018 | -0,538 | -1,271 | 0,799 | 0,995 | 71,072 | 0,303 |
| WikiB15 | SklearnLsi | 0,584 | 0,607 | 0,398 | 0,600 |  |  | 0,398 | -0,081 | -3,245 | -2,172 | 0,770 |  |  |  |
| WikiB15 | SklearnNmf | 0,724 | 0,770 | 0,620 | 0,741 |  |  | 0,643 | 0,066 | 0,062 | -1,204 | 0,862 | 0,089 | 6,852 | 0,154 |
| WikiB15 | SklearnPlsa | 0,570 | 0,596 | 0,413 | 0,541 |  |  | 0,443 | 0,007 | -0,461 | -1,291 | 0,819 | 0,204 | 7,027 | 0,148 |
| WikiB15 | SklearnBatchVbLda | 0,654 | 0,686 | 0,570 | 0,703 |  | 307,476 | 0,563 | 0,053 | 0,150 | -1,205 | 0,832 | 0,176 | 49,776 | 0,162 |
| WikiB15 | SklearnOnlineVbLda | 0,517 | 0,554 | 0,444 | 0,616 |  | 322,418 | 0,539 | 0,045 | -0,025 | -1,235 | 0,830 | 0,169 | 50,329 | 0,160 |
| WikiB15 | GensimBatchVbLda | 0,608 | 0,643 | 0,517 | 0,669 |  | 310,009 | 0,533 | 0,043 | -0,023 | -1,220 | 0,821 | 0,217 | 52,754 | 0,186 |
| WikiB15 | GensimOnlineVbLda | 0,512 | 0,553 | 0,446 | 0,619 |  | 314,700 | 0,548 | 0,048 | 0,027 | -1,238 | 0,836 | 0,153 | 49,873 | 0,150 |
| WikiB15 | MalletGibbsLda | 0,738 | 0,763 | 0,631 | 0,739 |  | 455,198 | 0,570 | 0,059 | 0,301 | -1,164 | 0,831 | 0,189 | 49,976 | 0,170 |
| WikiB17 | SklearnKmeans | 0,693 | 0,719 | 0,510 | 0,693 |  |  | 0,495 | 0,026 | -0,323 | -1,189 | 0,805 | 0,996 | 77,061 | 0,308 |
| WikiB17 | SklearnLsi | 0,627 | 0,631 | 0,411 | 0,596 |  |  | 0,420 | -0,061 | -2,652 | -2,099 | 0,779 |  |  |  |
| WikiB17 | SklearnNmf | 0,769 | 0,772 | 0,576 | 0,728 |  |  | 0,631 | 0,059 | -0,057 | -1,306 | 0,858 | 0,088 | 14,923 | 0,149 |
| WikiB17 | SklearnPlsa | 0,572 | 0,580 | 0,390 | 0,508 |  |  | 0,442 | 0,010 | -0,373 | -1,281 | 0,830 | 0,185 | 11,109 | 0,145 |
| WikiB17 | SklearnBatchVbLda | 0,608 | 0,641 | 0,507 | 0,660 |  | 320,266 | 0,548 | 0,050 | 0,095 | -1,234 | 0,833 | 0,167 | 51,144 | 0,159 |
| WikiB17 | SklearnOnlineVbLda | 0,437 | 0,476 | 0,346 | 0,553 |  | 337,143 | 0,518 | 0,040 | -0,110 | -1,271 | 0,831 | 0,154 | 52,263 | 0,154 |
| WikiB17 | GensimBatchVbLda | 0,598 | 0,624 | 0,491 | 0,651 |  | 321,274 | 0,530 | 0,044 | 0,008 | -1,240 | 0,824 | 0,211 | 54,127 | 0,185 |
| WikiB17 | GensimOnlineVbLda | 0,472 | 0,513 | 0,378 | 0,586 |  | 326,435 | 0,535 | 0,046 | -0,011 | -1,259 | 0,836 | 0,138 | 50,794 | 0,144 |
| WikiB17 | MalletGibbsLda | 0,701 | 0,731 | 0,587 | 0,711 |  | 492,180 | 0,562 | 0,058 | 0,273 | -1,202 | 0,834 | 0,180 | 51,638 | 0,167 |
| WikiB20 | SklearnKmeans | 0,679 | 0,706 | 0,475 | 0,685 |  |  | 0,477 | 0,022 | -0,307 | -1,180 | 0,770 | 0,996 | 84,483 | 0,315 |
| WikiB20 | SklearnLsi | 0,497 | 0,528 | 0,330 | 0,518 |  |  | 0,328 | -0,098 | -3,255 | -2,180 | 0,718 |  |  |  |
| WikiB20 | SklearnNmf | 0,699 | 0,739 | 0,565 | 0,722 |  |  | 0,619 | 0,060 | 0,058 | -1,262 | 0,835 | 0,079 | 19,744 | 0,150 |
| WikiB20 | SklearnPlsa | 0,508 | 0,515 | 0,344 | 0,478 |  |  | 0,455 | 0,006 | -0,496 | -1,317 | 0,807 | 0,142 | 23,294 | 0,136 |
| WikiB20 | SklearnBatchVbLda | 0,605 | 0,635 | 0,506 | 0,669 |  | 323,476 | 0,547 | 0,052 | 0,182 | -1,216 | 0,811 | 0,159 | 52,023 | 0,159 |
| WikiB20 | SklearnOnlineVbLda | 0,427 | 0,465 | 0,355 | 0,557 |  | 340,188 | 0,529 | 0,044 | 0,018 | -1,244 | 0,812 | 0,144 | 52,424 | 0,152 |
| WikiB20 | GensimBatchVbLda | 0,574 | 0,602 | 0,489 | 0,654 |  | 325,387 | 0,527 | 0,046 | 0,118 | -1,200 | 0,801 | 0,209 | 56,228 | 0,187 |
| WikiB20 | GensimOnlineVbLda | 0,464 | 0,500 | 0,381 | 0,584 |  | 328,969 | 0,546 | 0,051 | 0,124 | -1,241 | 0,818 | 0,127 | 50,835 | 0,142 |
| WikiB20 | MalletGibbsLda | 0,676 | 0,704 | 0,556 | 0,697 |  | 513,498 | 0,561 | 0,060 | 0,383 | -1,177 | 0,811 | 0,179 | 52,872 | 0,168 |
| WikiB23 | SklearnKmeans | 0,607 | 0,643 | 0,426 | 0,631 |  |  | 0,457 | 0,022 | -0,252 | -1,174 | 0,756 | 0,996 | 87,875 | 0,318 |
| WikiB23 | SklearnLsi | 0,520 | 0,541 | 0,342 | 0,511 |  |  | 0,334 | -0,084 | -2,777 | -1,817 | 0,707 |  |  |  |
| WikiB23 | SklearnNmf | 0,682 | 0,705 | 0,517 | 0,656 |  |  | 0,591 | 0,056 | 0,092 | -1,298 | 0,814 | 0,076 | 25,815 | 0,148 |
| WikiB23 | SklearnPlsa | 0,483 | 0,490 | 0,313 | 0,458 |  |  | 0,448 | 0,008 | -0,450 | -1,355 | 0,797 | 0,144 | 28,915 | 0,133 |
| WikiB23 | SklearnBatchVbLda | 0,557 | 0,581 | 0,449 | 0,624 |  | 336,552 | 0,535 | 0,052 | 0,234 | -1,206 | 0,796 | 0,143 | 52,442 | 0,154 |
| WikiB23 | SklearnOnlineVbLda | 0,383 | 0,417 | 0,298 | 0,522 |  | 354,412 | 0,507 | 0,038 | -0,066 | -1,262 | 0,795 | 0,116 | 52,602 | 0,142 |
| WikiB23 | GensimBatchVbLda | 0,525 | 0,558 | 0,433 | 0,614 |  | 339,764 | 0,515 | 0,045 | 0,131 | -1,201 | 0,786 | 0,187 | 57,038 | 0,184 |
| WikiB23 | GensimOnlineVbLda | 0,405 | 0,436 | 0,317 | 0,539 |  | 345,125 | 0,520 | 0,043 | 0,023 | -1,257 | 0,801 | 0,105 | 50,685 | 0,134 |
| WikiB23 | MalletGibbsLda | 0,640 | 0,663 | 0,518 | 0,663 |  | 567,695 | 0,548 | 0,060 | 0,398 | -1,153 | 0,797 | 0,165 | 53,528 | 0,165 |
| WikiB25 | SklearnKmeans | 0,596 | 0,623 | 0,408 | 0,610 |  |  | 0,438 | 0,017 | -0,294 | -1,240 | 0,736 | 0,997 | 93,313 | 0,323 |
| WikiB25 | SklearnLsi | 0,422 | 0,446 | 0,285 | 0,460 |  |  | 0,313 | -0,076 | -2,520 | -1,866 | 0,700 |  |  |  |
| WikiB25 | SklearnNmf | 0,649 | 0,665 | 0,475 | 0,635 |  |  | 0,566 | 0,050 | 0,012 | -1,339 | 0,803 | 0,074 | 39,452 | 0,147 |
| WikiB25 | SklearnPlsa | 0,394 | 0,410 | 0,280 | 0,422 |  |  | 0,454 | 0,012 | -0,403 | -1,380 | 0,793 | 0,115 | 45,174 | 0,125 |
| WikiB25 | SklearnBatchVbLda | 0,537 | 0,566 | 0,430 | 0,613 |  | 326,077 | 0,526 | 0,050 | 0,205 | -1,251 | 0,787 | 0,135 | 52,951 | 0,153 |
| WikiB25 | SklearnOnlineVbLda | 0,382 | 0,410 | 0,291 | 0,517 |  | 343,833 | 0,502 | 0,037 | -0,072 | -1,300 | 0,789 | 0,106 | 52,492 | 0,140 |
| WikiB25 | GensimBatchVbLda | 0,513 | 0,543 | 0,415 | 0,602 |  | 329,353 | 0,505 | 0,043 | 0,120 | -1,246 | 0,776 | 0,180 | 57,820 | 0,182 |
| WikiB25 | GensimOnlineVbLda | 0,417 | 0,444 | 0,324 | 0,541 |  | 332,982 | 0,513 | 0,043 | 0,035 | -1,301 | 0,795 | 0,097 | 50,979 | 0,132 |
| WikiB25 | MalletGibbsLda | 0,622 | 0,646 | 0,491 | 0,649 |  | 549,421 | 0,540 | 0,058 | 0,375 | -1,205 | 0,787 | 0,159 | 54,043 | 0,163 |
| WikiB27 | SklearnKmeans | 0,577 | 0,600 | 0,390 | 0,598 |  |  | 0,458 | 0,020 | -0,294 | -1,219 | 0,741 | 0,997 | 98,069 | 0,324 |
| WikiB27 | SklearnLsi | 0,409 | 0,438 | 0,251 | 0,438 |  |  | 0,300 | -0,087 | -2,790 | -1,993 | 0,687 |  |  |  |
| WikiB27 | SklearnNmf | 0,552 | 0,584 | 0,410 | 0,595 |  |  | 0,568 | 0,048 | -0,075 | -1,403 | 0,804 | 0,067 | 39,856 | 0,144 |
| WikiB27 | SklearnPlsa | 0,340 | 0,359 | 0,248 | 0,392 |  |  | 0,441 | 0,006 | -0,498 | -1,383 | 0,798 | 0,091 | 63,812 | 0,120 |
| WikiB27 | SklearnBatchVbLda | 0,523 | 0,554 | 0,413 | 0,606 |  | 334,273 | 0,539 | 0,053 | 0,241 | -1,269 | 0,789 | 0,134 | 53,692 | 0,151 |
| WikiB27 | SklearnOnlineVbLda | 0,370 | 0,397 | 0,285 | 0,506 |  | 351,787 | 0,520 | 0,044 | 0,033 | -1,310 | 0,793 | 0,107 | 53,123 | 0,139 |
| WikiB27 | GensimBatchVbLda | 0,493 | 0,524 | 0,393 | 0,591 |  | 338,272 | 0,518 | 0,045 | 0,118 | -1,271 | 0,779 | 0,174 | 58,504 | 0,180 |
| WikiB27 | GensimOnlineVbLda | 0,374 | 0,400 | 0,289 | 0,514 |  | 342,669 | 0,530 | 0,047 | 0,081 | -1,312 | 0,799 | 0,089 | 49,839 | 0,128 |
| WikiB27 | MalletGibbsLda | 0,602 | 0,631 | 0,472 | 0,642 |  | 568,393 | 0,554 | 0,061 | 0,399 | -1,229 | 0,790 | 0,158 | 55,241 | 0,161 |
| WikiB30 | SklearnKmeans | 0,527 | 0,558 | 0,349 | 0,574 |  |  | 0,471 | 0,022 | -0,289 | -1,287 | 0,735 | 0,997 | 100,257 | 0,325 |
| WikiB30 | SklearnLsi | 0,364 | 0,388 | 0,214 | 0,406 |  |  | 0,302 | -0,083 | -2,677 | -1,964 | 0,674 |  |  |  |
| WikiB30 | SklearnNmf | 0,548 | 0,575 | 0,404 | 0,585 |  |  | 0,572 | 0,054 | 0,095 | -1,369 | 0,796 | 0,061 | 50,167 | 0,141 |
| WikiB30 | SklearnPlsa | 0,315 | 0,330 | 0,223 | 0,363 |  |  | 0,452 | 0,005 | -0,579 | -1,454 | 0,781 | 0,067 | 104,613 | 0,113 |
| WikiB30 | SklearnBatchVbLda | 0,498 | 0,531 | 0,390 | 0,586 |  | 327,482 | 0,539 | 0,053 | 0,258 | -1,275 | 0,777 | 0,119 | 51,842 | 0,148 |
| WikiB30 | SklearnOnlineVbLda | 0,333 | 0,363 | 0,245 | 0,489 |  | 345,752 | 0,513 | 0,040 | 0,001 | -1,306 | 0,779 | 0,088 | 52,807 | 0,133 |
| WikiB30 | GensimBatchVbLda | 0,472 | 0,500 | 0,368 | 0,573 |  | 332,693 | 0,512 | 0,043 | 0,111 | -1,272 | 0,764 | 0,160 | 57,809 | 0,178 |
| WikiB30 | GensimOnlineVbLda | 0,366 | 0,393 | 0,271 | 0,508 |  | 334,920 | 0,523 | 0,044 | 0,046 | -1,303 | 0,784 | 0,078 | 49,346 | 0,125 |
| WikiB30 | MalletGibbsLda | 0,578 | 0,604 | 0,441 | 0,618 |  | 571,132 | 0,550 | 0,060 | 0,395 | -1,230 | 0,776 | 0,144 | 54,179 | 0,159 |
| WikiB35 | SklearnKmeans | 0,476 | 0,497 | 0,302 | 0,532 |  |  | 0,455 | 0,026 | -0,090 | -1,187 | 0,695 | 0,997 | 107,316 | 0,328 |
| WikiB35 | SklearnLsi | 0,288 | 0,331 | 0,146 | 0,329 |  |  | 0,278 | -0,074 | -2,302 | -1,891 | 0,645 |  |  |  |
| WikiB35 | SklearnNmf | 0,480 | 0,496 | 0,324 | 0,523 |  |  | 0,556 | 0,052 | 0,088 | -1,419 | 0,769 | 0,055 | 91,492 | 0,138 |
| WikiB35 | SklearnPlsa | 0,276 | 0,286 | 0,193 | 0,339 |  |  | 0,441 | 0,004 | -0,596 | -1,476 | 0,764 | 0,048 | 182,547 | 0,105 |
| WikiB35 | SklearnBatchVbLda | 0,447 | 0,477 | 0,341 | 0,556 |  | 333,968 | 0,525 | 0,054 | 0,326 | -1,256 | 0,749 | 0,112 | 53,206 | 0,146 |
| WikiB35 | SklearnOnlineVbLda | 0,290 | 0,316 | 0,215 | 0,458 |  | 355,531 | 0,497 | 0,039 | 0,020 | -1,315 | 0,752 | 0,079 | 52,780 | 0,128 |
| WikiB35 | GensimBatchVbLda | 0,435 | 0,463 | 0,336 | 0,552 |  | 339,338 | 0,503 | 0,046 | 0,208 | -1,250 | 0,736 | 0,156 | 60,031 | 0,177 |
| WikiB35 | GensimOnlineVbLda | 0,314 | 0,338 | 0,232 | 0,472 |  | 343,386 | 0,503 | 0,041 | 0,040 | -1,320 | 0,758 | 0,068 | 49,821 | 0,119 |
| WikiB35 | MalletGibbsLda | 0,525 | 0,551 | 0,390 | 0,589 |  | 603,717 | 0,540 | 0,062 | 0,481 | -1,205 | 0,749 | 0,135 | 55,875 | 0,156 |
| WikiB40 | SklearnKmeans | 0,474 | 0,499 | 0,302 | 0,535 |  |  | 0,441 | 0,022 | -0,124 | -1,253 | 0,689 | 0,997 | 114,651 | 0,334 |
| WikiB40 | SklearnLsi | 0,302 | 0,322 | 0,180 | 0,342 |  |  | 0,268 | -0,069 | -2,014 | -1,900 | 0,630 |  |  |  |
| WikiB40 | SklearnNmf | 0,460 | 0,483 | 0,302 | 0,502 |  |  | 0,541 | 0,050 | 0,148 | -1,407 | 0,760 | 0,049 | 136,430 | 0,136 |
| WikiB40 | SklearnPlsa | 0,237 | 0,247 | 0,155 | 0,293 |  |  | 0,437 | 0,001 | -0,690 | -1,590 | 0,771 | 0,029 | 296,340 | 0,098 |
| WikiB40 | SklearnBatchVbLda | 0,455 | 0,481 | 0,350 | 0,562 |  | 319,360 | 0,510 | 0,049 | 0,289 | -1,286 | 0,740 | 0,100 | 51,953 | 0,143 |
| WikiB40 | SklearnOnlineVbLda | 0,281 | 0,301 | 0,202 | 0,453 |  | 343,271 | 0,484 | 0,035 | -0,001 | -1,352 | 0,745 | 0,080 | 63,956 | 0,128 |
| WikiB40 | GensimBatchVbLda | 0,432 | 0,458 | 0,332 | 0,553 |  | 325,348 | 0,490 | 0,042 | 0,203 | -1,278 | 0,727 | 0,140 | 58,773 | 0,174 |
| WikiB40 | GensimOnlineVbLda | 0,299 | 0,320 | 0,223 | 0,470 |  | 329,495 | 0,483 | 0,034 | -0,040 | -1,358 | 0,749 | 0,058 | 53,709 | 0,115 |
| WikiB40 | MalletGibbsLda | 0,532 | 0,557 | 0,396 | 0,597 |  | 585,082 | 0,520 | 0,055 | 0,408 | -1,247 | 0,735 | 0,130 | 55,994 | 0,156 |
| WikiB45 | SklearnKmeans | 0,476 | 0,501 | 0,296 | 0,534 |  |  | 0,445 | 0,027 | 0,009 | -1,222 | 0,670 | 0,997 | 120,378 | 0,334 |
| WikiB45 | SklearnLsi | 0,295 | 0,318 | 0,171 | 0,341 |  |  | 0,249 | -0,083 | -2,338 | -1,967 | 0,610 |  |  |  |
| WikiB45 | SklearnNmf | 0,454 | 0,479 | 0,289 | 0,500 |  |  | 0,526 | 0,046 | 0,118 | -1,427 | 0,738 | 0,043 | 190,445 | 0,134 |
| WikiB45 | SklearnPlsa | 0,202 | 0,215 | 0,127 | 0,265 |  |  | 0,434 | 0,003 | -0,615 | -1,573 | 0,756 | 0,022 | 406,726 | 0,095 |
| WikiB45 | SklearnBatchVbLda | 0,432 | 0,458 | 0,332 | 0,551 |  | 319,163 | 0,500 | 0,047 | 0,277 | -1,291 | 0,720 | 0,088 | 50,089 | 0,140 |
| WikiB45 | SklearnOnlineVbLda | 0,256 | 0,274 | 0,203 | 0,451 |  | 344,189 | 0,472 | 0,033 | -0,030 | -1,359 | 0,726 | 0,076 | 66,350 | 0,125 |
| WikiB45 | GensimBatchVbLda | 0,409 | 0,435 | 0,314 | 0,539 |  | 326,571 | 0,484 | 0,042 | 0,208 | -1,293 | 0,708 | 0,128 | 58,855 | 0,171 |
| WikiB45 | GensimOnlineVbLda | 0,277 | 0,298 | 0,209 | 0,455 |  | 330,130 | 0,475 | 0,030 | -0,110 | -1,384 | 0,735 | 0,048 | 51,419 | 0,111 |
| WikiB45 | MalletGibbsLda | 0,500 | 0,523 | 0,369 | 0,576 |  | 599,669 | 0,518 | 0,056 | 0,445 | -1,237 | 0,718 | 0,112 | 54,224 | 0,152 |
| WikiB50 | SklearnKmeans | 0,438 | 0,462 | 0,274 | 0,513 |  |  | 0,428 | 0,022 | -0,061 | -1,187 | 0,649 | 0,997 | 123,009 | 0,335 |
| WikiB50 | SklearnLsi | 0,276 | 0,289 | 0,168 | 0,331 |  |  | 0,251 | -0,076 | -2,145 | -1,912 | 0,601 |  |  |  |
| WikiB50 | SklearnNmf | 0,392 | 0,408 | 0,240 | 0,453 |  |  | 0,516 | 0,047 | 0,185 | -1,448 | 0,730 | 0,038 | 255,428 | 0,131 |
| WikiB50 | SklearnPlsa | 0,182 | 0,190 | 0,126 | 0,257 |  |  | 0,427 | -0,002 | -0,758 | -1,625 | 0,750 | 0,016 | 565,783 | 0,092 |
| WikiB50 | SklearnBatchVbLda | 0,412 | 0,436 | 0,303 | 0,533 |  | 326,084 | 0,496 | 0,049 | 0,322 | -1,280 | 0,711 | 0,083 | 48,683 | 0,138 |
| WikiB50 | SklearnOnlineVbLda | 0,244 | 0,260 | 0,177 | 0,431 |  | 351,462 | 0,468 | 0,033 | 0,018 | -1,345 | 0,714 | 0,079 | 75,987 | 0,125 |
| WikiB50 | GensimBatchVbLda | 0,396 | 0,422 | 0,303 | 0,531 |  | 332,882 | 0,481 | 0,043 | 0,260 | -1,262 | 0,697 | 0,124 | 57,752 | 0,171 |
| WikiB50 | GensimOnlineVbLda | 0,252 | 0,273 | 0,188 | 0,440 |  | 335,521 | 0,465 | 0,027 | -0,152 | -1,386 | 0,722 | 0,048 | 64,797 | 0,110 |
| WikiB50 | MalletGibbsLda | 0,486 | 0,508 | 0,342 | 0,558 |  | 624,129 | 0,515 | 0,057 | 0,468 | -1,230 | 0,707 | 0,113 | 53,947 | 0,151 |
| WikiB55 | SklearnKmeans | 0,406 | 0,429 | 0,245 | 0,483 |  |  | 0,427 | 0,024 | 0,024 | -1,171 | 0,638 | 0,997 | 128,033 | 0,339 |
| WikiB55 | SklearnLsi | 0,230 | 0,244 | 0,129 | 0,286 |  |  | 0,252 | -0,064 | -1,781 | -1,888 | 0,590 |  |  |  |
| WikiB55 | SklearnNmf | 0,367 | 0,385 | 0,211 | 0,421 |  |  | 0,499 | 0,039 | 0,079 | -1,465 | 0,719 | 0,034 | 39,768 | 0,128 |
| WikiB55 | SklearnPlsa | 0,368 | 0,385 | 0,286 | 0,487 |  |  | 0,497 | 0,040 | 0,081 | -1,375 | 0,733 | 0,026 | 385,455 | 0,109 |
| WikiB55 | SklearnBatchVbLda | 0,379 | 0,405 | 0,274 | 0,507 |  | 316,246 | 0,485 | 0,045 | 0,291 | -1,290 | 0,700 | 0,075 | 47,222 | 0,135 |
| WikiB55 | SklearnOnlineVbLda | 0,218 | 0,235 | 0,165 | 0,417 |  | 342,818 | 0,448 | 0,025 | -0,094 | -1,382 | 0,705 | 0,076 | 100,079 | 0,122 |
| WikiB55 | GensimBatchVbLda | 0,373 | 0,397 | 0,278 | 0,513 |  | 321,921 | 0,472 | 0,041 | 0,239 | -1,267 | 0,687 | 0,115 | 56,679 | 0,169 |
| WikiB55 | GensimOnlineVbLda | 0,236 | 0,253 | 0,169 | 0,422 |  | 325,241 | 0,449 | 0,022 | -0,210 | -1,409 | 0,713 | 0,048 | 91,484 | 0,108 |
| WikiB55 | MalletGibbsLda | 0,458 | 0,479 | 0,317 | 0,539 |  | 621,914 | 0,503 | 0,053 | 0,416 | -1,267 | 0,698 | 0,107 | 53,542 | 0,149 |
| WikiB60 | SklearnKmeans | 0,388 | 0,418 | 0,233 | 0,468 |  |  | 0,432 | 0,024 | -0,007 | -1,179 | 0,629 | 0,997 | 131,087 | 0,338 |
| WikiB60 | SklearnLsi | 0,238 | 0,256 | 0,135 | 0,288 |  |  | 0,243 | -0,073 | -1,978 | -1,898 | 0,576 |  |  |  |
| WikiB60 | SklearnNmf | 0,346 | 0,369 | 0,200 | 0,411 |  |  | 0,503 | 0,043 | 0,157 | -1,472 | 0,708 | 0,031 | 437,366 | 0,126 |
| WikiB60 | SklearnPlsa | 0,155 | 0,166 | 0,090 | 0,213 |  |  | 0,414 | -0,002 | -0,698 | -1,654 | 0,726 | 0,010 | 827,403 | 0,089 |
| WikiB60 | SklearnBatchVbLda | 0,367 | 0,391 | 0,260 | 0,494 |  | 322,871 | 0,485 | 0,047 | 0,324 | -1,286 | 0,690 | 0,069 | 45,933 | 0,133 |
| WikiB60 | SklearnOnlineVbLda | 0,208 | 0,221 | 0,152 | 0,404 |  | 351,025 | 0,445 | 0,026 | -0,070 | -1,382 | 0,692 | 0,089 | 119,462 | 0,126 |
| WikiB60 | GensimBatchVbLda | 0,358 | 0,380 | 0,259 | 0,496 |  | 329,513 | 0,471 | 0,042 | 0,265 | -1,275 | 0,675 | 0,108 | 57,236 | 0,167 |
| WikiB60 | GensimOnlineVbLda | 0,224 | 0,238 | 0,159 | 0,409 |  | 331,220 | 0,446 | 0,020 | -0,258 | -1,436 | 0,703 | 0,048 | 113,525 | 0,108 |
| WikiB60 | MalletGibbsLda | 0,438 | 0,460 | 0,298 | 0,524 |  | 633,138 | 0,506 | 0,055 | 0,447 | -1,265 | 0,689 | 0,099 | 53,432 | 0,148 |
| WikiB65 | SklearnKmeans | 0,360 | 0,386 | 0,210 | 0,445 |  |  | 0,421 | 0,020 | -0,073 | -1,222 | 0,616 | 0,997 | 137,372 | 0,341 |
| WikiB65 | SklearnLsi | 0,207 | 0,223 | 0,116 | 0,269 |  |  | 0,242 | -0,065 | -1,733 | -1,863 | 0,568 |  |  |  |
| WikiB65 | SklearnNmf | 0,299 | 0,319 | 0,165 | 0,381 |  |  | 0,482 | 0,038 | 0,106 | -1,460 | 0,696 | 0,027 | 38,749 | 0,124 |
| WikiB65 | SklearnPlsa | 0,141 | 0,149 | 0,077 | 0,192 |  |  | 0,404 | -0,011 | -0,901 | -1,698 | 0,723 | 0,007 | 1066,487 | 0,088 |
| WikiB65 | SklearnBatchVbLda | 0,348 | 0,369 | 0,243 | 0,478 |  | 316,924 | 0,478 | 0,045 | 0,327 | -1,287 | 0,681 | 0,063 | 43,573 | 0,131 |
| WikiB65 | SklearnOnlineVbLda | 0,201 | 0,215 | 0,143 | 0,395 |  | 344,789 | 0,442 | 0,026 | -0,031 | -1,378 | 0,684 | 0,070 | 111,191 | 0,119 |
| WikiB65 | GensimBatchVbLda | 0,331 | 0,354 | 0,242 | 0,481 |  | 323,000 | 0,462 | 0,040 | 0,265 | -1,263 | 0,663 | 0,101 | 55,230 | 0,165 |
| WikiB65 | GensimOnlineVbLda | 0,210 | 0,226 | 0,148 | 0,398 |  | 325,871 | 0,435 | 0,020 | -0,192 | -1,431 | 0,695 | 0,043 | 95,066 | 0,106 |
| WikiB65 | MalletGibbsLda | 0,412 | 0,431 | 0,277 | 0,504 |  | 643,577 | 0,494 | 0,051 | 0,428 | -1,245 | 0,677 | 0,093 | 50,976 | 0,146 |
| WikiB70 | SklearnKmeans | 0,340 | 0,366 | 0,193 | 0,430 |  |  | 0,410 | 0,018 | -0,087 | -1,219 | 0,604 | 0,997 | 144,111 | 0,342 |
| WikiB70 | SklearnLsi | 0,198 | 0,215 | 0,106 | 0,254 |  |  | 0,226 | -0,068 | -1,739 | -1,902 | 0,554 |  |  |  |
| WikiB70 | SklearnNmf | 0,279 | 0,300 | 0,137 | 0,351 |  |  | 0,473 | 0,036 | 0,127 | -1,466 | 0,689 | 0,025 | 37,835 | 0,123 |
| WikiB70 | SklearnPlsa | 0,122 | 0,131 | 0,066 | 0,174 |  |  | 0,392 | -0,015 | -0,958 | -1,734 | 0,719 | 0,005 | 1272,369 | 0,086 |
| WikiB70 | SklearnBatchVbLda | 0,319 | 0,340 | 0,220 | 0,458 |  | 326,781 | 0,469 | 0,042 | 0,301 | -1,298 | 0,675 | 0,058 | 42,324 | 0,129 |
| WikiB70 | SklearnOnlineVbLda | 0,177 | 0,190 | 0,122 | 0,374 |  | 354,575 | 0,431 | 0,022 | -0,087 | -1,395 | 0,677 | 0,079 | 130,414 | 0,122 |
| WikiB70 | GensimBatchVbLda | 0,311 | 0,331 | 0,220 | 0,461 |  | 333,266 | 0,453 | 0,037 | 0,238 | -1,267 | 0,656 | 0,095 | 54,165 | 0,165 |
| WikiB70 | GensimOnlineVbLda | 0,185 | 0,200 | 0,129 | 0,382 |  | 330,993 | 0,426 | 0,019 | -0,158 | -1,444 | 0,690 | 0,043 | 136,483 | 0,105 |
| WikiB70 | MalletGibbsLda | 0,388 | 0,408 | 0,257 | 0,486 |  | 668,787 | 0,490 | 0,050 | 0,426 | -1,255 | 0,671 | 0,090 | 50,495 | 0,145 |
| WikiB75 | SklearnKmeans | 0,336 | 0,364 | 0,186 | 0,425 |  |  | 0,415 | 0,021 | -0,040 | -1,245 | 0,600 | 0,997 | 148,134 | 0,341 |
| WikiB75 | SklearnLsi | 0,188 | 0,203 | 0,084 | 0,227 |  |  | 0,223 | -0,066 | -1,689 | -1,927 | 0,546 |  |  |  |
| WikiB75 | SklearnNmf | 0,271 | 0,289 | 0,139 | 0,350 |  |  | 0,477 | 0,038 | 0,145 | -1,491 | 0,682 | 0,023 | 1048,052 | 0,121 |
| WikiB75 | SklearnPlsa | 0,115 | 0,122 | 0,062 | 0,166 |  |  | 0,388 | -0,013 | -0,890 | -1,747 | 0,707 | 0,005 | 1501,183 | 0,086 |
| WikiB75 | SklearnBatchVbLda | 0,308 | 0,330 | 0,210 | 0,451 |  | 324,502 | 0,469 | 0,043 | 0,310 | -1,316 | 0,667 | 0,053 | 41,717 | 0,126 |
| WikiB75 | SklearnOnlineVbLda | 0,167 | 0,179 | 0,116 | 0,369 |  | 356,458 | 0,427 | 0,022 | -0,086 | -1,421 | 0,668 | 0,092 | 165,094 | 0,126 |
| WikiB75 | GensimBatchVbLda | 0,306 | 0,329 | 0,215 | 0,458 |  | 330,600 | 0,455 | 0,039 | 0,273 | -1,289 | 0,650 | 0,090 | 54,327 | 0,163 |
| WikiB75 | GensimOnlineVbLda | 0,170 | 0,183 | 0,119 | 0,371 |  | 331,658 | 0,409 | 0,011 | -0,338 | -1,514 | 0,675 | 0,051 | 191,870 | 0,107 |
| WikiB75 | MalletGibbsLda | 0,378 | 0,399 | 0,250 | 0,482 |  | 658,051 | 0,492 | 0,052 | 0,447 | -1,273 | 0,664 | 0,086 | 50,818 | 0,142 |
| WikiC2 | SklearnKmeans | 1,000 | 1,000 | 1,000 | 1,000 |  |  | 0,654 | 0,020 | -1,092 | -1,541 | 0,983 | 0,987 | 21,305 | 0,149 |
| WikiC2 | SklearnLsi | 1,000 | 1,000 | 1,000 | 1,000 |  |  | 0,745 | 0,040 | -0,898 | -0,734 | 0,990 |  |  |  |
| WikiC2 | SklearnNmf | 1,000 | 1,000 | 1,000 | 1,000 |  |  | 0,733 | 0,037 | -0,927 | -0,739 | 0,989 | 0,074 | 3,346 | 0,094 |
| WikiC2 | SklearnPlsa | 1,000 | 1,000 | 1,000 | 1,000 |  |  | 0,630 | 0,031 | -0,793 | -0,723 | 0,972 | 0,258 | 4,707 | 0,111 |
| WikiC2 | SklearnBatchVbLda | 0,992 | 0,992 | 0,968 | 0,958 |  | 595,020 | 0,673 | 0,052 | -0,205 | -0,681 | 0,985 | 0,128 | 28,384 | 0,119 |
| WikiC2 | SklearnOnlineVbLda | 0,984 | 0,984 | 0,937 | 0,917 |  | 603,754 | 0,636 | 0,033 | -0,665 | -1,007 | 0,977 | 0,176 | 28,654 | 0,133 |
| WikiC2 | GensimBatchVbLda | 0,966 | 0,966 | 0,881 | 0,859 |  | 594,674 | 0,609 | 0,017 | -1,034 | -1,256 | 0,970 | 0,159 | 28,617 | 0,126 |
| WikiC2 | GensimOnlineVbLda | 0,970 | 0,970 | 0,905 | 0,902 |  | 595,884 | 0,637 | 0,034 | -0,641 | -1,074 | 0,976 | 0,180 | 28,724 | 0,134 |
| WikiC2 | MalletGibbsLda | 0,987 | 0,987 | 0,949 | 0,933 |  | 598,683 | 0,664 | 0,047 | -0,339 | -0,713 | 0,982 | 0,118 | 28,158 | 0,103 |
| WikiC3 | SklearnKmeans | 0,993 | 0,993 | 0,980 | 0,969 |  |  | 0,656 | 0,030 | -1,033 | -1,515 | 0,956 | 0,993 | 28,123 | 0,198 |
| WikiC3 | SklearnLsi | 0,980 | 0,980 | 0,940 | 0,929 |  |  | 0,679 | 0,014 | -1,872 | -1,511 | 0,958 |  |  |  |
| WikiC3 | SklearnNmf | 0,993 | 0,993 | 0,980 | 0,969 |  |  | 0,803 | 0,098 | -0,073 | -0,735 | 0,983 | 0,094 | 2,453 | 0,117 |
| WikiC3 | SklearnPlsa | 0,986 | 0,986 | 0,959 | 0,939 |  |  | 0,558 | -0,002 | -1,346 | -0,899 | 0,958 | 0,314 | 4,112 | 0,135 |
| WikiC3 | SklearnBatchVbLda | 0,968 | 0,968 | 0,925 | 0,929 |  | 452,042 | 0,692 | 0,064 | -0,205 | -0,893 | 0,958 | 0,162 | 31,605 | 0,136 |
| WikiC3 | SklearnOnlineVbLda | 0,852 | 0,889 | 0,800 | 0,844 |  | 484,498 | 0,625 | 0,035 | -0,736 | -1,297 | 0,945 | 0,208 | 32,171 | 0,151 |
| WikiC3 | GensimBatchVbLda | 0,941 | 0,941 | 0,880 | 0,886 |  | 451,578 | 0,663 | 0,050 | -0,525 | -1,077 | 0,951 | 0,181 | 31,798 | 0,142 |
| WikiC3 | GensimOnlineVbLda | 0,863 | 0,878 | 0,801 | 0,838 |  | 471,767 | 0,607 | 0,028 | -0,877 | -1,413 | 0,938 | 0,205 | 32,068 | 0,149 |
| WikiC3 | MalletGibbsLda | 0,987 | 0,987 | 0,963 | 0,959 |  | 462,888 | 0,713 | 0,074 | 0,004 | -0,808 | 0,962 | 0,149 | 31,342 | 0,124 |
| WikiC5 | SklearnKmeans | 0,972 | 0,972 | 0,932 | 0,935 |  |  | 0,681 | 0,047 | -1,080 | -1,721 | 0,925 | 0,992 | 36,152 | 0,236 |
| WikiC5 | SklearnLsi | 0,738 | 0,738 | 0,735 | 0,848 |  |  | 0,701 | 0,037 | -1,600 | -1,817 | 0,917 |  |  |  |
| WikiC5 | SklearnNmf | 0,992 | 0,992 | 0,980 | 0,975 |  |  | 0,815 | 0,117 | 0,092 | -0,880 | 0,956 | 0,088 | 1,322 | 0,137 |
| WikiC5 | SklearnPlsa | 0,955 | 0,955 | 0,892 | 0,888 |  |  | 0,519 | 0,004 | -0,890 | -0,961 | 0,906 | 0,349 | 1,958 | 0,161 |
| WikiC5 | SklearnBatchVbLda | 0,924 | 0,935 | 0,891 | 0,915 |  | 334,816 | 0,708 | 0,081 | -0,167 | -1,103 | 0,927 | 0,139 | 35,431 | 0,148 |
| WikiC5 | SklearnOnlineVbLda | 0,785 | 0,835 | 0,753 | 0,833 |  | 356,157 | 0,667 | 0,064 | -0,471 | -1,290 | 0,915 | 0,180 | 36,492 | 0,161 |
| WikiC5 | GensimBatchVbLda | 0,826 | 0,848 | 0,777 | 0,845 |  | 342,528 | 0,644 | 0,054 | -0,710 | -1,329 | 0,914 | 0,169 | 36,246 | 0,160 |
| WikiC5 | GensimOnlineVbLda | 0,837 | 0,866 | 0,800 | 0,858 |  | 341,766 | 0,673 | 0,068 | -0,429 | -1,304 | 0,917 | 0,165 | 36,220 | 0,157 |
| WikiC5 | MalletGibbsLda | 0,959 | 0,968 | 0,953 | 0,965 |  | 353,084 | 0,739 | 0,098 | 0,205 | -0,900 | 0,931 | 0,137 | 35,116 | 0,146 |
| WikiC7 | SklearnKmeans | 0,780 | 0,785 | 0,684 | 0,758 |  |  | 0,661 | 0,048 | -0,780 | -1,847 | 0,891 | 0,993 | 45,217 | 0,259 |
| WikiC7 | SklearnLsi | 0,608 | 0,629 | 0,471 | 0,635 |  |  | 0,527 | -0,034 | -3,142 | -3,001 | 0,873 |  |  |  |
| WikiC7 | SklearnNmf | 0,804 | 0,804 | 0,729 | 0,790 |  |  | 0,800 | 0,120 | 0,357 | -0,924 | 0,936 | 0,100 | 1,272 | 0,149 |
| WikiC7 | SklearnPlsa | 0,801 | 0,801 | 0,665 | 0,722 |  |  | 0,499 | 0,002 | -0,858 | -1,102 | 0,880 | 0,341 | 1,678 | 0,166 |
| WikiC7 | SklearnBatchVbLda | 0,703 | 0,732 | 0,638 | 0,749 |  | 317,828 | 0,693 | 0,082 | 0,097 | -1,062 | 0,905 | 0,155 | 39,543 | 0,157 |
| WikiC7 | SklearnOnlineVbLda | 0,710 | 0,722 | 0,621 | 0,732 |  | 321,498 | 0,688 | 0,080 | 0,102 | -1,044 | 0,903 | 0,172 | 40,198 | 0,165 |
| WikiC7 | GensimBatchVbLda | 0,727 | 0,754 | 0,649 | 0,754 |  | 312,077 | 0,687 | 0,081 | 0,080 | -1,066 | 0,902 | 0,173 | 40,197 | 0,169 |
| WikiC7 | GensimOnlineVbLda | 0,717 | 0,734 | 0,641 | 0,740 |  | 314,957 | 0,690 | 0,080 | 0,098 | -1,073 | 0,905 | 0,166 | 40,018 | 0,161 |
| WikiC7 | MalletGibbsLda | 0,772 | 0,785 | 0,703 | 0,777 |  | 383,131 | 0,719 | 0,096 | 0,414 | -0,955 | 0,908 | 0,157 | 38,939 | 0,158 |
| WikiC10 | SklearnKmeans | 0,784 | 0,792 | 0,658 | 0,767 |  |  | 0,616 | 0,049 | -0,562 | -1,789 | 0,868 | 0,995 | 56,987 | 0,283 |
| WikiC10 | SklearnLsi | 0,698 | 0,698 | 0,508 | 0,706 |  |  | 0,593 | -0,013 | -2,468 | -3,003 | 0,856 |  |  |  |
| WikiC10 | SklearnNmf | 0,820 | 0,820 | 0,712 | 0,797 |  |  | 0,775 | 0,121 | 0,557 | -0,964 | 0,918 | 0,098 | 3,000 | 0,153 |
| WikiC10 | SklearnPlsa | 0,724 | 0,724 | 0,557 | 0,662 |  |  | 0,490 | 0,007 | -0,724 | -1,150 | 0,875 | 0,300 | 1,948 | 0,164 |
| WikiC10 | SklearnBatchVbLda | 0,691 | 0,721 | 0,613 | 0,732 |  | 315,989 | 0,654 | 0,080 | 0,283 | -1,066 | 0,884 | 0,161 | 44,666 | 0,158 |
| WikiC10 | SklearnOnlineVbLda | 0,641 | 0,669 | 0,570 | 0,702 |  | 321,913 | 0,649 | 0,076 | 0,187 | -1,086 | 0,883 | 0,174 | 45,581 | 0,167 |
| WikiC10 | GensimBatchVbLda | 0,652 | 0,678 | 0,575 | 0,709 |  | 315,989 | 0,624 | 0,067 | 0,012 | -1,176 | 0,877 | 0,192 | 46,529 | 0,177 |
| WikiC10 | GensimOnlineVbLda | 0,672 | 0,696 | 0,587 | 0,715 |  | 313,108 | 0,654 | 0,079 | 0,253 | -1,067 | 0,884 | 0,165 | 45,637 | 0,161 |
| WikiC10 | MalletGibbsLda | 0,778 | 0,788 | 0,679 | 0,760 |  | 407,730 | 0,679 | 0,094 | 0,548 | -0,997 | 0,889 | 0,159 | 43,932 | 0,160 |
| WikiC13 | SklearnKmeans | 0,697 | 0,732 | 0,587 | 0,733 |  |  | 0,577 | 0,041 | -0,450 | -1,693 | 0,840 | 0,995 | 68,135 | 0,293 |
| WikiC13 | SklearnLsi | 0,565 | 0,566 | 0,398 | 0,585 |  |  | 0,423 | -0,100 | -4,138 | -3,205 | 0,783 |  |  |  |
| WikiC13 | SklearnNmf | 0,723 | 0,762 | 0,634 | 0,755 |  |  | 0,699 | 0,091 | 0,330 | -1,112 | 0,884 | 0,090 | 6,825 | 0,151 |
| WikiC13 | SklearnPlsa | 0,590 | 0,595 | 0,446 | 0,567 |  |  | 0,489 | -0,002 | -0,916 | -1,281 | 0,856 | 0,259 | 2,529 | 0,156 |
| WikiC13 | SklearnBatchVbLda | 0,652 | 0,679 | 0,572 | 0,706 |  | 326,761 | 0,620 | 0,071 | 0,246 | -1,114 | 0,862 | 0,162 | 48,201 | 0,159 |
| WikiC13 | SklearnOnlineVbLda | 0,565 | 0,598 | 0,480 | 0,646 |  | 341,621 | 0,592 | 0,056 | -0,050 | -1,185 | 0,860 | 0,158 | 48,830 | 0,159 |
| WikiC13 | GensimBatchVbLda | 0,618 | 0,645 | 0,544 | 0,692 |  | 327,087 | 0,595 | 0,062 | 0,114 | -1,145 | 0,853 | 0,196 | 50,816 | 0,180 |
| WikiC13 | GensimOnlineVbLda | 0,582 | 0,612 | 0,515 | 0,669 |  | 331,839 | 0,604 | 0,061 | 0,017 | -1,164 | 0,864 | 0,141 | 48,105 | 0,150 |
| WikiC13 | MalletGibbsLda | 0,745 | 0,757 | 0,640 | 0,737 |  | 452,762 | 0,647 | 0,082 | 0,437 | -1,059 | 0,867 | 0,166 | 48,119 | 0,163 |
| WikiC15 | SklearnKmeans | 0,636 | 0,672 | 0,503 | 0,666 |  |  | 0,547 | 0,035 | -0,444 | -1,555 | 0,808 | 0,996 | 69,649 | 0,300 |
| WikiC15 | SklearnLsi | 0,553 | 0,583 | 0,384 | 0,575 |  |  | 0,437 | -0,071 | -3,342 | -2,810 | 0,769 |  |  |  |
| WikiC15 | SklearnNmf | 0,690 | 0,725 | 0,568 | 0,700 |  |  | 0,696 | 0,089 | 0,355 | -1,106 | 0,862 | 0,089 | 10,155 | 0,149 |
| WikiC15 | SklearnPlsa | 0,515 | 0,522 | 0,375 | 0,526 |  |  | 0,473 | 0,000 | -0,708 | -1,217 | 0,822 | 0,228 | 5,062 | 0,149 |
| WikiC15 | SklearnBatchVbLda | 0,587 | 0,623 | 0,499 | 0,654 |  | 318,927 | 0,608 | 0,068 | 0,277 | -1,133 | 0,834 | 0,158 | 47,992 | 0,157 |
| WikiC15 | SklearnOnlineVbLda | 0,485 | 0,518 | 0,401 | 0,595 |  | 334,634 | 0,584 | 0,055 | -0,017 | -1,162 | 0,832 | 0,138 | 48,940 | 0,152 |
| WikiC15 | GensimBatchVbLda | 0,592 | 0,621 | 0,498 | 0,655 |  | 319,071 | 0,588 | 0,061 | 0,159 | -1,135 | 0,826 | 0,192 | 50,386 | 0,179 |
| WikiC15 | GensimOnlineVbLda | 0,510 | 0,543 | 0,429 | 0,617 |  | 327,013 | 0,594 | 0,058 | 0,022 | -1,158 | 0,834 | 0,130 | 47,741 | 0,145 |
| WikiC15 | MalletGibbsLda | 0,695 | 0,706 | 0,576 | 0,695 |  | 462,448 | 0,632 | 0,079 | 0,481 | -1,082 | 0,839 | 0,168 | 47,567 | 0,163 |
| WikiC17 | SklearnKmeans | 0,636 | 0,659 | 0,483 | 0,658 |  |  | 0,519 | 0,026 | -0,517 | -1,439 | 0,802 | 0,996 | 76,298 | 0,307 |
| WikiC17 | SklearnLsi | 0,553 | 0,569 | 0,404 | 0,561 |  |  | 0,404 | -0,083 | -3,330 | -2,437 | 0,764 |  |  |  |
| WikiC17 | SklearnNmf | 0,653 | 0,690 | 0,546 | 0,690 |  |  | 0,668 | 0,071 | 0,070 | -1,213 | 0,852 | 0,088 | 9,971 | 0,147 |
| WikiC17 | SklearnPlsa | 0,523 | 0,544 | 0,390 | 0,524 |  |  | 0,471 | -0,003 | -0,850 | -1,346 | 0,829 | 0,205 | 7,412 | 0,147 |
| WikiC17 | SklearnBatchVbLda | 0,561 | 0,591 | 0,473 | 0,640 |  | 326,122 | 0,580 | 0,056 | 0,109 | -1,181 | 0,829 | 0,152 | 49,261 | 0,155 |
| WikiC17 | SklearnOnlineVbLda | 0,468 | 0,495 | 0,381 | 0,581 |  | 341,023 | 0,556 | 0,043 | -0,186 | -1,204 | 0,826 | 0,132 | 49,360 | 0,149 |
| WikiC17 | GensimBatchVbLda | 0,544 | 0,578 | 0,455 | 0,631 |  | 327,260 | 0,563 | 0,051 | 0,037 | -1,187 | 0,820 | 0,194 | 52,292 | 0,180 |
| WikiC17 | GensimOnlineVbLda | 0,496 | 0,521 | 0,401 | 0,598 |  | 331,960 | 0,574 | 0,048 | -0,082 | -1,190 | 0,832 | 0,120 | 47,317 | 0,141 |
| WikiC17 | MalletGibbsLda | 0,621 | 0,649 | 0,527 | 0,668 |  | 488,357 | 0,603 | 0,066 | 0,304 | -1,131 | 0,835 | 0,166 | 49,175 | 0,162 |
| WikiC20 | SklearnKmeans | 0,603 | 0,634 | 0,432 | 0,627 |  |  | 0,505 | 0,027 | -0,394 | -1,355 | 0,784 | 0,996 | 83,944 | 0,313 |
| WikiC20 | SklearnLsi | 0,494 | 0,506 | 0,343 | 0,514 |  |  | 0,344 | -0,132 | -4,392 | -3,029 | 0,718 |  |  |  |
| WikiC20 | SklearnNmf | 0,628 | 0,644 | 0,466 | 0,631 |  |  | 0,636 | 0,065 | 0,076 | -1,228 | 0,835 | 0,078 | 19,837 | 0,147 |
| WikiC20 | SklearnPlsa | 0,500 | 0,507 | 0,333 | 0,484 |  |  | 0,493 | 0,013 | -0,526 | -1,336 | 0,820 | 0,174 | 13,379 | 0,141 |
| WikiC20 | SklearnBatchVbLda | 0,552 | 0,579 | 0,443 | 0,615 |  | 305,593 | 0,587 | 0,064 | 0,279 | -1,205 | 0,819 | 0,139 | 50,283 | 0,152 |
| WikiC20 | SklearnOnlineVbLda | 0,408 | 0,434 | 0,328 | 0,535 |  | 321,634 | 0,562 | 0,048 | -0,068 | -1,246 | 0,819 | 0,115 | 50,586 | 0,142 |
| WikiC20 | GensimBatchVbLda | 0,516 | 0,545 | 0,419 | 0,602 |  | 309,058 | 0,558 | 0,053 | 0,122 | -1,206 | 0,808 | 0,179 | 54,678 | 0,177 |
| WikiC20 | GensimOnlineVbLda | 0,422 | 0,448 | 0,344 | 0,550 |  | 313,697 | 0,565 | 0,048 | -0,072 | -1,247 | 0,820 | 0,105 | 49,470 | 0,134 |
| WikiC20 | MalletGibbsLda | 0,621 | 0,635 | 0,492 | 0,637 |  | 479,714 | 0,597 | 0,069 | 0,396 | -1,179 | 0,820 | 0,155 | 51,254 | 0,160 |
| WikiC23 | SklearnKmeans | 0,605 | 0,632 | 0,411 | 0,627 |  |  | 0,495 | 0,030 | -0,246 | -1,368 | 0,763 | 0,996 | 84,920 | 0,315 |
| WikiC23 | SklearnLsi | 0,520 | 0,533 | 0,347 | 0,518 |  |  | 0,355 | -0,117 | -3,941 | -2,787 | 0,709 |  |  |  |
| WikiC23 | SklearnNmf | 0,636 | 0,663 | 0,488 | 0,644 |  |  | 0,636 | 0,070 | 0,218 | -1,215 | 0,824 | 0,076 | 28,775 | 0,150 |
| WikiC23 | SklearnPlsa | 0,437 | 0,445 | 0,293 | 0,449 |  |  | 0,487 | 0,013 | -0,537 | -1,334 | 0,812 | 0,132 | 27,537 | 0,131 |
| WikiC23 | SklearnBatchVbLda | 0,532 | 0,553 | 0,429 | 0,606 |  | 320,795 | 0,571 | 0,062 | 0,304 | -1,181 | 0,805 | 0,135 | 50,220 | 0,153 |
| WikiC23 | SklearnOnlineVbLda | 0,413 | 0,431 | 0,321 | 0,531 |  | 338,149 | 0,546 | 0,046 | -0,059 | -1,250 | 0,805 | 0,108 | 50,168 | 0,139 |
| WikiC23 | GensimBatchVbLda | 0,501 | 0,526 | 0,405 | 0,593 |  | 323,755 | 0,543 | 0,051 | 0,121 | -1,189 | 0,793 | 0,176 | 54,116 | 0,179 |
| WikiC23 | GensimOnlineVbLda | 0,427 | 0,446 | 0,342 | 0,549 |  | 329,926 | 0,551 | 0,049 | -0,012 | -1,256 | 0,808 | 0,098 | 48,974 | 0,131 |
| WikiC23 | MalletGibbsLda | 0,588 | 0,601 | 0,463 | 0,625 |  | 521,970 | 0,579 | 0,068 | 0,433 | -1,139 | 0,805 | 0,152 | 50,561 | 0,161 |
| WikiC25 | SklearnKmeans | 0,584 | 0,613 | 0,410 | 0,617 |  |  | 0,489 | 0,026 | -0,303 | -1,251 | 0,749 | 0,996 | 91,112 | 0,318 |
| WikiC25 | SklearnLsi | 0,479 | 0,497 | 0,328 | 0,497 |  |  | 0,326 | -0,120 | -3,953 | -2,775 | 0,690 |  |  |  |
| WikiC25 | SklearnNmf | 0,645 | 0,652 | 0,469 | 0,637 |  |  | 0,609 | 0,068 | 0,257 | -1,261 | 0,807 | 0,071 | 33,947 | 0,147 |
| WikiC25 | SklearnPlsa | 0,411 | 0,417 | 0,269 | 0,424 |  |  | 0,485 | 0,018 | -0,374 | -1,352 | 0,799 | 0,102 | 45,885 | 0,125 |
| WikiC25 | SklearnBatchVbLda | 0,524 | 0,550 | 0,420 | 0,608 |  | 322,853 | 0,560 | 0,058 | 0,269 | -1,219 | 0,791 | 0,127 | 52,250 | 0,151 |
| WikiC25 | SklearnOnlineVbLda | 0,392 | 0,413 | 0,305 | 0,527 |  | 341,214 | 0,531 | 0,041 | -0,099 | -1,277 | 0,790 | 0,100 | 52,602 | 0,136 |
| WikiC25 | GensimBatchVbLda | 0,491 | 0,520 | 0,395 | 0,592 |  | 326,983 | 0,535 | 0,049 | 0,132 | -1,217 | 0,779 | 0,168 | 56,993 | 0,178 |
| WikiC25 | GensimOnlineVbLda | 0,413 | 0,430 | 0,332 | 0,549 |  | 332,688 | 0,537 | 0,039 | -0,188 | -1,291 | 0,795 | 0,087 | 50,827 | 0,127 |
| WikiC25 | MalletGibbsLda | 0,576 | 0,593 | 0,459 | 0,626 |  | 535,101 | 0,576 | 0,066 | 0,413 | -1,189 | 0,792 | 0,145 | 53,052 | 0,159 |
| WikiC27 | SklearnKmeans | 0,543 | 0,579 | 0,380 | 0,591 |  |  | 0,487 | 0,027 | -0,262 | -1,271 | 0,751 | 0,997 | 96,302 | 0,321 |
| WikiC27 | SklearnLsi | 0,403 | 0,420 | 0,267 | 0,442 |  |  | 0,317 | -0,104 | -3,396 | -2,315 | 0,686 |  |  |  |
| WikiC27 | SklearnNmf | 0,562 | 0,584 | 0,409 | 0,589 |  |  | 0,606 | 0,061 | 0,164 | -1,330 | 0,810 | 0,068 | 41,890 | 0,143 |
| WikiC27 | SklearnPlsa | 0,363 | 0,374 | 0,229 | 0,390 |  |  | 0,472 | 0,008 | -0,564 | -1,423 | 0,800 | 0,093 | 55,629 | 0,120 |
| WikiC27 | SklearnBatchVbLda | 0,502 | 0,529 | 0,403 | 0,594 |  | 319,663 | 0,559 | 0,058 | 0,279 | -1,251 | 0,793 | 0,122 | 52,469 | 0,148 |
| WikiC27 | SklearnOnlineVbLda | 0,362 | 0,379 | 0,280 | 0,504 |  | 338,982 | 0,533 | 0,039 | -0,143 | -1,312 | 0,792 | 0,096 | 54,150 | 0,133 |
| WikiC27 | GensimBatchVbLda | 0,469 | 0,499 | 0,373 | 0,575 |  | 324,690 | 0,534 | 0,048 | 0,130 | -1,260 | 0,783 | 0,166 | 58,629 | 0,176 |
| WikiC27 | GensimOnlineVbLda | 0,383 | 0,401 | 0,297 | 0,519 |  | 329,343 | 0,540 | 0,041 | -0,120 | -1,316 | 0,799 | 0,082 | 50,899 | 0,125 |
| WikiC27 | MalletGibbsLda | 0,562 | 0,581 | 0,447 | 0,620 |  | 541,287 | 0,571 | 0,065 | 0,435 | -1,219 | 0,794 | 0,142 | 54,125 | 0,157 |
| WikiC30 | SklearnKmeans | 0,498 | 0,533 | 0,350 | 0,574 |  |  | 0,478 | 0,027 | -0,258 | -1,208 | 0,731 | 0,997 | 97,982 | 0,322 |
| WikiC30 | SklearnLsi | 0,391 | 0,420 | 0,282 | 0,470 |  |  | 0,324 | -0,097 | -3,210 | -2,236 | 0,665 |  |  |  |
| WikiC30 | SklearnNmf | 0,517 | 0,548 | 0,381 | 0,582 |  |  | 0,586 | 0,058 | 0,160 | -1,281 | 0,784 | 0,062 | 62,879 | 0,140 |
| WikiC30 | SklearnPlsa | 0,311 | 0,330 | 0,209 | 0,371 |  |  | 0,475 | 0,015 | -0,408 | -1,365 | 0,785 | 0,081 | 81,459 | 0,116 |
| WikiC30 | SklearnBatchVbLda | 0,470 | 0,498 | 0,367 | 0,569 |  | 331,728 | 0,557 | 0,062 | 0,381 | -1,204 | 0,775 | 0,121 | 50,733 | 0,147 |
| WikiC30 | SklearnOnlineVbLda | 0,330 | 0,350 | 0,255 | 0,486 |  | 351,715 | 0,526 | 0,043 | -0,026 | -1,274 | 0,773 | 0,091 | 51,982 | 0,130 |
| WikiC30 | GensimBatchVbLda | 0,443 | 0,466 | 0,350 | 0,560 |  | 337,573 | 0,530 | 0,053 | 0,246 | -1,202 | 0,762 | 0,162 | 56,434 | 0,175 |
| WikiC30 | GensimOnlineVbLda | 0,354 | 0,370 | 0,278 | 0,505 |  | 341,371 | 0,535 | 0,043 | -0,081 | -1,288 | 0,783 | 0,085 | 59,683 | 0,124 |
| WikiC30 | MalletGibbsLda | 0,526 | 0,549 | 0,407 | 0,593 |  | 584,821 | 0,569 | 0,069 | 0,508 | -1,160 | 0,774 | 0,139 | 52,037 | 0,156 |
| WikiC35 | SklearnKmeans | 0,495 | 0,531 | 0,333 | 0,567 |  |  | 0,473 | 0,028 | -0,138 | -1,219 | 0,718 | 0,997 | 104,249 | 0,326 |
| WikiC35 | SklearnLsi | 0,368 | 0,379 | 0,223 | 0,395 |  |  | 0,274 | -0,104 | -3,207 | -2,325 | 0,648 |  |  |  |
| WikiC35 | SklearnNmf | 0,499 | 0,527 | 0,357 | 0,554 |  |  | 0,578 | 0,060 | 0,238 | -1,357 | 0,781 | 0,055 | 88,271 | 0,136 |
| WikiC35 | SklearnPlsa | 0,282 | 0,291 | 0,189 | 0,340 |  |  | 0,459 | 0,012 | -0,443 | -1,440 | 0,784 | 0,048 | 171,591 | 0,104 |
| WikiC35 | SklearnBatchVbLda | 0,467 | 0,488 | 0,363 | 0,575 |  | 322,845 | 0,545 | 0,059 | 0,367 | -1,239 | 0,766 | 0,109 | 50,162 | 0,144 |
| WikiC35 | SklearnOnlineVbLda | 0,301 | 0,316 | 0,245 | 0,489 |  | 351,857 | 0,513 | 0,038 | -0,087 | -1,324 | 0,755 | 0,105 | 75,499 | 0,132 |
| WikiC35 | GensimBatchVbLda | 0,468 | 0,497 | 0,373 | 0,591 |  | 354,360 | 0,555 | 0,065 | 0,411 | -1,201 | 0,719 | 0,140 | 57,036 | 0,170 |
| WikiC35 | GensimOnlineVbLda | 0,349 | 0,364 | 0,288 | 0,533 |  | 362,146 | 0,535 | 0,045 | -0,090 | -1,302 | 0,734 | 0,072 | 64,856 | 0,119 |
| WikiC35 | MalletGibbsLda | 0,546 | 0,566 | 0,435 | 0,625 |  | 619,173 | 0,595 | 0,080 | 0,630 | -1,180 | 0,735 | 0,121 | 52,922 | 0,150 |
| WikiC40 | SklearnKmeans | 0,473 | 0,504 | 0,310 | 0,544 |  |  | 0,460 | 0,025 | -0,167 | -1,211 | 0,701 | 0,997 | 113,243 | 0,330 |
| WikiC40 | SklearnLsi | 0,312 | 0,329 | 0,183 | 0,367 |  |  | 0,285 | -0,091 | -2,841 | -2,267 | 0,638 |  |  |  |
| WikiC40 | SklearnNmf | 0,447 | 0,477 | 0,296 | 0,509 |  |  | 0,557 | 0,053 | 0,174 | -1,368 | 0,770 | 0,049 | 44,137 | 0,134 |
| WikiC40 | SklearnPlsa | 0,471 | 0,504 | 0,367 | 0,565 |  |  | 0,565 | 0,057 | 0,198 | -1,342 | 0,784 | 0,047 | 150,083 | 0,121 |
| WikiC40 | SklearnBatchVbLda | 0,440 | 0,465 | 0,337 | 0,554 |  | 327,798 | 0,528 | 0,054 | 0,334 | -1,249 | 0,753 | 0,099 | 51,251 | 0,142 |
| WikiC40 | SklearnOnlineVbLda | 0,285 | 0,298 | 0,220 | 0,459 |  | 350,983 | 0,499 | 0,034 | -0,114 | -1,333 | 0,755 | 0,084 | 65,131 | 0,125 |
| WikiC40 | GensimBatchVbLda | 0,422 | 0,448 | 0,326 | 0,546 |  | 333,686 | 0,511 | 0,048 | 0,254 | -1,242 | 0,741 | 0,141 | 58,905 | 0,173 |
| WikiC40 | GensimOnlineVbLda | 0,308 | 0,321 | 0,241 | 0,478 |  | 338,346 | 0,508 | 0,036 | -0,089 | -1,339 | 0,760 | 0,073 | 83,918 | 0,118 |
| WikiC40 | MalletGibbsLda | 0,553 | 0,572 | 0,428 | 0,619 |  | 670,091 | 0,578 | 0,075 | 0,619 | -1,154 | 0,713 | 0,118 | 55,989 | 0,150 |
| WikiC45 | SklearnKmeans | 0,450 | 0,479 | 0,289 | 0,527 |  |  | 0,449 | 0,026 | -0,055 | -1,150 | 0,671 | 0,997 | 115,151 | 0,334 |
| WikiC45 | SklearnLsi | 0,291 | 0,313 | 0,173 | 0,346 |  |  | 0,264 | -0,082 | -2,408 | -2,009 | 0,609 |  |  |  |
| WikiC45 | SklearnNmf | 0,427 | 0,450 | 0,269 | 0,479 |  |  | 0,537 | 0,050 | 0,189 | -1,372 | 0,747 | 0,041 | 41,635 | 0,132 |
| WikiC45 | SklearnPlsa | 0,410 | 0,431 | 0,328 | 0,536 |  |  | 0,546 | 0,051 | 0,160 | -1,343 | 0,765 | 0,040 | 209,849 | 0,116 |
| WikiC45 | SklearnBatchVbLda | 0,406 | 0,432 | 0,307 | 0,535 |  | 331,328 | 0,515 | 0,052 | 0,330 | -1,251 | 0,729 | 0,092 | 49,234 | 0,140 |
| WikiC45 | SklearnOnlineVbLda | 0,264 | 0,277 | 0,207 | 0,457 |  | 354,598 | 0,482 | 0,032 | -0,071 | -1,333 | 0,730 | 0,078 | 72,847 | 0,124 |
| WikiC45 | GensimBatchVbLda | 0,398 | 0,422 | 0,307 | 0,539 |  | 336,627 | 0,493 | 0,045 | 0,250 | -1,231 | 0,714 | 0,132 | 57,069 | 0,172 |
| WikiC45 | GensimOnlineVbLda | 0,271 | 0,284 | 0,215 | 0,467 |  | 341,701 | 0,501 | 0,037 | 0,019 | -1,341 | 0,741 | 0,064 | 99,091 | 0,115 |
| WikiC45 | MalletGibbsLda | 0,517 | 0,538 | 0,396 | 0,602 |  | 691,596 | 0,561 | 0,071 | 0,588 | -1,191 | 0,698 | 0,109 | 54,300 | 0,148 |
| WikiC50 | SklearnKmeans | 0,421 | 0,449 | 0,257 | 0,497 |  |  | 0,442 | 0,024 | -0,042 | -1,202 | 0,656 | 0,997 | 123,817 | 0,336 |
| WikiC50 | SklearnLsi | 0,250 | 0,270 | 0,142 | 0,305 |  |  | 0,260 | -0,084 | -2,410 | -2,100 | 0,602 |  |  |  |
| WikiC50 | SklearnNmf | 0,401 | 0,414 | 0,241 | 0,453 |  |  | 0,522 | 0,045 | 0,116 | -1,431 | 0,740 | 0,037 | 42,746 | 0,130 |
| WikiC50 | SklearnPlsa | 0,392 | 0,413 | 0,293 | 0,496 |  |  | 0,529 | 0,048 | 0,156 | -1,362 | 0,755 | 0,031 | 292,562 | 0,112 |
| WikiC50 | SklearnBatchVbLda | 0,397 | 0,418 | 0,288 | 0,516 |  | 324,569 | 0,504 | 0,049 | 0,324 | -1,279 | 0,720 | 0,085 | 49,194 | 0,138 |
| WikiC50 | SklearnOnlineVbLda | 0,237 | 0,244 | 0,187 | 0,435 |  | 351,264 | 0,466 | 0,025 | -0,176 | -1,385 | 0,719 | 0,117 | 127,418 | 0,136 |
| WikiC50 | GensimBatchVbLda | 0,373 | 0,399 | 0,281 | 0,515 |  | 331,455 | 0,483 | 0,042 | 0,240 | -1,258 | 0,705 | 0,126 | 59,326 | 0,170 |
| WikiC50 | GensimOnlineVbLda | 0,259 | 0,269 | 0,198 | 0,443 |  | 333,543 | 0,476 | 0,027 | -0,179 | -1,391 | 0,732 | 0,074 | 159,493 | 0,117 |
| WikiC50 | MalletGibbsLda | 0,494 | 0,512 | 0,362 | 0,572 |  | 677,991 | 0,556 | 0,070 | 0,592 | -1,216 | 0,692 | 0,106 | 55,421 | 0,146 |
| WikiC55 | SklearnKmeans | 0,426 | 0,453 | 0,248 | 0,487 |  |  | 0,435 | 0,022 | -0,070 | -1,184 | 0,643 | 0,997 | 130,299 | 0,339 |
| WikiC55 | SklearnLsi | 0,261 | 0,281 | 0,148 | 0,315 |  |  | 0,255 | -0,082 | -2,294 | -2,005 | 0,589 |  |  |  |
| WikiC55 | SklearnNmf | 0,401 | 0,417 | 0,237 | 0,445 |  |  | 0,503 | 0,040 | 0,105 | -1,449 | 0,718 | 0,034 | 39,553 | 0,128 |
| WikiC55 | SklearnPlsa | 0,353 | 0,374 | 0,268 | 0,474 |  |  | 0,505 | 0,043 | 0,155 | -1,389 | 0,737 | 0,024 | 381,775 | 0,109 |
| WikiC55 | SklearnBatchVbLda | 0,394 | 0,415 | 0,285 | 0,511 |  | 321,027 | 0,494 | 0,047 | 0,325 | -1,279 | 0,705 | 0,076 | 47,622 | 0,136 |
| WikiC55 | SklearnOnlineVbLda | 0,221 | 0,229 | 0,172 | 0,416 |  | 350,403 | 0,449 | 0,021 | -0,193 | -1,389 | 0,703 | 0,116 | 136,830 | 0,136 |
| WikiC55 | GensimBatchVbLda | 0,378 | 0,401 | 0,279 | 0,511 |  | 328,160 | 0,474 | 0,040 | 0,237 | -1,258 | 0,689 | 0,115 | 57,011 | 0,169 |
| WikiC55 | GensimOnlineVbLda | 0,239 | 0,248 | 0,179 | 0,425 |  | 332,017 | 0,437 | -0,001 | -0,838 | -1,482 | 0,711 | 0,075 | 186,996 | 0,117 |
| WikiC55 | MalletGibbsLda | 0,476 | 0,494 | 0,346 | 0,563 |  | 677,979 | 0,547 | 0,067 | 0,579 | -1,230 | 0,686 | 0,105 | 55,403 | 0,144 |
| WikiC60 | SklearnKmeans | 0,394 | 0,421 | 0,218 | 0,464 |  |  | 0,427 | 0,020 | -0,080 | -1,192 | 0,633 | 0,997 | 134,825 | 0,341 |
| WikiC60 | SklearnLsi | 0,252 | 0,271 | 0,127 | 0,298 |  |  | 0,235 | -0,080 | -2,154 | -1,985 | 0,573 |  |  |  |
| WikiC60 | SklearnNmf | 0,363 | 0,381 | 0,206 | 0,421 |  |  | 0,494 | 0,039 | 0,118 | -1,456 | 0,707 | 0,030 | 39,650 | 0,126 |
| WikiC60 | SklearnPlsa | 0,349 | 0,370 | 0,247 | 0,461 |  |  | 0,496 | 0,042 | 0,183 | -1,370 | 0,725 | 0,021 | 477,565 | 0,107 |
| WikiC60 | SklearnBatchVbLda | 0,372 | 0,394 | 0,264 | 0,497 |  | 316,680 | 0,485 | 0,045 | 0,316 | -1,279 | 0,693 | 0,069 | 45,826 | 0,134 |
| WikiC60 | SklearnOnlineVbLda | 0,205 | 0,213 | 0,163 | 0,408 |  | 347,386 | 0,444 | 0,020 | -0,194 | -1,405 | 0,692 | 0,126 | 165,945 | 0,139 |
| WikiC60 | GensimBatchVbLda | 0,359 | 0,384 | 0,257 | 0,494 |  | 323,507 | 0,472 | 0,041 | 0,265 | -1,263 | 0,679 | 0,106 | 56,540 | 0,168 |
| WikiC60 | GensimOnlineVbLda | 0,218 | 0,227 | 0,166 | 0,411 |  | 326,765 | 0,431 | 0,003 | -0,694 | -1,501 | 0,704 | 0,077 | 207,570 | 0,117 |
| WikiC60 | MalletGibbsLda | 0,477 | 0,497 | 0,342 | 0,562 |  | 684,757 | 0,546 | 0,067 | 0,580 | -1,218 | 0,675 | 0,094 | 54,533 | 0,143 |
| WikiC65 | SklearnKmeans | 0,397 | 0,429 | 0,229 | 0,469 |  |  | 0,428 | 0,021 | -0,058 | -1,161 | 0,625 | 0,997 | 138,462 | 0,341 |
| WikiC65 | SklearnLsi | 0,245 | 0,262 | 0,133 | 0,291 |  |  | 0,240 | -0,073 | -1,963 | -2,009 | 0,568 |  |  |  |
| WikiC65 | SklearnNmf | 0,348 | 0,369 | 0,200 | 0,413 |  |  | 0,494 | 0,040 | 0,161 | -1,457 | 0,699 | 0,028 | 38,726 | 0,124 |
| WikiC65 | SklearnPlsa | 0,319 | 0,338 | 0,245 | 0,454 |  |  | 0,483 | 0,038 | 0,134 | -1,372 | 0,714 | 0,018 | 589,152 | 0,104 |
| WikiC65 | SklearnBatchVbLda | 0,361 | 0,382 | 0,259 | 0,489 |  | 315,309 | 0,482 | 0,045 | 0,324 | -1,295 | 0,686 | 0,063 | 44,471 | 0,131 |
| WikiC65 | SklearnOnlineVbLda | 0,186 | 0,194 | 0,148 | 0,399 |  | 348,842 | 0,435 | 0,017 | -0,227 | -1,435 | 0,682 | 0,138 | 201,362 | 0,143 |
| WikiC65 | GensimBatchVbLda | 0,350 | 0,371 | 0,254 | 0,491 |  | 321,977 | 0,465 | 0,039 | 0,252 | -1,278 | 0,669 | 0,102 | 56,067 | 0,166 |
| WikiC65 | GensimOnlineVbLda | 0,204 | 0,212 | 0,160 | 0,407 |  | 326,767 | 0,423 | 0,014 | -0,288 | -1,485 | 0,688 | 0,084 | 285,248 | 0,118 |
| WikiC65 | MalletGibbsLda | 0,458 | 0,481 | 0,338 | 0,555 |  | 684,753 | 0,545 | 0,059 | 0,576 | -1,229 | 0,667 | 0,090 | 54,517 | 0,132 |
| WikiC70 | SklearnKmeans | 0,383 | 0,410 | 0,225 | 0,466 |  |  | 0,427 | 0,021 | -0,065 | -1,179 | 0,617 | 0,997 | 142,438 | 0,340 |
| WikiC70 | SklearnLsi | 0,234 | 0,247 | 0,135 | 0,296 |  |  | 0,236 | -0,071 | -1,913 | -2,014 | 0,562 |  |  |  |
| WikiC70 | SklearnNmf | 0,323 | 0,346 | 0,182 | 0,398 |  |  | 0,491 | 0,041 | 0,188 | -1,471 | 0,692 | 0,025 | 37,902 | 0,123 |
| WikiC70 | SklearnPlsa | 0,318 | 0,333 | 0,237 | 0,450 |  |  | 0,473 | 0,037 | 0,140 | -1,379 | 0,707 | 0,015 | 722,123 | 0,102 |
| WikiC70 | SklearnBatchVbLda | 0,354 | 0,373 | 0,253 | 0,489 |  | 309,179 | 0,479 | 0,045 | 0,327 | -1,307 | 0,676 | 0,058 | 43,165 | 0,129 |
| WikiC70 | SklearnOnlineVbLda | 0,182 | 0,188 | 0,148 | 0,403 |  | 341,774 | 0,433 | 0,018 | -0,213 | -1,452 | 0,672 | 0,149 | 234,773 | 0,147 |
| WikiC70 | GensimBatchVbLda | 0,340 | 0,362 | 0,248 | 0,489 |  | 315,985 | 0,463 | 0,039 | 0,260 | -1,283 | 0,659 | 0,094 | 56,238 | 0,164 |
| WikiC70 | GensimOnlineVbLda | 0,191 | 0,199 | 0,147 | 0,404 |  | 319,698 | 0,425 | 0,014 | -0,283 | -1,510 | 0,680 | 0,085 | 309,170 | 0,119 |
| WikiC70 | MalletGibbsLda | 0,446 | 0,471 | 0,330 | 0,546 |  | 684,751 | 0,533 | 0,042 | 0,572 | -1,238 | 0,659 | 0,090 | 54,514 | 0,125 |
| WikiC75 | SklearnKmeans | 0,359 | 0,386 | 0,202 | 0,440 |  |  | 0,413 | 0,018 | -0,060 | -1,202 | 0,604 | 0,997 | 147,496 | 0,342 |
| WikiC75 | SklearnLsi | 0,213 | 0,225 | 0,112 | 0,258 |  |  | 0,227 | -0,066 | -1,695 | -1,955 | 0,550 |  |  |  |
| WikiC75 | SklearnNmf | 0,297 | 0,320 | 0,152 | 0,369 |  |  | 0,480 | 0,039 | 0,186 | -1,487 | 0,684 | 0,023 | 38,087 | 0,121 |
| WikiC75 | SklearnPlsa | 0,264 | 0,287 | 0,194 | 0,411 |  |  | 0,455 | 0,033 | 0,106 | -1,379 | 0,699 | 0,013 | 873,532 | 0,100 |
| WikiC75 | SklearnBatchVbLda | 0,329 | 0,348 | 0,231 | 0,466 |  | 315,565 | 0,472 | 0,043 | 0,317 | -1,312 | 0,671 | 0,054 | 41,985 | 0,127 |
| WikiC75 | SklearnOnlineVbLda | 0,170 | 0,178 | 0,130 | 0,379 |  | 348,890 | 0,427 | 0,017 | -0,190 | -1,455 | 0,666 | 0,141 | 235,315 | 0,144 |
| WikiC75 | GensimBatchVbLda | 0,316 | 0,337 | 0,225 | 0,467 |  | 322,355 | 0,458 | 0,038 | 0,265 | -1,285 | 0,653 | 0,092 | 55,432 | 0,164 |
| WikiC75 | GensimOnlineVbLda | 0,178 | 0,187 | 0,136 | 0,381 |  | 324,282 | 0,402 | 0,008 | -0,384 | -1,544 | 0,669 | 0,090 | 327,046 | 0,120 |
| WikiC75 | MalletGibbsLda | 0,423 | 0,442 | 0,296 | 0,521 |  | 689,337 | 0,530 | 0,063 | 0,558 | -1,260 | 0,658 | 0,081 | 52,717 | 0,138 |
| WikiD2 | SklearnKmeans | 0,987 | 0,987 | 0,950 | 0,912 |  |  | 0,485 | -0,027 | -1,647 | -1,240 | 0,957 | 0,981 | 22,965 | 0,168 |
| WikiD2 | SklearnLsi | 0,739 | 0,739 | 0,254 | 0,346 |  |  | 0,436 | -0,061 | -2,578 | -1,269 | 0,937 |  |  |  |
| WikiD2 | SklearnNmf | 0,990 | 0,990 | 0,959 | 0,927 |  |  | 0,564 | -0,003 | -1,200 | -1,021 | 0,961 | 0,174 | 29,808 | 0,122 |
| WikiD2 | SklearnPlsa | 0,969 | 0,969 | 0,880 | 0,831 |  |  | 0,515 | -0,014 | -1,349 | -1,026 | 0,961 | 0,204 | 3,021 | 0,124 |
| WikiD2 | SklearnBatchVbLda | 0,990 | 0,990 | 0,959 | 0,927 |  | 748,659 | 0,504 | 0,018 | -0,370 | -0,744 | 0,958 | 0,275 | 29,303 | 0,145 |
| WikiD2 | SklearnOnlineVbLda | 0,944 | 0,944 | 0,830 | 0,788 |  | 779,658 | 0,459 | 0,003 | -0,619 | -0,791 | 0,954 | 0,379 | 29,820 | 0,168 |
| WikiD2 | GensimBatchVbLda | 0,966 | 0,966 | 0,887 | 0,847 |  | 747,332 | 0,483 | 0,010 | -0,529 | -0,780 | 0,956 | 0,313 | 29,497 | 0,153 |
| WikiD2 | GensimOnlineVbLda | 0,965 | 0,965 | 0,870 | 0,816 |  | 751,696 | 0,481 | 0,013 | -0,422 | -0,740 | 0,957 | 0,357 | 29,754 | 0,166 |
| WikiD2 | MalletGibbsLda | 0,990 | 0,990 | 0,959 | 0,927 |  | 777,362 | 0,503 | 0,019 | -0,364 | -0,742 | 0,958 | 0,263 | 29,019 | 0,134 |
| WikiD3 | SklearnKmeans | 0,990 | 0,990 | 0,971 | 0,958 |  |  | 0,547 | 0,004 | -1,139 | -1,335 | 0,906 | 0,990 | 30,009 | 0,208 |
| WikiD3 | SklearnLsi | 0,805 | 0,805 | 0,488 | 0,588 |  |  | 0,598 | 0,021 | -0,917 | -1,061 | 0,914 |  |  |  |
| WikiD3 | SklearnNmf | 0,986 | 0,986 | 0,959 | 0,939 |  |  | 0,650 | 0,061 | -0,053 | -0,864 | 0,928 | 0,194 | 33,728 | 0,141 |
| WikiD3 | SklearnPlsa | 0,986 | 0,986 | 0,959 | 0,939 |  |  | 0,639 | 0,049 | -0,371 | -1,014 | 0,922 | 0,225 | 1,880 | 0,144 |
| WikiD3 | SklearnBatchVbLda | 0,933 | 0,942 | 0,887 | 0,881 |  | 664,729 | 0,571 | 0,043 | 0,014 | -0,795 | 0,907 | 0,311 | 33,694 | 0,165 |
| WikiD3 | SklearnOnlineVbLda | 0,785 | 0,820 | 0,661 | 0,698 |  | 706,377 | 0,513 | 0,022 | -0,272 | -0,881 | 0,899 | 0,401 | 35,031 | 0,185 |
| WikiD3 | GensimBatchVbLda | 0,939 | 0,941 | 0,869 | 0,863 |  | 656,010 | 0,561 | 0,041 | 0,014 | -0,777 | 0,906 | 0,340 | 33,758 | 0,173 |
| WikiD3 | GensimOnlineVbLda | 0,860 | 0,875 | 0,753 | 0,764 |  | 677,245 | 0,534 | 0,030 | -0,115 | -0,841 | 0,902 | 0,372 | 34,235 | 0,179 |
| WikiD3 | MalletGibbsLda | 0,985 | 0,985 | 0,955 | 0,934 |  | 732,290 | 0,588 | 0,050 | 0,132 | -0,787 | 0,907 | 0,288 | 33,233 | 0,154 |
| WikiD5 | SklearnKmeans | 0,954 | 0,954 | 0,890 | 0,885 |  |  | 0,583 | 0,013 | -1,099 | -1,761 | 0,864 | 0,994 | 41,362 | 0,240 |
| WikiD5 | SklearnLsi | 0,888 | 0,888 | 0,709 | 0,767 |  |  | 0,598 | 0,021 | -1,198 | -1,529 | 0,874 |  |  |  |
| WikiD5 | SklearnNmf | 0,972 | 0,972 | 0,930 | 0,919 |  |  | 0,696 | 0,065 | -0,257 | -0,980 | 0,894 | 0,153 | 40,449 | 0,150 |
| WikiD5 | SklearnPlsa | 0,976 | 0,976 | 0,940 | 0,933 |  |  | 0,660 | 0,048 | -0,565 | -1,044 | 0,888 | 0,179 | 1,757 | 0,152 |
| WikiD5 | SklearnBatchVbLda | 0,821 | 0,849 | 0,762 | 0,812 |  | 551,475 | 0,554 | 0,034 | -0,500 | -1,129 | 0,862 | 0,225 | 39,928 | 0,166 |
| WikiD5 | SklearnOnlineVbLda | 0,842 | 0,859 | 0,756 | 0,796 |  | 559,025 | 0,564 | 0,036 | -0,396 | -1,033 | 0,862 | 0,271 | 40,748 | 0,181 |
| WikiD5 | GensimBatchVbLda | 0,798 | 0,820 | 0,708 | 0,759 |  | 549,751 | 0,533 | 0,023 | -0,695 | -1,182 | 0,855 | 0,269 | 40,699 | 0,183 |
| WikiD5 | GensimOnlineVbLda | 0,882 | 0,889 | 0,804 | 0,828 |  | 542,144 | 0,579 | 0,041 | -0,322 | -1,032 | 0,863 | 0,255 | 40,703 | 0,175 |
| WikiD5 | MalletGibbsLda | 0,840 | 0,858 | 0,788 | 0,841 |  | 653,964 | 0,561 | 0,040 | -0,370 | -1,024 | 0,870 | 0,224 | 39,893 | 0,163 |
| WikiD7 | SklearnKmeans | 0,896 | 0,909 | 0,858 | 0,898 |  |  | 0,591 | 0,029 | -0,769 | -1,538 | 0,814 | 0,994 | 43,038 | 0,254 |
| WikiD7 | SklearnLsi | 0,893 | 0,893 | 0,725 | 0,800 |  |  | 0,505 | -0,035 | -2,518 | -2,257 | 0,791 |  |  |  |
| WikiD7 | SklearnNmf | 0,977 | 0,977 | 0,947 | 0,947 |  |  | 0,744 | 0,087 | -0,021 | -1,045 | 0,874 | 0,138 | 38,655 | 0,151 |
| WikiD7 | SklearnPlsa | 0,977 | 0,977 | 0,947 | 0,945 |  |  | 0,729 | 0,075 | -0,208 | -1,073 | 0,867 | 0,161 | 1,436 | 0,154 |
| WikiD7 | SklearnBatchVbLda | 0,816 | 0,851 | 0,779 | 0,841 |  | 470,472 | 0,602 | 0,053 | -0,143 | -1,082 | 0,831 | 0,206 | 38,581 | 0,163 |
| WikiD7 | SklearnOnlineVbLda | 0,805 | 0,832 | 0,734 | 0,799 |  | 481,052 | 0,565 | 0,041 | -0,346 | -1,155 | 0,826 | 0,244 | 39,583 | 0,176 |
| WikiD7 | GensimBatchVbLda | 0,830 | 0,848 | 0,774 | 0,829 |  | 464,168 | 0,590 | 0,050 | -0,187 | -1,081 | 0,827 | 0,241 | 39,476 | 0,181 |
| WikiD7 | GensimOnlineVbLda | 0,816 | 0,839 | 0,760 | 0,825 |  | 463,378 | 0,594 | 0,051 | -0,166 | -1,077 | 0,832 | 0,223 | 39,041 | 0,170 |
| WikiD7 | MalletGibbsLda | 0,897 | 0,916 | 0,858 | 0,884 |  | 567,244 | 0,636 | 0,068 | 0,176 | -0,993 | 0,834 | 0,213 | 38,566 | 0,164 |
| WikiD10 | SklearnKmeans | 0,806 | 0,839 | 0,725 | 0,810 |  |  | 0,556 | 0,041 | -0,434 | -1,523 | 0,792 | 0,993 | 51,321 | 0,271 |
| WikiD10 | SklearnLsi | 0,738 | 0,767 | 0,576 | 0,714 |  |  | 0,456 | -0,055 | -2,947 | -2,092 | 0,760 |  |  |  |
| WikiD10 | SklearnNmf | 0,803 | 0,846 | 0,757 | 0,831 |  |  | 0,720 | 0,103 | 0,403 | -1,048 | 0,849 | 0,120 | 42,580 | 0,154 |
| WikiD10 | SklearnPlsa | 0,785 | 0,836 | 0,744 | 0,820 |  |  | 0,720 | 0,098 | 0,403 | -1,093 | 0,845 | 0,150 | 3,726 | 0,154 |
| WikiD10 | SklearnBatchVbLda | 0,745 | 0,785 | 0,680 | 0,777 |  | 443,941 | 0,597 | 0,067 | 0,204 | -1,067 | 0,804 | 0,201 | 43,732 | 0,164 |
| WikiD10 | SklearnOnlineVbLda | 0,674 | 0,716 | 0,604 | 0,722 |  | 455,028 | 0,573 | 0,057 | 0,034 | -1,096 | 0,802 | 0,220 | 44,789 | 0,174 |
| WikiD10 | GensimBatchVbLda | 0,706 | 0,740 | 0,641 | 0,750 |  | 445,014 | 0,563 | 0,054 | -0,002 | -1,100 | 0,797 | 0,243 | 45,451 | 0,186 |
| WikiD10 | GensimOnlineVbLda | 0,700 | 0,741 | 0,646 | 0,752 |  | 439,552 | 0,591 | 0,064 | 0,155 | -1,059 | 0,806 | 0,205 | 44,097 | 0,168 |
| WikiD10 | MalletGibbsLda | 0,847 | 0,867 | 0,774 | 0,829 |  | 589,039 | 0,623 | 0,079 | 0,437 | -1,010 | 0,806 | 0,216 | 44,073 | 0,168 |
| WikiD13 | SklearnKmeans | 0,801 | 0,820 | 0,674 | 0,787 |  |  | 0,542 | 0,038 | -0,390 | -1,577 | 0,774 | 0,994 | 61,530 | 0,280 |
| WikiD13 | SklearnLsi | 0,653 | 0,694 | 0,447 | 0,638 |  |  | 0,461 | -0,037 | -2,267 | -2,234 | 0,750 |  |  |  |
| WikiD13 | SklearnNmf | 0,818 | 0,837 | 0,747 | 0,808 |  |  | 0,693 | 0,088 | 0,276 | -1,103 | 0,826 | 0,108 | 43,738 | 0,153 |
| WikiD13 | SklearnPlsa | 0,890 | 0,890 | 0,776 | 0,816 |  |  | 0,703 | 0,091 | 0,410 | -1,117 | 0,827 | 0,140 | 6,366 | 0,156 |
| WikiD13 | SklearnBatchVbLda | 0,728 | 0,746 | 0,625 | 0,734 |  | 446,417 | 0,588 | 0,065 | 0,243 | -1,115 | 0,792 | 0,186 | 46,482 | 0,163 |
| WikiD13 | SklearnOnlineVbLda | 0,604 | 0,638 | 0,521 | 0,661 |  | 462,846 | 0,564 | 0,056 | 0,099 | -1,150 | 0,789 | 0,198 | 47,480 | 0,169 |
| WikiD13 | GensimBatchVbLda | 0,674 | 0,703 | 0,590 | 0,714 |  | 447,727 | 0,559 | 0,055 | 0,070 | -1,140 | 0,784 | 0,231 | 48,719 | 0,187 |
| WikiD13 | GensimOnlineVbLda | 0,653 | 0,681 | 0,570 | 0,693 |  | 450,486 | 0,580 | 0,060 | 0,162 | -1,123 | 0,793 | 0,180 | 46,246 | 0,160 |
| WikiD13 | MalletGibbsLda | 0,789 | 0,799 | 0,680 | 0,766 |  | 645,136 | 0,606 | 0,073 | 0,401 | -1,090 | 0,794 | 0,194 | 46,773 | 0,167 |
| WikiD15 | SklearnKmeans | 0,721 | 0,750 | 0,584 | 0,728 |  |  | 0,518 | 0,034 | -0,286 | -1,155 | 0,758 | 0,993 | 66,218 | 0,285 |
| WikiD15 | SklearnLsi | 0,558 | 0,597 | 0,418 | 0,593 |  |  | 0,498 | 0,001 | -1,259 | -1,626 | 0,750 |  |  |  |
| WikiD15 | SklearnNmf | 0,777 | 0,799 | 0,667 | 0,760 |  |  | 0,662 | 0,082 | 0,279 | -1,158 | 0,810 | 0,104 | 46,514 | 0,152 |
| WikiD15 | SklearnPlsa | 0,740 | 0,759 | 0,638 | 0,731 |  |  | 0,652 | 0,075 | 0,149 | -1,161 | 0,814 | 0,131 | 8,636 | 0,152 |
| WikiD15 | SklearnBatchVbLda | 0,654 | 0,684 | 0,557 | 0,687 |  | 473,224 | 0,576 | 0,062 | 0,247 | -1,091 | 0,781 | 0,184 | 49,111 | 0,163 |
| WikiD15 | SklearnOnlineVbLda | 0,553 | 0,593 | 0,462 | 0,622 |  | 490,055 | 0,562 | 0,055 | 0,144 | -1,108 | 0,781 | 0,179 | 49,610 | 0,165 |
| WikiD15 | GensimBatchVbLda | 0,629 | 0,658 | 0,534 | 0,673 |  | 473,249 | 0,559 | 0,057 | 0,186 | -1,078 | 0,774 | 0,225 | 51,612 | 0,188 |
| WikiD15 | GensimOnlineVbLda | 0,581 | 0,610 | 0,484 | 0,636 |  | 478,184 | 0,577 | 0,062 | 0,237 | -1,106 | 0,786 | 0,165 | 48,539 | 0,155 |
| WikiD15 | MalletGibbsLda | 0,745 | 0,762 | 0,628 | 0,732 |  | 733,905 | 0,592 | 0,071 | 0,429 | -1,050 | 0,783 | 0,193 | 49,384 | 0,167 |
| WikiD17 | SklearnKmeans | 0,731 | 0,751 | 0,576 | 0,733 |  |  | 0,518 | 0,035 | -0,242 | -1,230 | 0,745 | 0,992 | 69,906 | 0,286 |
| WikiD17 | SklearnLsi | 0,593 | 0,631 | 0,456 | 0,615 |  |  | 0,446 | -0,030 | -1,950 | -1,983 | 0,722 |  |  |  |
| WikiD17 | SklearnNmf | 0,754 | 0,778 | 0,623 | 0,766 |  |  | 0,681 | 0,088 | 0,414 | -1,177 | 0,809 | 0,091 | 49,611 | 0,150 |
| WikiD17 | SklearnPlsa | 0,748 | 0,769 | 0,663 | 0,764 |  |  | 0,665 | 0,082 | 0,357 | -1,174 | 0,809 | 0,115 | 13,639 | 0,151 |
| WikiD17 | SklearnBatchVbLda | 0,636 | 0,671 | 0,559 | 0,706 |  | 436,660 | 0,582 | 0,067 | 0,354 | -1,118 | 0,774 | 0,171 | 51,538 | 0,161 |
| WikiD17 | SklearnOnlineVbLda | 0,532 | 0,565 | 0,456 | 0,630 |  | 454,271 | 0,559 | 0,055 | 0,148 | -1,144 | 0,771 | 0,163 | 51,425 | 0,159 |
| WikiD17 | GensimBatchVbLda | 0,624 | 0,656 | 0,551 | 0,698 |  | 440,133 | 0,553 | 0,054 | 0,148 | -1,137 | 0,763 | 0,212 | 54,560 | 0,186 |
| WikiD17 | GensimOnlineVbLda | 0,589 | 0,621 | 0,506 | 0,667 |  | 440,471 | 0,575 | 0,061 | 0,229 | -1,137 | 0,775 | 0,146 | 50,557 | 0,150 |
| WikiD17 | MalletGibbsLda | 0,740 | 0,759 | 0,628 | 0,743 |  | 681,404 | 0,604 | 0,076 | 0,506 | -1,116 | 0,777 | 0,179 | 51,432 | 0,166 |
| WikiD20 | SklearnKmeans | 0,691 | 0,725 | 0,548 | 0,713 |  |  | 0,522 | 0,035 | -0,263 | -1,477 | 0,734 | 0,993 | 76,334 | 0,290 |
| WikiD20 | SklearnLsi | 0,483 | 0,519 | 0,329 | 0,534 |  |  | 0,394 | -0,056 | -2,563 | -2,181 | 0,703 |  |  |  |
| WikiD20 | SklearnNmf | 0,703 | 0,744 | 0,586 | 0,712 |  |  | 0,684 | 0,097 | 0,621 | -1,163 | 0,798 | 0,088 | 48,738 | 0,147 |
| WikiD20 | SklearnPlsa | 0,709 | 0,724 | 0,597 | 0,718 |  |  | 0,670 | 0,088 | 0,468 | -1,185 | 0,801 | 0,109 | 22,564 | 0,146 |
| WikiD20 | SklearnBatchVbLda | 0,623 | 0,659 | 0,541 | 0,696 |  | 435,526 | 0,587 | 0,070 | 0,438 | -1,125 | 0,765 | 0,160 | 52,262 | 0,158 |
| WikiD20 | SklearnOnlineVbLda | 0,511 | 0,545 | 0,449 | 0,632 |  | 452,768 | 0,569 | 0,059 | 0,201 | -1,163 | 0,765 | 0,151 | 52,275 | 0,154 |
| WikiD20 | GensimBatchVbLda | 0,603 | 0,637 | 0,542 | 0,695 |  | 436,272 | 0,567 | 0,063 | 0,330 | -1,123 | 0,758 | 0,202 | 55,873 | 0,183 |
| WikiD20 | GensimOnlineVbLda | 0,531 | 0,561 | 0,474 | 0,650 |  | 441,411 | 0,580 | 0,065 | 0,324 | -1,151 | 0,770 | 0,137 | 51,638 | 0,146 |
| WikiD20 | MalletGibbsLda | 0,690 | 0,724 | 0,599 | 0,727 |  | 696,781 | 0,600 | 0,076 | 0,549 | -1,097 | 0,768 | 0,170 | 52,506 | 0,164 |
| WikiD23 | SklearnKmeans | 0,659 | 0,693 | 0,500 | 0,691 |  |  | 0,510 | 0,040 | -0,082 | -1,205 | 0,722 | 0,993 | 80,945 | 0,294 |
| WikiD23 | SklearnLsi | 0,546 | 0,583 | 0,411 | 0,588 |  |  | 0,391 | -0,067 | -2,664 | -2,246 | 0,687 |  |  |  |
| WikiD23 | SklearnNmf | 0,694 | 0,721 | 0,554 | 0,703 |  |  | 0,654 | 0,089 | 0,551 | -1,199 | 0,786 | 0,074 | 48,195 | 0,145 |
| WikiD23 | SklearnPlsa | 0,690 | 0,720 | 0,593 | 0,733 |  |  | 0,652 | 0,083 | 0,418 | -1,205 | 0,790 | 0,093 | 31,526 | 0,145 |
| WikiD23 | SklearnBatchVbLda | 0,605 | 0,635 | 0,527 | 0,694 |  | 408,691 | 0,578 | 0,068 | 0,416 | -1,142 | 0,755 | 0,143 | 52,757 | 0,155 |
| WikiD23 | SklearnOnlineVbLda | 0,484 | 0,519 | 0,430 | 0,625 |  | 426,585 | 0,559 | 0,058 | 0,229 | -1,169 | 0,755 | 0,127 | 52,216 | 0,148 |
| WikiD23 | GensimBatchVbLda | 0,581 | 0,613 | 0,515 | 0,683 |  | 410,802 | 0,555 | 0,060 | 0,309 | -1,134 | 0,746 | 0,181 | 56,487 | 0,182 |
| WikiD23 | GensimOnlineVbLda | 0,488 | 0,523 | 0,449 | 0,634 |  | 416,964 | 0,563 | 0,060 | 0,275 | -1,168 | 0,759 | 0,114 | 50,469 | 0,139 |
| WikiD23 | MalletGibbsLda | 0,683 | 0,706 | 0,581 | 0,723 |  | 672,555 | 0,585 | 0,072 | 0,493 | -1,112 | 0,755 | 0,155 | 52,940 | 0,162 |
| WikiD25 | SklearnKmeans | 0,658 | 0,688 | 0,507 | 0,691 |  |  | 0,513 | 0,038 | -0,094 | -1,154 | 0,728 | 0,993 | 84,698 | 0,297 |
| WikiD25 | SklearnLsi | 0,460 | 0,480 | 0,333 | 0,516 |  |  | 0,385 | -0,072 | -2,760 | -1,956 | 0,685 |  |  |  |
| WikiD25 | SklearnNmf | 0,627 | 0,668 | 0,502 | 0,668 |  |  | 0,656 | 0,085 | 0,531 | -1,219 | 0,788 | 0,075 | 50,596 | 0,144 |
| WikiD25 | SklearnPlsa | 0,691 | 0,705 | 0,584 | 0,720 |  |  | 0,658 | 0,085 | 0,481 | -1,231 | 0,796 | 0,097 | 36,693 | 0,142 |
| WikiD25 | SklearnBatchVbLda | 0,586 | 0,612 | 0,510 | 0,677 |  | 407,049 | 0,578 | 0,068 | 0,429 | -1,169 | 0,760 | 0,141 | 54,302 | 0,154 |
| WikiD25 | SklearnOnlineVbLda | 0,465 | 0,486 | 0,409 | 0,609 |  | 426,141 | 0,559 | 0,058 | 0,234 | -1,207 | 0,761 | 0,125 | 53,694 | 0,146 |
| WikiD25 | GensimBatchVbLda | 0,573 | 0,599 | 0,501 | 0,674 |  | 409,837 | 0,555 | 0,060 | 0,326 | -1,155 | 0,751 | 0,180 | 58,121 | 0,181 |
| WikiD25 | GensimOnlineVbLda | 0,477 | 0,501 | 0,434 | 0,628 |  | 415,274 | 0,566 | 0,059 | 0,225 | -1,208 | 0,764 | 0,111 | 54,014 | 0,136 |
| WikiD25 | MalletGibbsLda | 0,661 | 0,679 | 0,557 | 0,705 |  | 680,841 | 0,587 | 0,072 | 0,522 | -1,134 | 0,758 | 0,155 | 54,672 | 0,162 |
| WikiD27 | SklearnKmeans | 0,635 | 0,663 | 0,470 | 0,668 |  |  | 0,493 | 0,038 | -0,038 | -1,179 | 0,709 | 0,994 | 90,061 | 0,300 |
| WikiD27 | SklearnLsi | 0,508 | 0,523 | 0,359 | 0,525 |  |  | 0,343 | -0,068 | -2,453 | -1,907 | 0,662 |  |  |  |
| WikiD27 | SklearnNmf | 0,645 | 0,667 | 0,504 | 0,660 |  |  | 0,635 | 0,081 | 0,501 | -1,214 | 0,770 | 0,073 | 50,642 | 0,143 |
| WikiD27 | SklearnPlsa | 0,642 | 0,668 | 0,535 | 0,683 |  |  | 0,638 | 0,079 | 0,437 | -1,214 | 0,776 | 0,090 | 44,941 | 0,142 |
| WikiD27 | SklearnBatchVbLda | 0,580 | 0,607 | 0,488 | 0,661 |  | 418,493 | 0,564 | 0,065 | 0,422 | -1,147 | 0,742 | 0,137 | 54,981 | 0,154 |
| WikiD27 | SklearnOnlineVbLda | 0,433 | 0,460 | 0,368 | 0,576 |  | 438,192 | 0,549 | 0,056 | 0,244 | -1,188 | 0,743 | 0,120 | 55,067 | 0,144 |
| WikiD27 | GensimBatchVbLda | 0,543 | 0,571 | 0,465 | 0,647 |  | 422,423 | 0,546 | 0,059 | 0,325 | -1,149 | 0,733 | 0,177 | 59,434 | 0,181 |
| WikiD27 | GensimOnlineVbLda | 0,457 | 0,483 | 0,398 | 0,596 |  | 427,115 | 0,554 | 0,057 | 0,247 | -1,190 | 0,747 | 0,107 | 52,814 | 0,135 |
| WikiD27 | MalletGibbsLda | 0,648 | 0,674 | 0,540 | 0,691 |  | 723,001 | 0,577 | 0,072 | 0,552 | -1,120 | 0,743 | 0,154 | 56,018 | 0,162 |
| WikiD30 | SklearnKmeans | 0,603 | 0,631 | 0,433 | 0,641 |  |  | 0,486 | 0,033 | -0,094 | -1,206 | 0,699 | 0,994 | 93,829 | 0,304 |
| WikiD30 | SklearnLsi | 0,383 | 0,406 | 0,232 | 0,422 |  |  | 0,323 | -0,090 | -2,934 | -2,075 | 0,647 |  |  |  |
| WikiD30 | SklearnNmf | 0,581 | 0,611 | 0,431 | 0,611 |  |  | 0,611 | 0,071 | 0,371 | -1,260 | 0,758 | 0,070 | 50,285 | 0,143 |
| WikiD30 | SklearnPlsa | 0,556 | 0,580 | 0,453 | 0,622 |  |  | 0,613 | 0,070 | 0,310 | -1,256 | 0,768 | 0,084 | 57,715 | 0,136 |
| WikiD30 | SklearnBatchVbLda | 0,535 | 0,561 | 0,432 | 0,623 |  | 404,776 | 0,555 | 0,061 | 0,374 | -1,177 | 0,734 | 0,131 | 54,728 | 0,151 |
| WikiD30 | SklearnOnlineVbLda | 0,398 | 0,423 | 0,328 | 0,549 |  | 421,856 | 0,541 | 0,052 | 0,207 | -1,219 | 0,737 | 0,109 | 54,440 | 0,140 |
| WikiD30 | GensimBatchVbLda | 0,505 | 0,535 | 0,419 | 0,615 |  | 409,398 | 0,536 | 0,054 | 0,281 | -1,166 | 0,725 | 0,173 | 60,362 | 0,179 |
| WikiD30 | GensimOnlineVbLda | 0,424 | 0,444 | 0,357 | 0,568 |  | 411,448 | 0,545 | 0,053 | 0,194 | -1,226 | 0,741 | 0,096 | 52,559 | 0,130 |
| WikiD30 | MalletGibbsLda | 0,613 | 0,631 | 0,483 | 0,651 |  | 716,479 | 0,570 | 0,068 | 0,505 | -1,142 | 0,735 | 0,148 | 55,945 | 0,159 |
| WikiD35 | SklearnKmeans | 0,579 | 0,609 | 0,405 | 0,628 |  |  | 0,491 | 0,030 | -0,212 | -1,218 | 0,683 | 0,993 | 94,005 | 0,303 |
| WikiD35 | SklearnLsi | 0,390 | 0,433 | 0,251 | 0,441 |  |  | 0,292 | -0,107 | -3,280 | -2,133 | 0,620 |  |  |  |
| WikiD35 | SklearnNmf | 0,567 | 0,611 | 0,424 | 0,619 |  |  | 0,612 | 0,074 | 0,460 | -1,275 | 0,740 | 0,062 | 47,504 | 0,139 |
| WikiD35 | SklearnPlsa | 0,589 | 0,618 | 0,472 | 0,647 |  |  | 0,608 | 0,074 | 0,440 | -1,244 | 0,749 | 0,070 | 94,231 | 0,133 |
| WikiD35 | SklearnBatchVbLda | 0,522 | 0,548 | 0,413 | 0,617 |  | 395,580 | 0,559 | 0,066 | 0,472 | -1,172 | 0,720 | 0,119 | 52,601 | 0,147 |
| WikiD35 | SklearnOnlineVbLda | 0,386 | 0,407 | 0,304 | 0,540 |  | 415,746 | 0,531 | 0,053 | 0,259 | -1,222 | 0,718 | 0,096 | 54,200 | 0,134 |
| WikiD35 | GensimBatchVbLda | 0,507 | 0,532 | 0,405 | 0,616 |  | 400,798 | 0,539 | 0,059 | 0,374 | -1,169 | 0,710 | 0,157 | 57,946 | 0,175 |
| WikiD35 | GensimOnlineVbLda | 0,399 | 0,420 | 0,323 | 0,553 |  | 405,844 | 0,535 | 0,051 | 0,149 | -1,234 | 0,723 | 0,082 | 55,101 | 0,124 |
| WikiD35 | MalletGibbsLda | 0,615 | 0,631 | 0,470 | 0,649 |  | 716,821 | 0,573 | 0,072 | 0,568 | -1,146 | 0,721 | 0,136 | 54,008 | 0,156 |
| WikiD40 | SklearnKmeans | 0,528 | 0,559 | 0,360 | 0,599 |  |  | 0,489 | 0,031 | -0,174 | -1,232 | 0,673 | 0,994 | 101,662 | 0,306 |
| WikiD40 | SklearnLsi | 0,326 | 0,354 | 0,228 | 0,424 |  |  | 0,311 | -0,076 | -2,451 | -2,002 | 0,615 |  |  |  |
| WikiD40 | SklearnNmf | 0,505 | 0,532 | 0,365 | 0,572 |  |  | 0,590 | 0,069 | 0,373 | -1,315 | 0,726 | 0,054 | 46,001 | 0,136 |
| WikiD40 | SklearnPlsa | 0,503 | 0,525 | 0,396 | 0,596 |  |  | 0,611 | 0,077 | 0,504 | -1,279 | 0,745 | 0,058 | 129,757 | 0,126 |
| WikiD40 | SklearnBatchVbLda | 0,493 | 0,517 | 0,387 | 0,600 |  | 388,243 | 0,559 | 0,068 | 0,517 | -1,182 | 0,712 | 0,108 | 52,522 | 0,143 |
| WikiD40 | SklearnOnlineVbLda | 0,342 | 0,363 | 0,279 | 0,518 |  | 407,596 | 0,538 | 0,055 | 0,285 | -1,239 | 0,713 | 0,086 | 54,381 | 0,130 |
| WikiD40 | GensimBatchVbLda | 0,462 | 0,485 | 0,364 | 0,587 |  | 395,551 | 0,538 | 0,059 | 0,389 | -1,189 | 0,702 | 0,145 | 58,940 | 0,172 |
| WikiD40 | GensimOnlineVbLda | 0,379 | 0,396 | 0,299 | 0,535 |  | 395,445 | 0,543 | 0,054 | 0,212 | -1,255 | 0,718 | 0,074 | 54,254 | 0,121 |
| WikiD40 | MalletGibbsLda | 0,566 | 0,582 | 0,424 | 0,616 |  | 725,951 | 0,574 | 0,074 | 0,626 | -1,139 | 0,714 | 0,126 | 54,269 | 0,152 |
| WikiD45 | SklearnKmeans | 0,501 | 0,532 | 0,338 | 0,578 |  |  | 0,477 | 0,036 | 0,048 | -1,187 | 0,657 | 0,995 | 109,613 | 0,312 |
| WikiD45 | SklearnLsi | 0,312 | 0,341 | 0,217 | 0,401 |  |  | 0,289 | -0,077 | -2,329 | -1,879 | 0,603 |  |  |  |
| WikiD45 | SklearnNmf | 0,452 | 0,485 | 0,318 | 0,528 |  |  | 0,567 | 0,063 | 0,357 | -1,349 | 0,717 | 0,046 | 45,563 | 0,134 |
| WikiD45 | SklearnPlsa | 0,447 | 0,471 | 0,357 | 0,577 |  |  | 0,576 | 0,066 | 0,363 | -1,309 | 0,730 | 0,047 | 184,496 | 0,120 |
| WikiD45 | SklearnBatchVbLda | 0,467 | 0,495 | 0,365 | 0,588 |  | 377,963 | 0,543 | 0,063 | 0,483 | -1,215 | 0,699 | 0,100 | 52,315 | 0,142 |
| WikiD45 | SklearnOnlineVbLda | 0,315 | 0,333 | 0,259 | 0,506 |  | 398,990 | 0,516 | 0,049 | 0,217 | -1,268 | 0,698 | 0,086 | 63,943 | 0,130 |
| WikiD45 | GensimBatchVbLda | 0,442 | 0,469 | 0,354 | 0,585 |  | 384,225 | 0,522 | 0,056 | 0,378 | -1,210 | 0,688 | 0,136 | 59,900 | 0,171 |
| WikiD45 | GensimOnlineVbLda | 0,333 | 0,353 | 0,268 | 0,514 |  | 386,199 | 0,517 | 0,046 | 0,114 | -1,294 | 0,704 | 0,068 | 65,759 | 0,118 |
| WikiD45 | MalletGibbsLda | 0,560 | 0,578 | 0,418 | 0,619 |  | 718,175 | 0,560 | 0,070 | 0,594 | -1,172 | 0,701 | 0,121 | 54,712 | 0,152 |
| WikiD50 | SklearnKmeans | 0,482 | 0,506 | 0,316 | 0,559 |  |  | 0,474 | 0,030 | -0,138 | -1,336 | 0,653 | 0,995 | 113,052 | 0,315 |
| WikiD50 | SklearnLsi | 0,280 | 0,304 | 0,180 | 0,363 |  |  | 0,272 | -0,079 | -2,268 | -1,917 | 0,587 |  |  |  |
| WikiD50 | SklearnNmf | 0,448 | 0,470 | 0,307 | 0,519 |  |  | 0,564 | 0,062 | 0,358 | -1,364 | 0,708 | 0,040 | 43,869 | 0,130 |
| WikiD50 | SklearnPlsa | 0,449 | 0,464 | 0,348 | 0,556 |  |  | 0,574 | 0,066 | 0,395 | -1,335 | 0,723 | 0,039 | 248,598 | 0,117 |
| WikiD50 | SklearnBatchVbLda | 0,435 | 0,459 | 0,338 | 0,564 |  | 369,485 | 0,538 | 0,061 | 0,463 | -1,244 | 0,692 | 0,089 | 51,224 | 0,138 |
| WikiD50 | SklearnOnlineVbLda | 0,299 | 0,317 | 0,235 | 0,487 |  | 391,411 | 0,510 | 0,047 | 0,209 | -1,302 | 0,692 | 0,086 | 76,509 | 0,129 |
| WikiD50 | GensimBatchVbLda | 0,429 | 0,452 | 0,337 | 0,569 |  | 375,719 | 0,519 | 0,055 | 0,382 | -1,230 | 0,680 | 0,125 | 58,694 | 0,169 |
| WikiD50 | GensimOnlineVbLda | 0,315 | 0,333 | 0,245 | 0,493 |  | 376,717 | 0,515 | 0,045 | 0,112 | -1,326 | 0,700 | 0,060 | 68,892 | 0,115 |
| WikiD50 | MalletGibbsLda | 0,526 | 0,540 | 0,384 | 0,588 |  | 709,783 | 0,556 | 0,068 | 0,581 | -1,198 | 0,692 | 0,113 | 54,132 | 0,149 |
| WikiD55 | SklearnKmeans | 0,463 | 0,481 | 0,296 | 0,536 |  | 0,002 | 0,468 | 0,033 | -0,104 | -1,253 | 0,648 | 0,997 | 129,575 | 0,339 |
| WikiD55 | SklearnLsi | 0,276 | 0,293 | 0,175 | 0,356 |  | 0,009 | 0,272 | -0,074 | -2,126 | -1,989 | 0,600 |  |  |  |
| WikiD55 | SklearnNmf | 0,427 | 0,440 | 0,278 | 0,489 |  | 0,005 | 0,557 | 0,061 | 0,354 | -1,436 | 0,719 | 0,033 | 39,537 | 0,129 |
| WikiD55 | SklearnPlsa | 0,406 | 0,423 | 0,319 | 0,530 |  | 0,005 | 0,559 | 0,070 | 0,379 | -1,661 | 0,744 | 0,012 | 696,385 | 0,090 |
| WikiD55 | SklearnBatchVbLda | 0,420 | 0,440 | 0,323 | 0,549 |  | 365,694 | 0,534 | 0,064 | 0,462 | -1,301 | 0,706 | 0,078 | 48,101 | 0,136 |
| WikiD55 | SklearnOnlineVbLda | 0,277 | 0,295 | 0,215 | 0,477 |  | 389,038 | 0,504 | 0,055 | 0,216 | -1,378 | 0,711 | 0,072 | 76,073 | 0,122 |
| WikiD55 | GensimBatchVbLda | 0,404 | 0,430 | 0,319 | 0,553 |  | 372,934 | 0,512 | 0,062 | 0,377 | -1,284 | 0,692 | 0,113 | 56,957 | 0,168 |
| WikiD55 | GensimOnlineVbLda | 0,291 | 0,314 | 0,233 | 0,485 |  | 373,275 | 0,501 | 0,043 | -0,005 | -1,405 | 0,720 | 0,051 | 80,679 | 0,110 |
| WikiD55 | MalletGibbsLda | 0,505 | 0,520 | 0,357 | 0,577 |  | 713,866 | 0,552 | 0,072 | 0,568 | -1,265 | 0,702 | 0,112 | 54,834 | 0,151 |
| WikiD60 | SklearnKmeans | 0,425 | 0,455 | 0,268 | 0,512 |  |  | 0,457 | 0,028 | -0,088 | -1,314 | 0,634 | 0,995 | 124,066 | 0,319 |
| WikiD60 | SklearnLsi | 0,260 | 0,279 | 0,159 | 0,335 |  |  | 0,259 | -0,072 | -1,995 | -1,859 | 0,571 |  |  |  |
| WikiD60 | SklearnNmf | 0,389 | 0,404 | 0,244 | 0,459 |  |  | 0,541 | 0,057 | 0,331 | -1,403 | 0,691 | 0,033 | 42,014 | 0,127 |
| WikiD60 | SklearnPlsa | 0,357 | 0,374 | 0,271 | 0,491 |  |  | 0,540 | 0,058 | 0,347 | -1,346 | 0,703 | 0,026 | 427,255 | 0,109 |
| WikiD60 | SklearnBatchVbLda | 0,397 | 0,419 | 0,292 | 0,529 |  | 361,895 | 0,522 | 0,058 | 0,447 | -1,255 | 0,676 | 0,073 | 48,878 | 0,134 |
| WikiD60 | SklearnOnlineVbLda | 0,244 | 0,259 | 0,192 | 0,448 |  | 386,661 | 0,492 | 0,044 | 0,216 | -1,310 | 0,675 | 0,086 | 103,889 | 0,128 |
| WikiD60 | GensimBatchVbLda | 0,374 | 0,398 | 0,281 | 0,525 |  | 370,137 | 0,501 | 0,051 | 0,359 | -1,247 | 0,662 | 0,109 | 59,276 | 0,165 |
| WikiD60 | GensimOnlineVbLda | 0,260 | 0,278 | 0,203 | 0,457 |  | 369,821 | 0,482 | 0,032 | -0,138 | -1,389 | 0,685 | 0,059 | 118,837 | 0,113 |
| WikiD60 | MalletGibbsLda | 0,468 | 0,485 | 0,329 | 0,553 |  | 717,935 | 0,541 | 0,065 | 0,555 | -1,214 | 0,676 | 0,097 | 53,663 | 0,144 |
| WikiD65 | SklearnKmeans | 0,417 | 0,441 | 0,253 | 0,501 |  | 0,002 | 0,454 | 0,037 | -0,059 | -1,163 | 0,634 | 0,997 | 136,339 | 0,340 |
| WikiD65 | SklearnLsi | 0,255 | 0,271 | 0,155 | 0,317 |  | 0,006 | 0,242 | -0,071 | -1,947 | -1,963 | 0,572 |  |  |  |
| WikiD65 | SklearnNmf | 0,373 | 0,386 | 0,224 | 0,446 |  | 0,004 | 0,538 | 0,057 | 0,352 | -1,491 | 0,704 | 0,027 | 38,091 | 0,124 |
| WikiD65 | SklearnPlsa | 0,347 | 0,364 | 0,266 | 0,490 |  | 0,005 | 0,539 | 0,057 | 0,368 | -1,703 | 0,725 | 0,008 | 1049,848 | 0,088 |
| WikiD65 | SklearnBatchVbLda | 0,390 | 0,408 | 0,283 | 0,523 |  | 356,424 | 0,516 | 0,064 | 0,452 | -1,313 | 0,689 | 0,064 | 44,047 | 0,131 |
| WikiD65 | SklearnOnlineVbLda | 0,230 | 0,244 | 0,184 | 0,442 |  | 383,545 | 0,480 | 0,046 | 0,183 | -1,407 | 0,691 | 0,068 | 100,366 | 0,118 |
| WikiD65 | GensimBatchVbLda | 0,367 | 0,393 | 0,274 | 0,522 |  | 364,353 | 0,504 | 0,060 | 0,374 | -1,288 | 0,672 | 0,101 | 55,540 | 0,165 |
| WikiD65 | GensimOnlineVbLda | 0,242 | 0,259 | 0,191 | 0,449 |  | 364,706 | 0,489 | 0,037 | -0,008 | -1,453 | 0,698 | 0,045 | 98,329 | 0,106 |
| WikiD65 | MalletGibbsLda | 0,461 | 0,472 | 0,326 | 0,543 |  | 713,279 | 0,535 | 0,072 | 0,554 | -1,275 | 0,685 | 0,100 | 53,187 | 0,147 |
| WikiD70 | SklearnKmeans | 0,398 | 0,425 | 0,234 | 0,482 |  |  | 0,442 | 0,026 | -0,057 | -1,182 | 0,622 | 0,997 | 142,632 | 0,341 |
| WikiD70 | SklearnLsi | 0,241 | 0,260 | 0,145 | 0,303 |  |  | 0,229 | -0,080 | -1,930 | -1,898 | 0,563 |  |  |  |
| WikiD70 | SklearnNmf | 0,358 | 0,366 | 0,204 | 0,432 |  |  | 0,524 | 0,053 | 0,356 | -1,492 | 0,696 | 0,025 | 38,773 | 0,123 |
| WikiD70 | SklearnPlsa | 0,340 | 0,351 | 0,255 | 0,476 |  |  | 0,527 | 0,051 | 0,362 | -1,726 | 0,716 | 0,006 | 1223,228 | 0,087 |
| WikiD70 | SklearnBatchVbLda | 0,375 | 0,397 | 0,265 | 0,509 |  | 353,682 | 0,506 | 0,055 | 0,443 | -1,321 | 0,679 | 0,059 | 43,762 | 0,129 |
| WikiD70 | SklearnOnlineVbLda | 0,215 | 0,226 | 0,166 | 0,427 |  | 381,986 | 0,469 | 0,037 | 0,156 | -1,415 | 0,682 | 0,067 | 111,692 | 0,118 |
| WikiD70 | GensimBatchVbLda | 0,359 | 0,385 | 0,264 | 0,512 |  | 361,460 | 0,498 | 0,050 | 0,376 | -1,294 | 0,662 | 0,096 | 56,646 | 0,164 |
| WikiD70 | GensimOnlineVbLda | 0,229 | 0,240 | 0,173 | 0,433 |  | 362,136 | 0,479 | 0,032 | 0,050 | -1,465 | 0,695 | 0,038 | 93,291 | 0,104 |
| WikiD70 | MalletGibbsLda | 0,445 | 0,455 | 0,305 | 0,532 |  | 710,943 | 0,529 | 0,064 | 0,549 | -1,288 | 0,676 | 0,093 | 52,309 | 0,145 |
| WikiD75 | SklearnKmeans | 0,395 | 0,423 | 0,234 | 0,481 |  |  | 0,447 | 0,028 | -0,041 | -1,296 | 0,617 | 0,996 | 135,157 | 0,322 |
| WikiD75 | SklearnLsi | 0,236 | 0,253 | 0,136 | 0,294 |  |  | 0,225 | -0,075 | -1,902 | -1,933 | 0,548 |  |  |  |
| WikiD75 | SklearnNmf | 0,345 | 0,365 | 0,203 | 0,421 |  |  | 0,523 | 0,054 | 0,363 | -1,432 | 0,669 | 0,026 | 41,873 | 0,123 |
| WikiD75 | SklearnPlsa | 0,334 | 0,350 | 0,255 | 0,478 |  |  | 0,520 | 0,055 | 0,370 | -1,351 | 0,685 | 0,017 | 756,197 | 0,104 |
| WikiD75 | SklearnBatchVbLda | 0,374 | 0,395 | 0,266 | 0,508 |  | 350,944 | 0,508 | 0,056 | 0,454 | -1,273 | 0,658 | 0,059 | 45,168 | 0,129 |
| WikiD75 | SklearnOnlineVbLda | 0,212 | 0,223 | 0,166 | 0,429 |  | 380,428 | 0,467 | 0,038 | 0,147 | -1,346 | 0,654 | 0,103 | 170,902 | 0,132 |
| WikiD75 | GensimBatchVbLda | 0,359 | 0,380 | 0,259 | 0,508 |  | 358,568 | 0,492 | 0,050 | 0,388 | -1,251 | 0,646 | 0,092 | 57,692 | 0,162 |
| WikiD75 | GensimOnlineVbLda | 0,223 | 0,235 | 0,174 | 0,433 |  | 359,584 | 0,476 | 0,037 | 0,120 | -1,369 | 0,665 | 0,060 | 188,771 | 0,111 |
| WikiD75 | MalletGibbsLda | 0,438 | 0,457 | 0,302 | 0,531 |  | 708,614 | 0,527 | 0,062 | 0,546 | -1,232 | 0,656 | 0,086 | 52,621 | 0,141 |
| WikiE2 | SklearnKmeans | 1,000 | 1,000 | 1,000 | 1,000 |  |  | 0,649 | -0,012 | -1,413 | -1,754 | 0,977 | 0,989 | 23,142 | 0,160 |
| WikiE2 | SklearnLsi | 0,727 | 0,727 | 0,234 | 0,331 |  |  | 0,458 | -0,059 | -2,504 | -1,981 | 0,958 |  |  |  |
| WikiE2 | SklearnNmf | 1,000 | 1,000 | 1,000 | 1,000 |  |  | 0,726 | 0,049 | -0,221 | -0,672 | 0,982 | 0,120 | 30,317 | 0,107 |
| WikiE2 | SklearnPlsa | 1,000 | 1,000 | 1,000 | 1,000 |  |  | 0,698 | 0,045 | -0,225 | -0,657 | 0,981 | 0,126 | 2,440 | 0,105 |
| WikiE2 | SklearnBatchVbLda | 1,000 | 1,000 | 1,000 | 1,000 |  | 622,130 | 0,656 | 0,037 | -0,083 | -0,666 | 0,978 | 0,162 | 29,203 | 0,127 |
| WikiE2 | SklearnOnlineVbLda | 0,946 | 0,946 | 0,849 | 0,836 |  | 660,135 | 0,600 | 0,006 | -0,882 | -1,144 | 0,975 | 0,286 | 29,760 | 0,153 |
| WikiE2 | GensimBatchVbLda | 0,998 | 0,998 | 0,994 | 0,990 |  | 612,459 | 0,653 | 0,035 | -0,169 | -0,694 | 0,978 | 0,176 | 29,291 | 0,131 |
| WikiE2 | GensimOnlineVbLda | 0,984 | 0,984 | 0,946 | 0,932 |  | 625,749 | 0,631 | 0,020 | -0,556 | -0,932 | 0,976 | 0,247 | 29,621 | 0,148 |
| WikiE2 | MalletGibbsLda | 1,000 | 1,000 | 1,000 | 1,000 |  | 628,655 | 0,657 | 0,037 | -0,106 | -0,670 | 0,978 | 0,149 | 28,944 | 0,112 |
| WikiE3 | SklearnKmeans | 1,000 | 1,000 | 1,000 | 1,000 |  |  | 0,675 | 0,028 | -0,980 | -1,729 | 0,947 | 0,984 | 29,283 | 0,186 |
| WikiE3 | SklearnLsi | 0,793 | 0,793 | 0,608 | 0,722 |  |  | 0,672 | -0,025 | -2,379 | -2,175 | 0,928 |  |  |  |
| WikiE3 | SklearnNmf | 1,000 | 1,000 | 1,000 | 1,000 |  |  | 0,798 | 0,084 | -0,109 | -0,739 | 0,968 | 0,100 | 33,989 | 0,123 |
| WikiE3 | SklearnPlsa | 1,000 | 1,000 | 1,000 | 1,000 |  |  | 0,785 | 0,078 | -0,231 | -0,746 | 0,969 | 0,109 | 1,498 | 0,122 |
| WikiE3 | SklearnBatchVbLda | 0,959 | 0,972 | 0,957 | 0,972 |  | 440,702 | 0,716 | 0,067 | 0,001 | -0,813 | 0,953 | 0,162 | 33,686 | 0,142 |
| WikiE3 | SklearnOnlineVbLda | 0,914 | 0,943 | 0,909 | 0,939 |  | 452,250 | 0,696 | 0,059 | -0,181 | -0,935 | 0,951 | 0,193 | 34,044 | 0,151 |
| WikiE3 | GensimBatchVbLda | 0,998 | 0,998 | 0,995 | 0,994 |  | 426,230 | 0,724 | 0,069 | 0,059 | -0,770 | 0,954 | 0,162 | 33,597 | 0,144 |
| WikiE3 | GensimOnlineVbLda | 0,867 | 0,909 | 0,852 | 0,903 |  | 389,182 | 0,662 | 0,030 | -0,887 | -1,088 | 0,977 | 0,217 | 34,325 | 0,154 |
| WikiE3 | MalletGibbsLda | 1,000 | 1,000 | 1,000 | 1,000 |  | 442,437 | 0,727 | 0,070 | 0,077 | -0,759 | 0,952 | 0,147 | 33,296 | 0,132 |
| WikiE5 | SklearnKmeans | 0,813 | 0,813 | 0,760 | 0,840 |  |  | 0,702 | 0,033 | -1,135 | -1,979 | 0,940 | 0,996 | 42,125 | 0,246 |
| WikiE5 | SklearnLsi | 0,813 | 0,813 | 0,765 | 0,847 |  |  | 0,749 | 0,058 | -1,036 | -1,459 | 0,955 |  |  |  |
| WikiE5 | SklearnNmf | 0,804 | 0,804 | 0,752 | 0,830 |  |  | 0,808 | 0,094 | -0,093 | -0,844 | 0,970 | 0,115 | 38,425 | 0,140 |
| WikiE5 | SklearnPlsa | 0,814 | 0,814 | 0,756 | 0,834 |  |  | 0,793 | 0,081 | -0,304 | -0,831 | 0,969 | 0,130 | 0,466 | 0,138 |
| WikiE5 | SklearnBatchVbLda | 0,723 | 0,753 | 0,694 | 0,792 |  | 356,289 | 0,719 | 0,072 | -0,117 | -0,969 | 0,950 | 0,189 | 39,194 | 0,155 |
| WikiE5 | SklearnOnlineVbLda | 0,742 | 0,784 | 0,740 | 0,851 |  | 362,005 | 0,725 | 0,067 | -0,289 | -1,013 | 0,947 | 0,194 | 40,352 | 0,163 |
| WikiE5 | GensimBatchVbLda | 0,731 | 0,755 | 0,682 | 0,779 |  | 355,206 | 0,703 | 0,063 | -0,302 | -1,139 | 0,946 | 0,219 | 40,132 | 0,166 |
| WikiE5 | GensimOnlineVbLda | 0,762 | 0,781 | 0,738 | 0,838 |  | 349,542 | 0,732 | 0,072 | -0,195 | -0,945 | 0,950 | 0,183 | 39,908 | 0,159 |
| WikiE5 | MalletGibbsLda | 0,803 | 0,811 | 0,752 | 0,836 |  | 450,055 | 0,764 | 0,101 | 0,471 | -0,772 | 0,908 | 0,169 | 39,416 | 0,146 |
| WikiE7 | SklearnKmeans | 0,868 | 0,868 | 0,830 | 0,896 |  |  | 0,660 | 0,046 | -0,804 | -1,796 | 0,935 | 0,997 | 48,922 | 0,271 |
| WikiE7 | SklearnLsi | 0,677 | 0,720 | 0,584 | 0,765 |  |  | 0,663 | 0,048 | -1,154 | -1,632 | 0,927 |  |  |  |
| WikiE7 | SklearnNmf | 0,878 | 0,878 | 0,827 | 0,891 |  |  | 0,791 | 0,106 | 0,157 | -0,909 | 0,961 | 0,118 | 40,871 | 0,152 |
| WikiE7 | SklearnPlsa | 0,850 | 0,850 | 0,818 | 0,884 |  |  | 0,777 | 0,084 | -0,267 | -0,922 | 0,959 | 0,134 | 1,068 | 0,152 |
| WikiE7 | SklearnBatchVbLda | 0,752 | 0,781 | 0,739 | 0,838 |  | 368,277 | 0,677 | 0,072 | -0,031 | -1,057 | 0,934 | 0,194 | 41,828 | 0,164 |
| WikiE7 | SklearnOnlineVbLda | 0,716 | 0,766 | 0,700 | 0,817 |  | 375,870 | 0,664 | 0,067 | -0,127 | -1,061 | 0,931 | 0,216 | 42,703 | 0,174 |
| WikiE7 | GensimBatchVbLda | 0,725 | 0,761 | 0,707 | 0,816 |  | 363,588 | 0,660 | 0,065 | -0,197 | -1,124 | 0,930 | 0,228 | 42,982 | 0,180 |
| WikiE7 | GensimOnlineVbLda | 0,709 | 0,754 | 0,710 | 0,830 |  | 364,356 | 0,670 | 0,073 | 0,035 | -1,000 | 0,933 | 0,205 | 42,383 | 0,170 |
| WikiE7 | MalletGibbsLda | 0,814 | 0,820 | 0,778 | 0,855 |  | 492,727 | 0,726 | 0,105 | 0,556 | -0,817 | 0,885 | 0,178 | 42,232 | 0,159 |
| WikiE10 | SklearnKmeans | 0,862 | 0,865 | 0,798 | 0,854 |  |  | 0,630 | 0,045 | -0,649 | -2,155 | 0,895 | 0,997 | 55,237 | 0,290 |
| WikiE10 | SklearnLsi | 0,737 | 0,779 | 0,613 | 0,773 |  |  | 0,594 | 0,003 | -1,822 | -1,930 | 0,888 |  |  |  |
| WikiE10 | SklearnNmf | 0,866 | 0,866 | 0,801 | 0,857 |  |  | 0,786 | 0,109 | 0,359 | -0,958 | 0,935 | 0,100 | 42,004 | 0,154 |
| WikiE10 | SklearnPlsa | 0,866 | 0,868 | 0,824 | 0,873 |  |  | 0,776 | 0,102 | 0,230 | -0,971 | 0,935 | 0,114 | 3,885 | 0,154 |
| WikiE10 | SklearnBatchVbLda | 0,733 | 0,768 | 0,706 | 0,808 |  | 325,065 | 0,662 | 0,078 | 0,183 | -1,119 | 0,905 | 0,172 | 43,414 | 0,165 |
| WikiE10 | SklearnOnlineVbLda | 0,674 | 0,710 | 0,635 | 0,775 |  | 334,736 | 0,617 | 0,056 | -0,154 | -1,145 | 0,897 | 0,183 | 44,180 | 0,170 |
| WikiE10 | GensimBatchVbLda | 0,703 | 0,732 | 0,673 | 0,786 |  | 325,042 | 0,626 | 0,063 | -0,087 | -1,195 | 0,897 | 0,208 | 44,836 | 0,183 |
| WikiE10 | GensimOnlineVbLda | 0,734 | 0,762 | 0,692 | 0,811 |  | 321,891 | 0,656 | 0,075 | 0,172 | -1,069 | 0,904 | 0,173 | 43,683 | 0,165 |
| WikiE10 | MalletGibbsLda | 0,778 | 0,804 | 0,749 | 0,832 |  | 481,470 | 0,712 | 0,104 | 0,611 | -0,968 | 0,858 | 0,164 | 44,319 | 0,164 |
| WikiE13 | SklearnKmeans | 0,806 | 0,813 | 0,686 | 0,799 |  |  | 0,569 | 0,041 | -0,395 | -1,531 | 0,851 | 0,995 | 62,687 | 0,299 |
| WikiE13 | SklearnLsi | 0,733 | 0,746 | 0,608 | 0,746 |  |  | 0,508 | -0,034 | -2,462 | -2,372 | 0,828 |  |  |  |
| WikiE13 | SklearnNmf | 0,838 | 0,838 | 0,729 | 0,817 |  |  | 0,725 | 0,097 | 0,396 | -1,041 | 0,902 | 0,087 | 43,309 | 0,158 |
| WikiE13 | SklearnPlsa | 0,859 | 0,859 | 0,786 | 0,843 |  |  | 0,730 | 0,091 | 0,177 | -1,116 | 0,912 | 0,104 | 7,055 | 0,154 |
| WikiE13 | SklearnBatchVbLda | 0,683 | 0,716 | 0,648 | 0,767 |  | 306,566 | 0,626 | 0,071 | 0,208 | -1,125 | 0,875 | 0,161 | 45,462 | 0,164 |
| WikiE13 | SklearnOnlineVbLda | 0,620 | 0,654 | 0,568 | 0,734 |  | 320,840 | 0,587 | 0,051 | -0,125 | -1,154 | 0,868 | 0,155 | 46,394 | 0,160 |
| WikiE13 | GensimBatchVbLda | 0,682 | 0,712 | 0,635 | 0,759 |  | 303,503 | 0,605 | 0,063 | 0,070 | -1,153 | 0,867 | 0,189 | 46,867 | 0,185 |
| WikiE13 | GensimOnlineVbLda | 0,650 | 0,689 | 0,611 | 0,754 |  | 309,303 | 0,603 | 0,059 | 0,025 | -1,132 | 0,872 | 0,145 | 44,676 | 0,155 |
| WikiE13 | MalletGibbsLda | 0,766 | 0,780 | 0,709 | 0,801 |  | 470,199 | 0,676 | 0,097 | 0,616 | -1,001 | 0,830 | 0,157 | 46,332 | 0,165 |
| WikiE15 | SklearnKmeans | 0,778 | 0,790 | 0,639 | 0,773 |  |  | 0,552 | 0,037 | -0,395 | -1,679 | 0,831 | 0,996 | 68,307 | 0,304 |
| WikiE15 | SklearnLsi | 0,743 | 0,757 | 0,613 | 0,732 |  |  | 0,450 | -0,089 | -3,676 | -3,060 | 0,788 |  |  |  |
| WikiE15 | SklearnNmf | 0,770 | 0,800 | 0,679 | 0,797 |  |  | 0,679 | 0,076 | 0,097 | -1,104 | 0,880 | 0,080 | 45,566 | 0,156 |
| WikiE15 | SklearnPlsa | 0,773 | 0,804 | 0,705 | 0,807 |  |  | 0,673 | 0,064 | -0,106 | -1,167 | 0,884 | 0,105 | 11,534 | 0,153 |
| WikiE15 | SklearnBatchVbLda | 0,660 | 0,698 | 0,602 | 0,737 |  | 311,677 | 0,609 | 0,065 | 0,210 | -1,148 | 0,858 | 0,154 | 47,091 | 0,162 |
| WikiE15 | SklearnOnlineVbLda | 0,578 | 0,608 | 0,502 | 0,678 |  | 329,235 | 0,565 | 0,041 | -0,244 | -1,220 | 0,851 | 0,138 | 47,027 | 0,153 |
| WikiE15 | GensimBatchVbLda | 0,660 | 0,688 | 0,589 | 0,729 |  | 311,824 | 0,575 | 0,052 | -0,042 | -1,192 | 0,849 | 0,188 | 49,410 | 0,184 |
| WikiE15 | GensimOnlineVbLda | 0,630 | 0,662 | 0,557 | 0,712 |  | 318,374 | 0,591 | 0,054 | 0,002 | -1,199 | 0,856 | 0,133 | 46,143 | 0,149 |
| WikiE15 | MalletGibbsLda | 0,752 | 0,767 | 0,677 | 0,779 |  | 510,093 | 0,652 | 0,086 | 0,472 | -1,079 | 0,817 | 0,153 | 48,760 | 0,164 |
| WikiE17 | SklearnKmeans | 0,750 | 0,768 | 0,612 | 0,767 |  |  | 0,542 | 0,033 | -0,414 | -1,506 | 0,828 | 0,996 | 67,382 | 0,309 |
| WikiE17 | SklearnLsi | 0,709 | 0,736 | 0,551 | 0,697 |  |  | 0,429 | -0,071 | -3,193 | -2,461 | 0,792 |  |  |  |
| WikiE17 | SklearnNmf | 0,805 | 0,805 | 0,671 | 0,783 |  |  | 0,685 | 0,085 | 0,359 | -1,155 | 0,882 | 0,078 | 41,969 | 0,156 |
| WikiE17 | SklearnPlsa | 0,844 | 0,844 | 0,739 | 0,819 |  |  | 0,685 | 0,084 | 0,317 | -1,165 | 0,883 | 0,097 | 16,960 | 0,152 |
| WikiE17 | SklearnBatchVbLda | 0,654 | 0,688 | 0,598 | 0,741 |  | 310,419 | 0,602 | 0,065 | 0,246 | -1,144 | 0,854 | 0,144 | 44,234 | 0,160 |
| WikiE17 | SklearnOnlineVbLda | 0,546 | 0,571 | 0,483 | 0,672 |  | 325,689 | 0,571 | 0,045 | -0,169 | -1,221 | 0,850 | 0,120 | 44,386 | 0,149 |
| WikiE17 | GensimBatchVbLda | 0,614 | 0,653 | 0,557 | 0,716 |  | 311,101 | 0,577 | 0,056 | 0,080 | -1,171 | 0,847 | 0,176 | 46,765 | 0,182 |
| WikiE17 | GensimOnlineVbLda | 0,590 | 0,613 | 0,519 | 0,698 |  | 317,112 | 0,582 | 0,052 | -0,037 | -1,197 | 0,855 | 0,113 | 42,933 | 0,143 |
| WikiE17 | MalletGibbsLda | 0,718 | 0,748 | 0,661 | 0,782 |  | 518,460 | 0,653 | 0,090 | 0,618 | -1,044 | 0,814 | 0,142 | 45,586 | 0,162 |
| WikiE20 | SklearnKmeans | 0,719 | 0,744 | 0,560 | 0,739 |  |  | 0,541 | 0,036 | -0,334 | -1,219 | 0,816 | 0,996 | 72,091 | 0,313 |
| WikiE20 | SklearnLsi | 0,583 | 0,605 | 0,474 | 0,637 |  |  | 0,404 | -0,080 | -3,221 | -2,254 | 0,765 |  |  |  |
| WikiE20 | SklearnNmf | 0,747 | 0,783 | 0,655 | 0,784 |  |  | 0,704 | 0,103 | 0,652 | -1,157 | 0,831 | 0,069 | 40,354 | 0,149 |
| WikiE20 | SklearnPlsa | 0,486 | 0,526 | 0,403 | 0,559 |  |  | 0,483 | 0,011 | -0,506 | -1,286 | 0,778 | 0,178 | 17,049 | 0,142 |
| WikiE20 | SklearnBatchVbLda | 0,637 | 0,680 | 0,593 | 0,752 |  | 335,095 | 0,631 | 0,084 | 0,514 | -1,133 | 0,792 | 0,126 | 44,233 | 0,154 |
| WikiE20 | SklearnOnlineVbLda | 0,532 | 0,557 | 0,477 | 0,677 |  | 352,153 | 0,606 | 0,068 | 0,227 | -1,194 | 0,787 | 0,108 | 43,916 | 0,142 |
| WikiE20 | GensimBatchVbLda | 0,615 | 0,660 | 0,581 | 0,747 |  | 335,038 | 0,618 | 0,078 | 0,408 | -1,148 | 0,786 | 0,154 | 47,196 | 0,176 |
| WikiE20 | GensimOnlineVbLda | 0,584 | 0,610 | 0,523 | 0,704 |  | 341,472 | 0,619 | 0,075 | 0,334 | -1,186 | 0,793 | 0,104 | 43,328 | 0,139 |
| WikiE20 | MalletGibbsLda | 0,704 | 0,734 | 0,644 | 0,777 |  | 500,533 | 0,648 | 0,091 | 0,621 | -1,125 | 0,797 | 0,137 | 44,616 | 0,159 |
| WikiE23 | SklearnKmeans | 0,714 | 0,742 | 0,574 | 0,743 |  |  | 0,553 | 0,043 | -0,201 | -1,190 | 0,746 | 0,992 | 73,259 | 0,292 |
| WikiE23 | SklearnLsi | 0,581 | 0,605 | 0,454 | 0,646 |  |  | 0,404 | -0,085 | -3,154 | -2,248 | 0,689 |  |  |  |
| WikiE23 | SklearnNmf | 0,779 | 0,783 | 0,632 | 0,760 |  |  | 0,677 | 0,091 | 0,541 | -1,185 | 0,800 | 0,068 | 43,986 | 0,145 |
| WikiE23 | SklearnPlsa | 0,588 | 0,601 | 0,431 | 0,577 |  |  | 0,498 | 0,025 | -0,235 | -1,284 | 0,768 | 0,157 | 23,221 | 0,140 |
| WikiE23 | SklearnBatchVbLda | 0,633 | 0,665 | 0,577 | 0,737 |  | 335,963 | 0,609 | 0,076 | 0,475 | -1,160 | 0,775 | 0,125 | 46,755 | 0,152 |
| WikiE23 | SklearnOnlineVbLda | 0,490 | 0,517 | 0,448 | 0,658 |  | 353,786 | 0,565 | 0,053 | 0,034 | -1,232 | 0,768 | 0,104 | 47,343 | 0,139 |
| WikiE23 | GensimBatchVbLda | 0,589 | 0,620 | 0,548 | 0,723 |  | 338,111 | 0,582 | 0,066 | 0,321 | -1,168 | 0,765 | 0,154 | 50,583 | 0,175 |
| WikiE23 | GensimOnlineVbLda | 0,509 | 0,533 | 0,468 | 0,671 |  | 347,175 | 0,576 | 0,056 | 0,087 | -1,226 | 0,772 | 0,097 | 46,465 | 0,133 |
| WikiE23 | MalletGibbsLda | 0,715 | 0,739 | 0,637 | 0,769 |  | 513,662 | 0,621 | 0,084 | 0,619 | -1,111 | 0,778 | 0,139 | 47,378 | 0,160 |
| WikiE25 | SklearnKmeans | 0,696 | 0,719 | 0,535 | 0,718 |  |  | 0,560 | 0,052 | 0,029 | -1,134 | 0,750 | 0,993 | 75,405 | 0,296 |
| WikiE25 | SklearnLsi | 0,587 | 0,600 | 0,427 | 0,601 |  |  | 0,381 | -0,081 | -2,939 | -2,144 | 0,679 |  |  |  |
| WikiE25 | SklearnNmf | 0,723 | 0,742 | 0,570 | 0,723 |  |  | 0,660 | 0,089 | 0,555 | -1,189 | 0,792 | 0,064 | 42,773 | 0,144 |
| WikiE25 | SklearnPlsa | 0,504 | 0,516 | 0,370 | 0,532 |  |  | 0,490 | 0,019 | -0,342 | -1,347 | 0,767 | 0,146 | 27,052 | 0,135 |
| WikiE25 | SklearnBatchVbLda | 0,600 | 0,629 | 0,517 | 0,695 |  | 325,407 | 0,606 | 0,076 | 0,484 | -1,159 | 0,773 | 0,117 | 46,399 | 0,150 |
| WikiE25 | SklearnOnlineVbLda | 0,448 | 0,470 | 0,390 | 0,614 |  | 344,914 | 0,569 | 0,056 | 0,102 | -1,230 | 0,768 | 0,089 | 46,446 | 0,133 |
| WikiE25 | GensimBatchVbLda | 0,574 | 0,607 | 0,503 | 0,691 |  | 327,957 | 0,587 | 0,069 | 0,376 | -1,166 | 0,764 | 0,146 | 49,949 | 0,173 |
| WikiE25 | GensimOnlineVbLda | 0,492 | 0,513 | 0,426 | 0,636 |  | 335,713 | 0,575 | 0,058 | 0,151 | -1,212 | 0,771 | 0,083 | 45,430 | 0,129 |
| WikiE25 | MalletGibbsLda | 0,704 | 0,726 | 0,598 | 0,737 |  | 505,144 | 0,621 | 0,084 | 0,614 | -1,112 | 0,777 | 0,132 | 47,013 | 0,159 |
| WikiE27 | SklearnKmeans | 0,677 | 0,703 | 0,519 | 0,702 |  |  | 0,523 | 0,040 | -0,168 | -1,217 | 0,728 | 0,993 | 77,384 | 0,299 |
| WikiE27 | SklearnLsi | 0,521 | 0,546 | 0,412 | 0,590 |  |  | 0,363 | -0,095 | -3,243 | -2,116 | 0,663 |  |  |  |
| WikiE27 | SklearnNmf | 0,693 | 0,718 | 0,551 | 0,707 |  |  | 0,650 | 0,085 | 0,481 | -1,248 | 0,783 | 0,062 | 44,219 | 0,145 |
| WikiE27 | SklearnPlsa | 0,399 | 0,420 | 0,287 | 0,448 |  |  | 0,501 | 0,027 | -0,185 | -1,320 | 0,765 | 0,093 | 61,257 | 0,120 |
| WikiE27 | SklearnBatchVbLda | 0,592 | 0,621 | 0,508 | 0,689 |  | 333,752 | 0,600 | 0,076 | 0,523 | -1,160 | 0,762 | 0,116 | 48,199 | 0,150 |
| WikiE27 | SklearnOnlineVbLda | 0,430 | 0,450 | 0,378 | 0,609 |  | 354,987 | 0,555 | 0,052 | 0,062 | -1,239 | 0,757 | 0,090 | 50,020 | 0,133 |
| WikiE27 | GensimBatchVbLda | 0,561 | 0,594 | 0,485 | 0,677 |  | 336,378 | 0,578 | 0,068 | 0,401 | -1,161 | 0,753 | 0,144 | 51,980 | 0,174 |
| WikiE27 | GensimOnlineVbLda | 0,460 | 0,481 | 0,415 | 0,632 |  | 346,239 | 0,559 | 0,048 | -0,065 | -1,267 | 0,760 | 0,086 | 51,917 | 0,128 |
| WikiE27 | MalletGibbsLda | 0,676 | 0,699 | 0,577 | 0,724 |  | 538,780 | 0,610 | 0,082 | 0,624 | -1,123 | 0,764 | 0,129 | 49,086 | 0,157 |
| WikiE30 | SklearnKmeans | 0,675 | 0,700 | 0,502 | 0,703 |  |  | 0,527 | 0,046 | 0,021 | -1,126 | 0,725 | 0,993 | 84,673 | 0,300 |
| WikiE30 | SklearnLsi | 0,509 | 0,535 | 0,393 | 0,583 |  |  | 0,367 | -0,082 | -2,901 | -2,071 | 0,667 |  |  |  |
| WikiE30 | SklearnNmf | 0,654 | 0,685 | 0,534 | 0,698 |  |  | 0,626 | 0,083 | 0,506 | -1,252 | 0,773 | 0,058 | 44,362 | 0,142 |
| WikiE30 | SklearnPlsa | 0,421 | 0,434 | 0,313 | 0,476 |  |  | 0,486 | 0,026 | -0,201 | -1,355 | 0,758 | 0,090 | 81,206 | 0,119 |
| WikiE30 | SklearnBatchVbLda | 0,593 | 0,619 | 0,506 | 0,691 |  | 341,603 | 0,582 | 0,072 | 0,474 | -1,181 | 0,752 | 0,110 | 48,575 | 0,148 |
| WikiE30 | SklearnOnlineVbLda | 0,426 | 0,448 | 0,362 | 0,594 |  | 362,669 | 0,562 | 0,059 | 0,225 | -1,253 | 0,756 | 0,084 | 47,759 | 0,131 |
| WikiE30 | GensimBatchVbLda | 0,560 | 0,588 | 0,476 | 0,675 |  | 346,160 | 0,564 | 0,066 | 0,395 | -1,174 | 0,744 | 0,141 | 53,360 | 0,173 |
| WikiE30 | GensimOnlineVbLda | 0,470 | 0,491 | 0,406 | 0,624 |  | 351,546 | 0,568 | 0,061 | 0,254 | -1,238 | 0,757 | 0,078 | 47,113 | 0,125 |
| WikiE30 | MalletGibbsLda | 0,676 | 0,690 | 0,558 | 0,718 |  | 566,418 | 0,597 | 0,079 | 0,607 | -1,151 | 0,757 | 0,124 | 50,013 | 0,156 |
| WikiE35 | SklearnKmeans | 0,610 | 0,644 | 0,432 | 0,658 |  |  | 0,502 | 0,039 | -0,033 | -1,174 | 0,705 | 0,993 | 93,736 | 0,304 |
| WikiE35 | SklearnLsi | 0,441 | 0,480 | 0,326 | 0,519 |  |  | 0,319 | -0,083 | -2,649 | -2,005 | 0,639 |  |  |  |
| WikiE35 | SklearnNmf | 0,586 | 0,624 | 0,442 | 0,634 |  |  | 0,599 | 0,074 | 0,454 | -1,304 | 0,756 | 0,053 | 43,478 | 0,139 |
| WikiE35 | SklearnPlsa | 0,318 | 0,338 | 0,227 | 0,393 |  |  | 0,492 | 0,025 | -0,225 | -1,402 | 0,758 | 0,062 | 138,606 | 0,111 |
| WikiE35 | SklearnBatchVbLda | 0,545 | 0,572 | 0,451 | 0,651 |  | 344,490 | 0,560 | 0,067 | 0,451 | -1,197 | 0,738 | 0,104 | 49,562 | 0,146 |
| WikiE35 | SklearnOnlineVbLda | 0,380 | 0,400 | 0,312 | 0,551 |  | 366,159 | 0,537 | 0,052 | 0,180 | -1,278 | 0,740 | 0,075 | 48,679 | 0,126 |
| WikiE35 | GensimBatchVbLda | 0,515 | 0,546 | 0,434 | 0,643 |  | 350,693 | 0,536 | 0,058 | 0,319 | -1,206 | 0,727 | 0,135 | 55,096 | 0,171 |
| WikiE35 | GensimOnlineVbLda | 0,419 | 0,440 | 0,340 | 0,571 |  | 355,486 | 0,546 | 0,055 | 0,199 | -1,281 | 0,746 | 0,071 | 50,214 | 0,120 |
| WikiE35 | MalletGibbsLda | 0,618 | 0,641 | 0,515 | 0,688 |  | 600,132 | 0,569 | 0,071 | 0,533 | -1,177 | 0,739 | 0,122 | 51,877 | 0,154 |
| WikiE40 | SklearnKmeans | 0,556 | 0,593 | 0,382 | 0,618 |  |  | 0,481 | 0,029 | -0,182 | -1,136 | 0,676 | 0,994 | 101,929 | 0,308 |
| WikiE40 | SklearnLsi | 0,359 | 0,394 | 0,250 | 0,449 |  |  | 0,289 | -0,092 | -2,717 | -2,038 | 0,615 |  |  |  |
| WikiE40 | SklearnNmf | 0,551 | 0,586 | 0,401 | 0,602 |  |  | 0,580 | 0,067 | 0,375 | -1,333 | 0,730 | 0,049 | 45,136 | 0,136 |
| WikiE40 | SklearnPlsa | 0,264 | 0,274 | 0,180 | 0,338 |  |  | 0,474 | 0,023 | -0,233 | -1,420 | 0,740 | 0,044 | 222,723 | 0,104 |
| WikiE40 | SklearnBatchVbLda | 0,501 | 0,526 | 0,401 | 0,611 |  | 357,112 | 0,544 | 0,061 | 0,411 | -1,229 | 0,714 | 0,101 | 51,749 | 0,144 |
| WikiE40 | SklearnOnlineVbLda | 0,340 | 0,355 | 0,280 | 0,522 |  | 381,577 | 0,510 | 0,042 | 0,042 | -1,305 | 0,712 | 0,091 | 70,748 | 0,129 |
| WikiE40 | GensimBatchVbLda | 0,482 | 0,508 | 0,396 | 0,611 |  | 361,501 | 0,526 | 0,055 | 0,320 | -1,216 | 0,705 | 0,135 | 57,648 | 0,171 |
| WikiE40 | GensimOnlineVbLda | 0,372 | 0,388 | 0,299 | 0,536 |  | 368,152 | 0,519 | 0,044 | 0,049 | -1,301 | 0,719 | 0,069 | 63,969 | 0,118 |
| WikiE40 | MalletGibbsLda | 0,594 | 0,614 | 0,475 | 0,654 |  | 638,381 | 0,554 | 0,067 | 0,514 | -1,183 | 0,712 | 0,119 | 53,808 | 0,153 |
| WikiE45 | SklearnKmeans | 0,519 | 0,552 | 0,347 | 0,582 |  |  | 0,485 | 0,037 | 0,040 | -1,160 | 0,670 | 0,995 | 107,294 | 0,311 |
| WikiE45 | SklearnLsi | 0,350 | 0,375 | 0,230 | 0,425 |  |  | 0,281 | -0,091 | -2,680 | -2,073 | 0,608 |  |  |  |
| WikiE45 | SklearnNmf | 0,499 | 0,526 | 0,335 | 0,542 |  |  | 0,577 | 0,065 | 0,356 | -1,349 | 0,720 | 0,045 | 46,276 | 0,133 |
| WikiE45 | SklearnPlsa | 0,245 | 0,256 | 0,171 | 0,330 |  |  | 0,476 | 0,021 | -0,291 | -1,464 | 0,733 | 0,036 | 303,283 | 0,101 |
| WikiE45 | SklearnBatchVbLda | 0,470 | 0,498 | 0,360 | 0,581 |  | 357,950 | 0,547 | 0,064 | 0,459 | -1,236 | 0,707 | 0,090 | 52,044 | 0,140 |
| WikiE45 | SklearnOnlineVbLda | 0,293 | 0,306 | 0,235 | 0,487 |  | 385,207 | 0,505 | 0,040 | 0,004 | -1,347 | 0,705 | 0,077 | 72,495 | 0,123 |
| WikiE45 | GensimBatchVbLda | 0,444 | 0,470 | 0,342 | 0,571 |  | 365,896 | 0,528 | 0,057 | 0,368 | -1,228 | 0,696 | 0,126 | 59,712 | 0,169 |
| WikiE45 | GensimOnlineVbLda | 0,322 | 0,339 | 0,252 | 0,500 |  | 372,709 | 0,510 | 0,036 | -0,144 | -1,355 | 0,710 | 0,062 | 74,487 | 0,114 |
| WikiE45 | MalletGibbsLda | 0,540 | 0,564 | 0,408 | 0,609 |  | 665,411 | 0,560 | 0,070 | 0,566 | -1,209 | 0,707 | 0,111 | 55,375 | 0,149 |
| WikiE50 | SklearnKmeans | 0,504 | 0,539 | 0,324 | 0,573 |  |  | 0,473 | 0,033 | -0,040 | -1,176 | 0,654 | 0,994 | 112,236 | 0,311 |
| WikiE50 | SklearnLsi | 0,327 | 0,356 | 0,213 | 0,405 |  |  | 0,263 | -0,087 | -2,393 | -2,067 | 0,585 |  |  |  |
| WikiE50 | SklearnNmf | 0,477 | 0,503 | 0,317 | 0,526 |  |  | 0,568 | 0,063 | 0,364 | -1,386 | 0,711 | 0,039 | 43,861 | 0,131 |
| WikiE50 | SklearnPlsa | 0,239 | 0,250 | 0,177 | 0,336 |  |  | 0,471 | 0,022 | -0,257 | -1,486 | 0,723 | 0,029 | 401,661 | 0,098 |
| WikiE50 | SklearnBatchVbLda | 0,459 | 0,486 | 0,354 | 0,581 |  | 348,748 | 0,536 | 0,061 | 0,443 | -1,239 | 0,696 | 0,085 | 51,116 | 0,139 |
| WikiE50 | SklearnOnlineVbLda | 0,261 | 0,273 | 0,228 | 0,493 |  | 379,861 | 0,479 | 0,030 | -0,133 | -1,378 | 0,688 | 0,105 | 118,004 | 0,133 |
| WikiE50 | GensimBatchVbLda | 0,434 | 0,455 | 0,323 | 0,553 |  | 365,881 | 0,519 | 0,052 | 0,350 | -1,240 | 0,685 | 0,120 | 59,709 | 0,155 |
| WikiE50 | GensimOnlineVbLda | 0,319 | 0,319 | 0,247 | 0,486 |  | 372,708 | 0,507 | 0,035 | -0,146 | -1,370 | 0,707 | 0,052 | 74,482 | 0,101 |
| WikiE50 | MalletGibbsLda | 0,528 | 0,552 | 0,402 | 0,607 |  | 654,398 | 0,551 | 0,067 | 0,537 | -1,205 | 0,696 | 0,105 | 54,981 | 0,148 |
| WikiD55 | SklearnKmeans | 0,467 | 0,503 | 0,303 | 0,545 |  |  | 0,474 | 0,031 | -0,057 | -1,253 | 0,648 | 0,997 | 129,575 | 0,339 |
| WikiD55 | SklearnLsi | 0,296 | 0,324 | 0,191 | 0,373 |  |  | 0,264 | -0,073 | -2,190 | -1,989 | 0,600 |  |  |  |
| WikiD55 | SklearnNmf | 0,433 | 0,454 | 0,283 | 0,502 |  |  | 0,560 | 0,061 | 0,352 | -1,436 | 0,719 | 0,033 | 39,537 | 0,129 |
| WikiD55 | SklearnPlsa | 0,305 | 0,318 | 0,234 | 0,421 |  |  | 0,507 | 0,050 | 0,049 | -1,661 | 0,744 | 0,012 | 696,385 | 0,090 |
| WikiD55 | SklearnBatchVbLda | 0,433 | 0,458 | 0,331 | 0,565 |  | 355,328 | 0,532 | 0,060 | 0,445 | -1,301 | 0,706 | 0,078 | 48,101 | 0,136 |
| WikiD55 | SklearnOnlineVbLda | 0,261 | 0,270 | 0,218 | 0,479 |  | 383,270 | 0,492 | 0,043 | 0,051 | -1,378 | 0,711 | 0,072 | 76,073 | 0,122 |
| WikiD55 | GensimBatchVbLda | 0,411 | 0,432 | 0,310 | 0,542 |  | 368,017 | 0,519 | 0,059 | 0,363 | -1,284 | 0,692 | 0,113 | 56,957 | 0,168 |
| WikiD55 | GensimOnlineVbLda | 0,291 | 0,306 | 0,231 | 0,476 |  | 371,269 | 0,495 | 0,034 | -0,141 | -1,405 | 0,720 | 0,051 | 80,679 | 0,110 |
| WikiD55 | MalletGibbsLda | 0,510 | 0,533 | 0,379 | 0,589 |  | 660,316 | 0,550 | 0,074 | 0,545 | -1,265 | 0,702 | 0,112 | 54,834 | 0,151 |
| WikiD60 | SklearnKmeans | 0,425 | 0,455 | 0,268 | 0,512 |  |  | 0,457 | 0,028 | -0,088 | -1,314 | 0,634 | 0,995 | 124,066 | 0,319 |
| WikiD60 | SklearnLsi | 0,260 | 0,279 | 0,159 | 0,335 |  |  | 0,259 | -0,072 | -1,995 | -1,859 | 0,571 |  |  |  |
| WikiD60 | SklearnNmf | 0,389 | 0,404 | 0,244 | 0,459 |  |  | 0,541 | 0,057 | 0,331 | -1,403 | 0,691 | 0,033 | 42,014 | 0,127 |
| WikiD60 | SklearnPlsa | 0,357 | 0,374 | 0,271 | 0,491 |  |  | 0,540 | 0,058 | 0,347 | -1,346 | 0,703 | 0,026 | 427,255 | 0,109 |
| WikiD60 | SklearnBatchVbLda | 0,397 | 0,419 | 0,292 | 0,529 |  | 361,895 | 0,522 | 0,058 | 0,447 | -1,255 | 0,676 | 0,073 | 48,878 | 0,134 |
| WikiD60 | SklearnOnlineVbLda | 0,244 | 0,259 | 0,192 | 0,448 |  | 386,661 | 0,492 | 0,044 | 0,216 | -1,310 | 0,675 | 0,086 | 103,889 | 0,128 |
| WikiD60 | GensimBatchVbLda | 0,374 | 0,398 | 0,281 | 0,525 |  | 370,137 | 0,501 | 0,051 | 0,359 | -1,247 | 0,662 | 0,109 | 59,276 | 0,165 |
| WikiD60 | GensimOnlineVbLda | 0,260 | 0,278 | 0,203 | 0,457 |  | 369,821 | 0,482 | 0,032 | -0,138 | -1,389 | 0,685 | 0,059 | 118,837 | 0,113 |
| WikiE60 | MalletGibbsLda | 0,483 | 0,505 | 0,342 | 0,562 |  | 666,220 | 0,538 | 0,065 | 0,552 | -1,214 | 0,676 | 0,097 | 53,663 | 0,144 |
| WikiD65 | SklearnKmeans | 0,414 | 0,446 | 0,266 | 0,507 |  |  | 0,439 | 0,010 | -0,107 | -1,163 | 0,634 | 0,997 | 136,339 | 0,340 |
| WikiD65 | SklearnLsi | 0,248 | 0,277 | 0,154 | 0,326 |  |  | 0,251 | -0,076 | -1,998 | -1,963 | 0,572 |  |  |  |
| WikiD65 | SklearnNmf | 0,381 | 0,387 | 0,235 | 0,452 |  |  | 0,523 | 0,042 | 0,323 | -1,491 | 0,704 | 0,027 | 38,091 | 0,124 |
| WikiD65 | SklearnPlsa | 0,352 | 0,361 | 0,253 | 0,480 |  |  | 0,523 | 0,053 | 0,338 | -1,703 | 0,725 | 0,008 | 1049,848 | 0,088 |
| WikiD65 | SklearnBatchVbLda | 0,384 | 0,408 | 0,272 | 0,526 |  | 361,881 | 0,515 | 0,057 | 0,435 | -1,313 | 0,689 | 0,064 | 44,047 | 0,131 |
| WikiD65 | SklearnOnlineVbLda | 0,236 | 0,240 | 0,179 | 0,440 |  | 386,659 | 0,472 | 0,043 | 0,208 | -1,407 | 0,691 | 0,068 | 100,366 | 0,118 |
| WikiD65 | GensimBatchVbLda | 0,366 | 0,386 | 0,276 | 0,524 |  | 370,119 | 0,498 | 0,046 | 0,354 | -1,288 | 0,672 | 0,101 | 55,540 | 0,165 |
| WikiD65 | GensimOnlineVbLda | 0,258 | 0,268 | 0,185 | 0,451 |  | 369,806 | 0,465 | 0,028 | -0,150 | -1,453 | 0,698 | 0,045 | 98,329 | 0,106 |
| WikiD65 | MalletGibbsLda | 0,482 | 0,504 | 0,339 | 0,556 |  | 666,211 | 0,530 | 0,059 | 0,548 | -1,275 | 0,685 | 0,100 | 53,187 | 0,147 |
| WikiD70 | SklearnKmeans | 0,401 | 0,430 | 0,259 | 0,506 |  |  | 0,426 | 0,008 | -0,122 | -1,182 | 0,622 | 0,997 | 142,632 | 0,341 |
| WikiD70 | SklearnLsi | 0,247 | 0,268 | 0,146 | 0,308 |  |  | 0,235 | -0,077 | -2,004 | -1,898 | 0,563 |  |  |  |
| WikiD70 | SklearnNmf | 0,380 | 0,375 | 0,226 | 0,435 |  |  | 0,519 | 0,036 | 0,323 | -1,492 | 0,696 | 0,025 | 38,773 | 0,123 |
| WikiD70 | SklearnPlsa | 0,350 | 0,357 | 0,233 | 0,465 |  |  | 0,517 | 0,036 | 0,325 | -1,726 | 0,716 | 0,006 | 1223,228 | 0,087 |
| WikiD70 | SklearnBatchVbLda | 0,370 | 0,408 | 0,266 | 0,516 |  | 361,866 | 0,506 | 0,055 | 0,420 | -1,321 | 0,679 | 0,059 | 43,762 | 0,129 |
| WikiD70 | SklearnOnlineVbLda | 0,223 | 0,231 | 0,165 | 0,424 |  | 386,649 | 0,456 | 0,030 | 0,205 | -1,415 | 0,682 | 0,067 | 111,692 | 0,118 |
| WikiD70 | GensimBatchVbLda | 0,357 | 0,371 | 0,274 | 0,505 |  | 370,113 | 0,495 | 0,041 | 0,348 | -1,294 | 0,662 | 0,096 | 56,646 | 0,164 |
| WikiD70 | GensimOnlineVbLda | 0,240 | 0,259 | 0,181 | 0,449 |  | 369,805 | 0,452 | 0,025 | -0,164 | -1,465 | 0,695 | 0,038 | 93,291 | 0,104 |
| WikiD70 | MalletGibbsLda | 0,463 | 0,488 | 0,320 | 0,551 |  | 667,209 | 0,513 | 0,045 | 0,529 | -1,288 | 0,676 | 0,093 | 52,309 | 0,145 |
| WikiE75 | SklearnKmeans | 0,391 | 0,414 | 0,242 | 0,499 |  |  | 0,388 | 0,004 | -0,139 | -1,206 | 0,590 | 0,994 | 142,605 | 0,337 |
| WikiE75 | SklearnLsi | 0,243 | 0,244 | 0,129 | 0,277 |  |  | 0,224 | -0,109 | -2,038 | -1,903 | 0,537 |  |  |  |
| WikiE75 | SklearnNmf | 0,362 | 0,344 | 0,207 | 0,434 |  |  | 0,515 | 0,005 | 0,313 | -1,524 | 0,672 | 0,007 | 38,766 | 0,120 |
| WikiE75 | SklearnPlsa | 0,327 | 0,330 | 0,233 | 0,445 |  |  | 0,517 | 0,036 | 0,293 | -1,745 | 0,687 | 0,002 | 1223,223 | 0,078 |
| WikiE75 | SklearnBatchVbLda | 0,346 | 0,394 | 0,236 | 0,485 |  | 361,828 | 0,477 | 0,054 | 0,397 | -1,339 | 0,678 | 0,056 | 43,759 | 0,121 |
| WikiE75 | SklearnOnlineVbLda | 0,222 | 0,213 | 0,163 | 0,412 |  | 386,631 | 0,427 | 0,004 | 0,176 | -1,420 | 0,656 | 0,055 | 111,662 | 0,081 |
| WikiE75 | GensimBatchVbLda | 0,350 | 0,335 | 0,251 | 0,482 |  | 370,074 | 0,460 | 0,003 | 0,311 | -1,320 | 0,641 | 0,084 | 56,638 | 0,133 |
| WikiE75 | GensimOnlineVbLda | 0,212 | 0,244 | 0,163 | 0,442 |  | 369,798 | 0,445 | -0,001 | -0,196 | -1,470 | 0,663 | 0,031 | 93,269 | 0,102 |
| WikiE75 | MalletGibbsLda | 0,425 | 0,467 | 0,312 | 0,533 |  | 669,192 | 0,478 | 0,023 | 0,524 | -1,237 | 0,658 | 0,080 | 50,746 | 0,139 |
